# Inequalities and risk factors analysis in prevalence and management of hypertension in India and Nepal: a national and subnational study

**DOI:** 10.1186/s12889-020-09450-6

**Published:** 2020-09-03

**Authors:** Santosh Kumar Rauniyar, Md. Mizanur Rahman, Md. Shafiur Rahman, Sarah Krull Abe, Shuhei Nomura, Kenji Shibuya

**Affiliations:** 1 Department of Global Health Policy, Graduate School of Medicine, The University of Tokyo, 7-3-1, Hongo, Bunkyo-ku, Tokyo, 113-0033 Japan; 2 Epidemiology and Prevention Group, Center for Public Health Sciences, National Cancer Center, Tokyo, Japan; 3 Institute for Global Health Policy Research, Bureau of International Health Cooperation, National Center for Global Health and Medicine, Tokyo, Japan; 4 Institute for Population Health Science, King’s College London, London, UK

**Keywords:** Inequality in management of hypertension, Hypertension, Blood pressure, Non-communicable diseases

## Abstract

**Background:**

Hypertension is one of the leading risk factors for cardiovascular diseases in India and Nepal. Socio-economic disparity in these two countries has created wide gap in management of hypertension. However, inequalities in prevalence and management (awareness, treatment, and control) of hypertension is poorly assessed. This study analyzes the risk factors associated with prevalence and management of hypertension in India and Nepal and assesses the wealth-and education-based inequalities in them.

**Methods:**

This study used data from the Demographic and Health Survey; a cross-sectional survey conducted between January 2015 to December 2016 in India and June 2016 to January 2017 in Nepal. A total of 787,713 individuals in India and 14,454 individuals in Nepal aged between 15 and 49 years were included in the study. Respondents were classified as being hypertensive if their systolic blood pressure (SBP) readings were at least 140 mmHg or diastolic blood pressure (DBP) readings were at least 90 mmHg, or if they reported currently taking anti-hypertensive medication. Multilevel logistic regression models with random intercepts at household-and community-levels were used to identify the risk factors associated with prevalence and management of hypertension. For inequality assessment, slope index and relative index of inequalities in prevalence and management of hypertension were estimated.

**Results:**

Overall prevalence of hypertension in India and Nepal were 11.4% (95% confidence interval (CI), 11.4–11.5) and 19.6% (95% CI, 18.9–20.2), respectively. Less than one-third of the hypertensive population received treatment and below 20% among them had their blood pressure controlled. In both countries, wealth-and education-based inequalities in awareness, treatment, and control of hypertension were significantly high in urban and rural areas.

**Conclusion:**

Wealth- and education**-**based inequalities in prevalence and management of hypertension were high among different socio-economic groups at national and sub-national levels. Tailored strategies are required to effectively manage hypertension in different regions by considering socio-economic and demographic factors.

## Background

Hypertension is one of the major global health risks for cardiovascular diseases (CVDs), kidney disease, and other complications [1, 2]. In 2017, CVDs caused 17.8 million deaths worldwide, accounting for 42% of total mortality due to non-communicable diseases (NCDs); 58% of them had complication of hypertension [3]. Accorinidng to WHO estimate in 2010, almost 1 billion people suffered due to raised blood pressure which was around 40% in low- and middle- income countries (LMICs) and 35% in high-income countires (HICs) [2, 4]. Thus, the Global Action Plan for the Prevention and Control of NCDs was adopted by the World Health Assembly in 2013, targeted to reduce the global prevalence of raised blood pressure (BP) by 25% by 2020 (relative to its 2010 level) [4]. However, despite the global efforts, some studies suggest that prevalence of hypertension among adults is predicted to increase from 26.4% in 2000 to 29.2% in 2025 [5].

Prevalence of hypertension and its related disease burden are notably increasing in most LMICs including southeast Asia, where it affected more than 35% of adults in 2013 [2]. As with other LMICs, hypertension is one of the leading risk factors for health loss including both premature deaths and disabilities in India and Nepal [6, 7]. In 2017, about 7.9 and 6.7% of total disability adjusted life years (DALYs) were attributed to high systolic blood pressure in India and Nepal, respectively with an upward trend since 1990 [8].

Early diagnosis and effective treatment of hypertension are key strategies to reduce disabilities and mortality related to CVDs and other chronic diseases [9, 10]. However, there are no studies that evaluates the availability and affordability of screening and treatment services for hypertension at subnational levels in India and Nepal. It is well known fact that inequality in access to health care services due to socio-demographic and economic factors leads to higher disparity in health outcome [11, 12]. Hence, prevailing health problems cannot be tackled effectively, if disparities in availability and accessibility of health care services are not thoroughly evaluated and intervened within the communities [13]. Therefore, measuring inequalities in prevalence, awareness, treatment, and control of hypertension at national and subnational levels is critical to understand the existing disparities in prevalence and management of hypertension within the communities. Further, this critical assessment will help to formulate tailored strategies and policies to effectively manage hypertension.

Few previous studies demonstrated the inequalities in prevalence and management of hypertension in LMICs [14, 15]. Nonetheless, these studies were mostly based on national-level estimates and did not measure inequalities at sub-national levels, which are more relevant to the needs of policy makers and administrators. Therefore, our study attempts to measure inequalities in prevalence and management of hypertension at national and sub-national levels and assess the risk factors associated with it using a nationally representative sample from Nepal and India.

## Method

### Sources of data

We used recently available data from the National Family Health Survey 2015–16 (NFHS-4) in India and Nepal Demographic and Health Survey 2015–16 (NDHS) in Nepal to analyze the risk factors and the inequalities in prevalence and management of hypertension. Both surveys were nationally representative population-based cross-sectional household surveys that included participants aged 15–49 years. Due to limited data availability population aged 50 years and above were not included in this study. However, inclusion of population aged 15–49 years emphasizes the burden of hypertension in younger age group in both countries. Data were collected from January 2015 to December 2016 in India and June 2016 to January 2017 in Nepal. NFHS-4 selected 616,346 occupied households and interviewed 601,509 households with response rate of 98.0%. NDHS interviewed 11,203 households out of 11,472 occupied households with response rate of 99.0%. Both surveys used multistage stratified cluster sampling design method to select sample. Out of total population of 1.3 billion in India [16], 687,230 women and 100,483 men aged 15–49 years were included in the study. In Nepal, out of total population of 29.3 million [17], 8488 women and 5966 men aged 15–49 years were included. Further details for data collection are provided in Supplementary Method 1&2, p68. Detailed methodology for each survey is available elsewhere [18, 19].

### Outcome variables

Prevalence, awareness, treatment, and control of hypertension were the main outcome variables in this study. Respondents were classified as being hypertensive if their systolic blood pressure (SBP) readings were at least 140 mm Hg (mmHg) or diastolic blood pressure (DBP) readings were at least 90 mmHg, or if they reported currently taking anti-hypertensive medication [20]. SBP and DBP readings were measured three times within five-minute intervals. Management of hypertension includes awareness, treatment, and control of hypertension. Awareness of hypertension was defined as self-reported case of hypertension previously diagnosed by a doctor or other health professionals [20]. Treatment of hypertension was defined as self-reported use of prescribed antihypertensive medication [20], and control of hypertension was defined as receiving antihypertensive medication and having an average SBP below 140 mmHg or DBP below 90 mmHg [20]. All the measurements were taken by the trained field staffs using standardized blood pressure monitors. Details are provided in Supplementary Method 3 p73.

### Covariates

We selected predictor variables for the analyses based on previous literatures on hypertension and its management [7, 21]. Covariates were stratified into three levels: individual-, household-, and community-levels. Individual-level variables included were; age, sex, educational status, body mass index (BMI), alcohol and tobacco consumption, and marital status. In both countries, participants aged 15–49 years of age were included in the study. Household-level variables included were availability of iodized salt and household wealth quintile. Community-level variables were region of residence, place of residence, and further stratified by province and states, respectively. Details of covariates are provided in Supplementary Method 3 p73.

### Statistical analysis

We used descriptive statistics and frequency distributions to describe participants’ demographic and socio-economic characteristics. Prevalence, awareness, treatment, and control of hypertension were calculated by measuring proportion. To investigate the relationship between covariates and outcome variables, we employed multilevel logistic regression models with random intercept at household-and community-levels [22, 23]. Since NFHS-4 and NDHS used a multi-stage cluster sampling method (described above), the obtained data were nested in multiple categories (for example primary sampling units, states/provinces, and households). Thus, to account for the cluster level effect (within-cluster correlation), multilevel logistic regression was performed in this study [23]. Further, both descriptive and multilevel regression analyses were adjusted to complex survey design using sample weight.

To assess the inequalities in prevalence and management of hypertension, we estimated slope index of inequality (SII), relative index of inequality (RII), concentration index (CIX), and decomposition of concentration index at national and sub-national levels. Inequality analyses were performed based on household wealth index and education level. SII is a weighted measure of inequality that represents the absolute difference in estimated values of a health indicator between the most advantaged and disadvantaged, while taking into consideration all other subgroups using an appropriate regression model. Whereas, RII is a weighted measure of inequality that shows health gradient across multiple subgroups with natural ordering, on a relative scale. It is a ratio of estimated value of a health indicator in the most-advantaged to the most-disadvantaged (or vice-versa) while taking into consideration all other subgroups [24, 25]. Similarly, CIX is a measure of inequality in a health-related variable over distribution of another variable of interest such as household wealth quintile or education. CIX is defined in reference to the concentration curve (CC) which graphs the cumulative proportion of health-related variable against the cumulative proportion of the population ranked by variable of interest. It quantifies the degree of inequality in a specific health variable [9, 26–28]. Further, we decomposed concentration index to see the contribution of other socio-economic and demographic factors towards the concentration of prevalence and management of hypertension [26, 27, 29]. Detail of statistical analyses is provided in Supplementary Methods 4 p76. Statistical analyses were performed using Stata version 15.1/SE.

## Results

### Sample characteristics

**Fig. 1:**
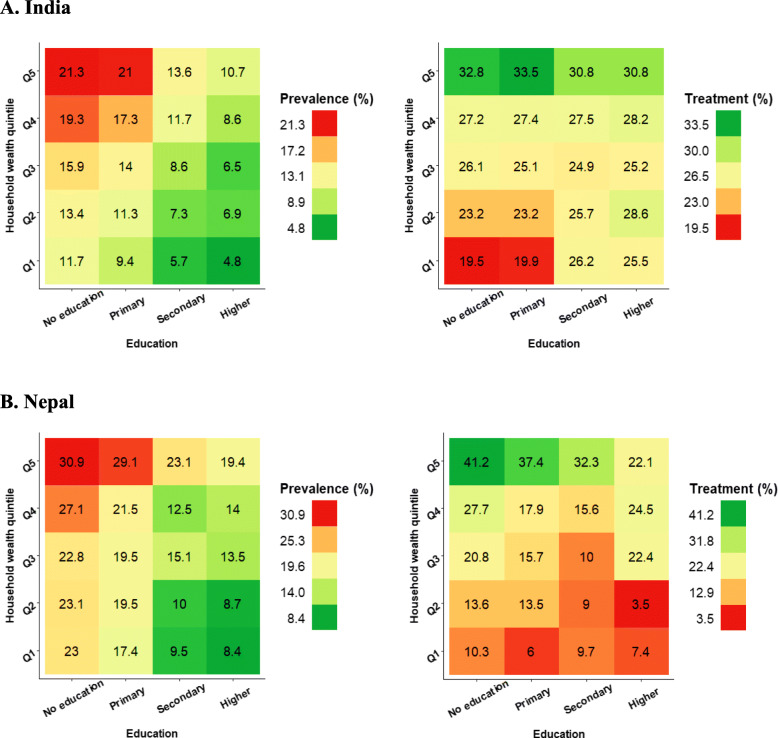
Prevalence and treatment of hypertension by household wealth quintile and education in India and Nepal, 2016. * Q1 = Poorest quintile, Q5 = Richest quintile * All the figures were generated in R programming software

**Fig. 2:**
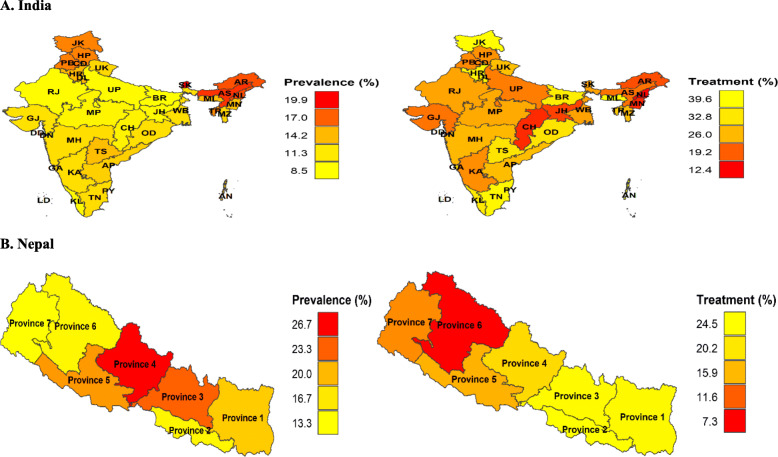
Prevalence and treatment of hypertension at subnational levels in India and Nepal, 2016. AN, Andaman and Nicobar Island; AP, Andhra Pradesh; AR, Arunachal Pradesh; AS, Assam; BR, Bihar; CD, Chandigarh; CH, Chhattisgarh; DN, Dadra and Nagar Haveli; DD, Daman and Diu; GA, Goa; GJ, Gujarat; HR, Haryana; HP, Himachal Pradesh; JK, Jammu & Kashmir; JH, Jharkhand; KA, Karnataka; KL, Kerala; LD, Lakshadweep; MP, Madhya Pradesh; MH, Maharashtra; MN, Manipur; ML, Meghalaya; MZ, Mizoram; NL, Nagaland; DL, New Delhi; OD, Odisha; PY, Puducherry; PB, Punjab; RJ, Rajasthan; SK, Sikkim; TN, Tamil Nadu; TR, Tripura; UP, Uttar Pradesh; UK, Uttarakhand; WB, West Bengal; TS, Telangana * All the choropleth maps were generated in R programming software using spatial data from the DHS Spatial Data Repository. Permission to reproduce the map was obtained from DHS Program

**Fig. 3:**
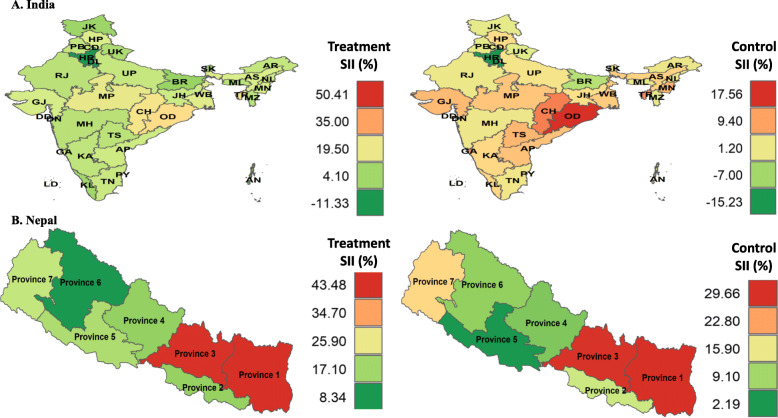
Wealth-based SII in treatment and control of hypertension in India and Nepal, 2016. *SII = slope index of inequality; AN, Andaman and Nicobar Island; AP, Andhra Pradesh; AR, Arunachal Pradesh; AS, Assam; BR, Bihar; CD, Chandigarh; CH, Chhattisgarh; DN, Dadra and Nagar Haveli; DD, Daman and Diu; GA, Goa; GJ, Gujarat; HR, Haryana; HP, Himachal Pradesh; JK, Jammu & Kashmir; JH, Jharkhand; KA, Karnataka; KL, Kerala; LD, Lakshadweep; MP, Madhya Pradesh; MH, Maharashtra; MN, Manipur; ML, Meghalaya; MZ, Mizoram; NL, Nagaland; DL, New Delhi; OD, Odisha; PY, Puducherry; PB, Punjab; RJ, Rajasthan; SK, Sikkim; TN, Tamil Nadu; TR, Tripura; UP, Uttar Pradesh; UK, Uttarakhand; WB, West Bengal; TS, Telangana; * All the choropleth maps were generated in R programming software using spatial data from the DHS Spatial Data Repository. Permission to reproduce the map was obtained from DHS Program.

Table 1 shows the sample characteristics of the selected population in India and Nepal. The overall prevalence of hypertension in India and Nepal was 11.4% (95% confidence interval [CI] 11.4–11.5) and 19.6% (18.9–20.2) respectively. Among the hypertensive population, 30.3% (30.0–30.6) in India and 40.0% (38.2–41.8) in Nepal were aware of their condition, 26.4% (26.2–26.7) and 20.2% (18.8–21.7), respectively, were receiving treatment. Only 17.8% (17.5–18.0) in India and 10.4% (9.3–11.6) in Nepal under the treatment had their blood pressure controlled. Detailed statistics are provided in Figs. 1, 2, 3, Supplementary Tables 1–2 p1, and Supplementary Figs. 1–10 p35.

**Table 1:** Characteristics of selected participants in India and Nepal, 2016 (number, %)

Characteristics	India		Nepal	
	Female	Male	Female	Male
Sample size	682,624 (88.6)	99,217 (11.4)	8435 (58.2)	6059 (41.8)
Age group				
15–24	238,367 (34.9)	33,882 (34.1)	2436 (28.9)	1584 (16.1)
25–34	206,687 (30.3)	29,355 (29.6)	1932 (22.9)	1089 (18.0)
35–44	165,034 (24.2)	24,894 (25.1)	1475 (17.5)	1005 (16.6)
45–49	72,536 (10.6)	11,086 (11.2)	522 (6.2)	427 (7.05)
Above 49	NA	NA	2069 (24.5)	1953 (32.2)
Educational status				
No formal education	180,499 (26.5)	11,098 (11.2)	4009 (47.6)	1449 (23.9)
Primary education	91,600 (13.4)	12,034 (12.2)	1158 (13.7)	1231 (20.3)
Secondary education	324,407 (47.6)	58,228 (58.8)	2321 (27.5)	2340 (38.7)
Higher education	84,942 (12.5)	17,628 (17.8)	941 (1.2)	1032 (17.1)
Marital status				
Never married	153,692 (22.5)	37,212 (37.5)	1339 (15.9)	1369 (22.6)
Married	500,911 (73.4)	60,785 (61.3)	6241 (74.0)	4387 (72.4)
Widowed	20,813 (3.1)	583 (0.6)	767 (9.1)	260 (4.3)
Divorced	2200 (0.3)	239 (0.2)	85 (1.0)	42 (0.7)
Not Living together	4966 (0.7)	393 (0.4)	NA	NA
Tobacco consumption				
No	635,761 (93.1)	54,645 (55.1)	8148 (96.6)	4896 (80.8)
Yes	46,862 (6.9)	44,574 (44.9)	287 (3.4)	1163 (19.2)
Alcohol consumption				
No	73,279 (98.0)	70,020 (70.6)	8383 (99.4)	5903 (97.4)
Yes	1515 (2.0)	29,197 (29.4)	52 (0.6)	155 (2.6)
Body mass index				
Underweight	152,918 (22.4)	19,992 (20.2)	1577 (18.8)	1079 (17.9)
Normal	389,263 (57.1)	60,231 (60.8)	4987 (59.3)	3939 (65.5)
Overweight	104,808 (15.4)	15,717 (15.9)	1420 (16.9)	869 (14.4)
Obese	34,915 (5.1)	3108 (3.1)	415 (4.9)	130 (2.2)
Wealth quintile				
Q1 (Poorest)	122,110 (17.9)	14,809 (14.9)	1535 (18.2)	1028 (17.0)
Q2	134,859 (19.8)	18,896 (19.1)	1758 (20.8)	1208 (20.0)
Q3	141,047 (20.7)	21,209 (21.4)	1831 (21.7)	1291 (21.3)
Q4	143,992 (21.1)	21,870 (22.0)	1608 (19.1)	1213 (20.0)
Q5 (Richest)	140,614 (20.6)	22,431 (22.6)	1704 (20.2)	1316 (21.7)
Availability of iodine				
Iodine present	636,778 (93.4)	93,097 (93.9)	7970 (94.6)	5737 (94.8)
No iodine	43,783 (6.4)	5703 (5.8)	409 (4.9)	272 (4.5)
No salt in house	1392 (0.2)	318 (0.3)	49 (0.6)	43 (0.7)
Place of residence				
Urban	232,326 (34.0)	37,053 (37.4)	5153 (61.1)	3740 (61.7)
Rural	450,297 (66.0)	62,163 (62.7)	3281 (38.9)	2318 (38.3)

### Factors associated with prevalence and management of hypertension

Supplementary Table 3 and Supplementary Table 4 shows the risk factors associated with prevalence, awareness, treatment, and control of hypertension in India and Nepal. After adjusting for covariates, the odds of being hypertensive was higher in men compared to women in both countries (Odds ratio [OR] 1.47, 95% CI, 1.41–1.52 for India and 1.96 (1.59–2.44) for Nepal). However, the odds for awareness, treatment, and control were lower in men compared to women in both countries. In India, the odds of being hypertensive in the wealthy and poorest population were similar, however in case of Nepal these results were not statistically significant. But, the OR for treatment of hypertension were 1.58 (1.41–1.77) and 7.86 (1.92–32.11) and for control were 1.30 (1.14–1.48) and 9.48 (1.11–80.81) in the richest quintile in reference to the poorest quintile in India and Nepal, respectively. In both countries, urban residents had higher odds of being aware and having treatment. In India, odds of having the blood pressure controlled was higher in urban residents compared to the rural residents, however in Nepal this result was not statistically significant.

### Wealth-based inequality in prevalence and management of hypertension

Table 2 provides the summary results of wealth-based inequality in prevalence and management of hypertension in India and Nepal. Prevalence of hypertension was 5.2 (95% CI 4.7–5.7) and 7.1 (2.9–11.4) percentage points higher in affluent populations compared to the disadvantaged ones in India and Nepal. Similarly, inequalities in awareness, treatment, and control were substantially high and concentrated towards the wealthy populations in both countries (Fig. 3). Both in urban and rural residences in India, affluent groups had higher percentage points of hypertension prevalence, awareness, treatment, and control than the disadvantaged groups. Unlike in India, absolute inequalities in prevalence, treatment, and control in Nepal were higher in urban residence compared to rural. Wealthy urban population in Nepal had higher prevalence, awareness, treatment, and control than the poorer and poorest population. However, in rural residence, poorest population had higher prevalence of hypertension than the affluent ones.

**Table 2:** Wealth based inequalities in prevalence and management of hypertension in India and Nepal, 2016

Categorization	Mean		SII (95% CI)	RII (95% CI)	CIX*100 (95% CI)
	Q1 (95% CI)	Q5 (95% CI)			
**India**					
**Prevalence (national)**	9.4 (9.3–9.6)	13.4 (13.2–13.5)	5.22 (4.77–5.67)	1.53 (1.48–1.59)	7.28 (6.75–7.81)
Urban	10.4 (9.7–11.1)	13.1 (13.0–13.3)	2.63 (1.74–3.52)	1.22 (1.14–1.30)	3.79 (2.71–4.88)
Rural	9.4 (9.2–9.5)	14.0 (13.7–14.3)	4.71 (4.27–5.15)	1.50 (1.45–1.56)	6.21 (5.67–6.75)
**Awareness (national)**	19.2 (18.5–19.9)	37.7 (37.0–38.3)	20.25 (18.47–22.03)	1.95 (1.84–2.07)	11.91 (11.08–12.74)
Urban	19.1 (16.2–21.9)	37.9 (37.1–38.6)	12.20 (8.77–15.64)	1.42 (1.28–1.56)	8.43 (6.73–10.12)
Rural	19.2 (18.5–19.9)	37.1 (35.9–38.3)	18.82 (16.90–20.75)	1.98 (1.84–2.12)	10.48 (9.62–11.34)
**Treatment (national)**	20.8 (20.1–21.5)	31.2 (30.6–31.8)	11.68 (10.01–13.35)	1.55 (1.45–1.64)	7.25 (6.33–8.18)
Urban	18.0 (15.2–20.7)	31.9 (31.2–32.6)	11.24 (8.08–14.40)	1.47 (1.31–1.63)	6.29 (4.47–8.12)
Rural	21.0 (20.3–21.7)	29.5 (28.3–30.6)	8.88 (7.02–10.74)	1.42 (1.32–1.52)	5.37 (4.41–6.34)
**Control (national)**	16.0 (15.4–16.7)	19.9 (19.4–20.4)	6.24 (3.39–9.08)	1.39 (1.18–1.59)	3.53 (2.32–4.74)
Urban	14.8 (13.4–16.2)	18.0 (16.3–19.7)	1.56 (−16.60–19.72)	1.09 (0.00–2.18)	4.73 (2.42–7.04)
Rural	19.6 (18.1–21.1)	17.8 (17.1–18.5)	6.80 (−1.05–14.65)	1.47 (0.82–2.12)	1.92 (0.65–3.18)
**Nepal**					
**Prevalence (national)**	26.8 (22.8–30.9)	51.7 (48.1–55.4)	28.92 (21.27–36.57)	2.10 (1.67–2.52)	11.44 (8.48–14.40)
Urban	30.7 (25.4–36.0)	54.1 (49.8–58.4)	28.14 (18.12–38.15)	1.97 (1.48–2.45)	11.631 (7.87–15.39)
Rural	20.3 (14.3–26.4)	46.3 (39.7–52.9)	28.91 (17.27–40.56)	2.32 (1.51–3.12)	12.037 (7.51–16.57)
**Awareness (national)**	9.3 (6.7–12.0)	33.1 (29.7–36.5)	30.24 (23.39–37.08)	4.57 (3.00–6.13)	23.29 (18.41–28.17)
Urban	12.3 (8.5–16.0)	35.4 (31.3–39.5)	30.28 (21.25–39.32)	3.86 (2.28–5.43)	23.40 (16.94–29.85)
Rural	4.5 (1.4–7.5)	27.8 (21.9–33.7)	27.09 (16.97–37.21)	5.79 (2.29–9.28)	21.09 (14.21–27.97)
**Treatment (national)**	4.2 (2.4–6.0)	17.7 (14.9–20.5)	18.32 (12.96–23.69)	5.33 (2.96–7.70)	27.41 (19.89–34.93)
Urban	4.7 (2.2–7.1)	19.5 (16.1–22.9)	20.01 (13.17–26.85)	5.27 (2.45–8.09)	29.86 (19.87–39.85)
Rural	3.4 (0.7–6.1)	13.5 (9.0–18.0)	13.97 (5.51–22.44)	4.81 (1.03–8.60)	19.90 (9.88–29.92)
**Control (national)**	14.3 (14.1–14.5)	9.4 (9.2–9.6)	−6.96 (−7.39– −6.52)	0.57 (0.54–0.59)	−9.26 (−9.78– −8.74)
Urban	17.3 (16.9–17.7)	10.1 (9.9–10.4)	−8.56 (−9.50– −7.62)	0.53 (0.49–0.56)	−10.30 (−11.41– −9.19)
Rural	13.6 (13.4–13.7)	8.3 (8.0–8.6)	−7.73 (− 8.17– − 7.29)	0.51 (0.49–0.53)	− 10.70 (− 11.24– −10.16)

At sub-national levels, India’s North region had highest wealth-based absolute inequality in prevalence of hypertension, where hypertension was more prevalent in richer and richest population than their poorer counterparts. The Northeast and Central part of India had highest absolute inequalities in awareness and treatment. Wealth-based inequality in control of hypertension was highest in the South and Northeast regions. (Fig. 3 and Supplementary Table 8 p18). In Nepal, compared to other provinces, Province 3 had substantially high wealth-based inequalities in prevalence 19.4% (95% CI 9.1–29.9), awareness 43.4% (26.1–60.8), treatment 43.4% (27.7–59.1), and control 29.6% (15.9–43.2) of hypertension followed by Province 6 and 7. Absolute inequalities in prevalence and management of hypertension at sub-national levels were significantly higher in Nepal compared to India. Details are provided in Fig. 3, Supplementary Tables 5–12 p11, and Supplementary Fig. 11,12,15 p45 & 17–24 p51).

### Education-based inequality in prevalence and management of hypertension

Prevalence of hypertension in India and Nepal was higher among the population with no formal education than their higher educated counterparts. However, awareness, treatment, and control were higher in educated populations than the uneducated ones in India. In Nepal, awareness and treatment were concentrated in the uneducated population (Supplementary Tables 13–20 p23 and Supplementary Figs. 13–15 p47 & 25–32 p57).

Figure 1 shows the concentration indices for prevalence, awareness, treatment, and control of hypertension in India and Nepal, decomposed by age group, household wealth quintile, body mass index, and education. In both countries, BMI was the major factor that accounted for the pro-rich concentration in prevalence of hypertension. Education didn’t show notable contribution towards the pro-rich concentration in treatment and control of hypertension in both countries.

## Discussion

This study provides a concrete evidence regarding wealth-and education-based inequalities in prevalence and management of hypertension at national and subnational levelsin young adults aged 15–49 years, in India and Nepal. It shows that wealth-and education-based inequalities in prevalence and management of hypertension especially treatment and control were significantly high and varied among different regions in India and Nepal.

In this study we found around 11% percent of the young adults aged 15–49 years in India, and almost 20% of young adults in Nepal were hypertensive. More than half of the hypertensive population were unaware of their conditions and almost two-thirds did not receive treatment. Above 80% of the hypertensive population had uncontrolled blood pressure in both countries. These findings were consistent with previous studies [7, 30, 31]. The risk factor analysis showed that the odds of being hypertensive in men were significantly higher than women in both countries. In contrary, odds of being treated and having their blood pressure controlled in women were twice as high as compared to men. A plausible reason includes higher health seeking behavior in women [32, 33]. Odds of being hypertensive, were notably higher among urban residents compared to rural residents. This could be because of unplanned urbanization, environmental factors such as air pollution, high disparities in living standards, and other behavioral risk factors [34, 35]. In both counties, adults aged between 35 and 49 years, with higher BMI had approximately four times higher odds of being hypertensive and were two times less likely to have their blood pressure controlled compared to individuals with normal BMI. The reasons could be low physical activity, long work hours, and sedentary lifestyle [36–38].

Wide regional variability existed in prevalence, awareness, treatment, and control of hypertension in both countries. For instance, some regions in India with disproportionately high prevalence such as the Northeast region, had significantly low awareness, treatment, and control. One of the probable reasons for high prevalence in this region could be unhealthy lifestyles and dietary pattern [38]. Further, low awareness, treatment, and control of hypertension could be due to inadequate knowledge among people about the risk factors of NCDs [39, 40], unaffordable anti-hypertensive medication or lack of treatment services, and distant heath care centers [41]. It was important to note that in both countries, the regions with low prevalence of hypertension, also had low proportion of awareness, treatment, and control which indicates high burden in these regions. Thus, it signifies along with prevalence estimate, estimates for awareness, treatment, and control are important to understand the overall burden of hypertension and ensure effective policy formulation and implementation.

Wealth-and education-based inequalities in awareness, treatment, and control in India and Nepal were remarkably high across all the regions and were highly concentrated in the affluent population. Importantly, wealth-based inequalities in treatment and control were three times higher in Nepal compared to India. This could be due to high disparity in income distribution within different provinces of Nepal [16, 42]. In both countries, some regions for example, East region in India and Province 3 in Nepal had higher levels of awareness, treatment, and control demonstrating better scenarios in hypertension management. However, wealth-and education-based inequality estimates showed that the awareness, treatment, and control of hypertension varied among the subgroups with high concentration towards wealthy and educated population. These results imply the poorest and disadvantaged population were still suffering from high burden of hypertension. Thus, inequality estimates at sub-national levels provided important insight to understand the actual burden of hypertension in different regions and subgroups.

High disparity in prevalence, awareness, treatment, and control of hypertension among different regions entails the importance of thorough intervention at regional levels to tackle the existing problem. Thorough implementation of prevention and treatment strategies recommended by World Health Organization (WHO), the International Society of Hypertension (ISH) and Centers for Disease Control and Prevention (CDC) could be helpful to manage the burden of hypertension [43]. Building better healthcare infrastructure, improving standardized treatment services, and using modern technology such as mobile health applications could help to increase awareness, treatment, and control of hypertension [44, 45].

At present, the governments in both countries are struggling to mitigate the challenges of communicable diseases [46, 47]. The increased risk of hypertension creates a havoc for policy makers and governmental bodies to manage the double burden of diseases. Hence, prioritizing prevention and management of hypertension as a national agenda and thoroughly monitoring the progress at regional level would be urgently needed.

There are several studies conducted to estimate burden of hypertension in low-and middle-income countries including South Asian countries. These studies have shown that the burden of hypertension in low-and middle-income countries is high, particularly in south Asian countries [2, 48]. Most of these studies suggest that hypertension is mostly prevalent in wealthy people. Some studies suggest that in the south Asian countries, burden of hypertension particularly in India and Nepal are in increasing trend with increasing urbanization and sedentary lifestyle [21, 31]. Several studies have estimated the prevalence of hypertension and its management in both countries [7, 31], but none of these studies performed inequality assessment at national and sub-national levels. Therefore, this study provides detailed information on existing inequalities in prevalence and management of hypertension in these two neighboring countries that share similar lifestyle, culture, and religions, yet with differences in demographic and socio-economic characteristics. This study has a few limitations. Firstly, we excluded population aged 49 years and above due to missing data. Hence, this study may not be generalized to population aged 50 years and above. However, this study emphasizes the increasing burden of hypertension in younger adults which is a major issue in many high-income countries [49, 50]. Secondly, this is a cross-sectional study therefore a causal relationship cannot be necessarily established between the covariates and outcomes. Lastly, there could be possibilities of misreporting because of single-day measurements. However, SBP and DBP were measured three times to minimize the possibility of misreporting.

## Conclusion

Our study showed that India and Nepal had high inequalities in prevalence and management of hypertension at national and sub-national levels. There was wide gap in awareness, treatment, and control of hypertension among different subgroups within a region. Hence, for effective management of hypertension, tailored strategies are required for specific regions by considering several socio-economic and demographic factors such as socio-economic status, education level, and BMI. More efforts should be put towards awareness campaigns taking SES inequality into consideration. Community-based behavioral interventions such as change in dietary pattern, increase in physical activity, and routine health checkups should be encouraged to manage hypertension in both countries. Effective screening and treatment services should be made easily available and affordable for everyone regardless of their SES. In addition, involvement of the private sector should be encouraged for the sustainable management of hypertension. Further studies are needed to explore the inequality issues and its major factors and the association of hypertension with other comorbidities such as diabetes in India and Nepal.

## Supplementary information

**Additional file 1.**

## Data Availability

The datasets analyzed during the study are available in the Demographic and Health Surveys, DHS repository, https://dhsprogram.com/Data/

## Abbreviations

BP: Blood Pressure
BMI: Body Mass Index
CBS: Central Bureau of Statistics
CC: Concentration Curve
CDC: Centers for Disease Control and Prevention
CEBs: Census Enumeration Blocks
CI: Confidence Interval
CIX: Concentration Index
CVDs: Cardiovascular Diseases
DALYs: Disabilities Adjusted Life Years
DBP: Diastolic Blood Pressure
GDP: Gross Domestic Product
EAs: Enumeration Areas
HICs: High Income Countries
ISH: International Society of Hypertension
LIC: Low Income Countries
LMICs: Low-and Middle-Income Countries
MoHFW: Ministry of Health and Family Welfare
MLM: Multi-level model
NCDs: Non–communicable Diseases
NDHS: Nepal Demographic and Health Surveys
NFHS: National Family Health Survey
NPHC: National Population and Housing Census
OOP: Out-of-Pocket
OR: Odds Ratio
PSU: Primary Sampling Unit
RII: Relative Index of Inequality
SBP: Systolic Blood Pressure
SDG: Sustainable Development Goals
SES: Socio-Economic Status
SII: Slope Index of Inequality
UHC: Universal Health Coverage
UN: United Nations
WB: World Bank
WHO: World Health Organization
