# Supplementary material for: Inequalities and risk factors analysis in prevalence and management of hypertension in India and Nepal: a national and subnational study
Source: BMC Public Health. 2020 Sep 3;20:1341. doi: 10.1186/s12889-020-09450-6 (PMC7469349; doi:10.1186/s12889-020-09450-6)
Supplement: Supplementary file 1 — Additional file 1. [file 12889_2020_9450_MOESM1_ESM.docx]

**Supplementary Tables**

1. Supplementary Table 1. Prevalence, awareness, treatment and control of hypertension in India, 2016

2. Supplementary Table 2. Prevalence, awareness, treatment and control of hypertension in Nepal,2016

3. Supplementary Table 3. Risk factor of prevalence, awareness, treatment and control of hypertension in India, 2016

4. Supplementary Table 4. Risk factor of prevalence, awareness, treatment and control of hypertension in Nepal, 2016

5. Supplementary Table 5. Wealth–based inequality in prevalence of hypertension, India, 2016

6. Supplementary Table 6. Wealth–based inequality in awareness of hypertension, India, 2016

7. Supplementary Table 7. Wealth–based inequality in treatment of hypertension, India, 2016

8. Supplementary Table 8. Wealth–based inequality in control of hypertension, India, 2016

9. Supplementary Table 9. Wealth–based inequality in prevalence of hypertension, Nepal, 2016

10. Supplementary Table 10. Wealth–based inequality in awareness of hypertension, Nepal, 2016

11. Supplementary Table 11. Wealth–based inequality in treatment of hypertension, Nepal, 2016

12. Supplementary Table 12. Wealth–based inequality in control of hypertension, Nepal, 2016

13. Supplementary Table 13. Education–based inequality in prevalence of hypertension, India, 2016

14. Supplementary Table 14. Education–based inequality in awareness of hypertension, India, 2016

15. Supplementary Table 15. Education–based inequality in treatment of hypertension, India, 2016

16. Supplementary Table 16. Education–based inequality in control of hypertension, India, 2016

17. Supplementary Table 17. Education–based inequality in prevalence of hypertension, Nepal, 2016

18. Supplementary Table 18. Education–based inequality in awareness of hypertension, Nepal, 2016

19. Supplementary Table 19. Education–based inequality in treatment of hypertension, Nepal, 2016

20. Supplementary Table 20. Education–based inequality in control of hypertension, Nepal, 2016

**Supplementary Figures**

1. Supplementary Figure 1. Awareness control of hypertension by household wealth quintile and education in India and Nepal, 2016

2. Supplementary Figure 2. Prevalence, awareness, treatment and control of hypertension based on socio–economic status and body mass index in India and Nepal, 2016

3. Supplementary Figure 3. Prevalence, awareness, treatment and control of hypertension based on education-levels and body mass index in India and Nepal, 2016

4. Supplementary Figure 4. Awareness and control of hypertension at subnational levels in India and Nepal, 2016

5. Supplementary Figure 5. Prevalence and management of hypertension in urban residence in India and Nepal, 2016

6. Supplementary Figure 6. Prevalence and management of hypertension in rural residence in India and Nepal, 2016

7. Supplementary Figure 7. Prevalence and management of hypertension in females in India and Nepal, 2016

8. Supplementary Figure 8. Prevalence and management of hypertension in males in India and Nepal, 2016

9. Supplementary Figure 9. Prevalence and management of hypertension at regional level by urban and rural residence in India, 2016

10. Supplementary Figure 10. Prevalence and management of hypertension at regional level by gender in India, 2016

11. Supplementary Figure 11. Wealth-based SII in prevalence and awareness of hypertension in India and Nepal

12. Supplementary Figure 12. Wealth-based relative index of inequalities in prevalence and management of hypertension at subnational level in India and Nepal, 2016

13. Supplementary Figure 13. Education-based slope index of inequalities in prevalence and management of hypertension at subnational level in India and Nepal, 2016

14. Supplementary Figure 14. Education-based relative index of inequalities in prevalence and management of hypertension at subnational level in India and Nepal, 2016

15. Supplementary Figure 15. Slope index of inequalities in prevalence and management of hypertension by wealth quintile and education at regional level in India, 2016

16. Supplementary Figure 16. Decomposition of concentration index, India and Nepal 2016

17. Supplementary Figure 17. Equiplots for prevalence of hypertension by household wealth quintile in India, 2016

18. Supplementary Figure 18. Equiplots for awareness, of hypertension by household wealth quintile in India, 2016.

19. Supplementary Figure 19. Equiplots for treatment of hypertension by household wealth quintile in India, 2016.

20. Supplementary Figure 20. Equiplots for control of hypertension by household wealth quintile in India, 2016.

21. Supplementary Figure 21. Equiplots for prevalence of hypertension by household wealth quintile in Nepal, 2016.

22. Supplementary Figure 22. Equiplots for awareness of hypertension by household wealth quintile in Nepal, 2016

23. Supplementary Figure 23. Equiplots for treatment of hypertension by household wealth quintile in Nepal, 2016.

24. Supplementary Figure 24. Equiplots for control of hypertension by household wealth quintile in Nepal, 2016.

25. Supplementary Figure 25. Equiplots for prevalence of hypertension by level of education in India, 2016.

26. Supplementary Figure 26. Equiplots for awareness of hypertension by level of education in India, 2016.

27. Supplementary Figure 27. Equiplots for treatment of hypertension by level of education in India, 2016.

28. Supplementary Figure 28. Equiplots for control of hypertension by level of education in India, 2016.

29. Supplementary Figure 29. Equiplots for prevalence of hypertension by level of education in Nepal, 2016

30. Supplementary Figure 30. Equiplots for awareness of hypertension by level of education in Nepal, 2016.

31. Supplementary Figure 31. Equiplots for treatment of hypertension by level of education in Nepal, 2016

32. Supplementary Figure 32. Equiplots for control of hypertension by level of education in Nepal, 2016.

**Supplementary Methods**

1. Supplementary Method 1. Data Collection

2. Supplementary Method 2. Sampling weight

3. Supplementary Method 3. Independent variables

4. Supplementary Method 4. Statistical Analysis

**Supplementary Table 1. Prevalence, awareness, treatment and control of hypertension in India, 2016**

| **Characteristics** | **Proportion (95% confidence interval)** | | | |
| --- | --- | --- | --- | --- |
|  | **Hypertension (n=781,841)** | **Awareness (n=89,481)** | **Treatment (n=89,481)** | **Control (n=89,481)** |
| **National** | 11.4 (11.4 - 11.5) | 30.3 (30.0 - 30.6) | 26.4 (26.2 - 26.7) | 17.8 (17.5 - 18.0) |
| **Age group(years)** |  |  |  |  |
| 15-25 | 3.8 (3.7-3.9) | 21.4 (20.7-22.2) | 31.4 (30.5-32.3) | 29.3 (28.4-30.2) |
| 25-35 | 9.3 (9.1-9.4) | 25.8 (25.2-26.3) | 23.9 (23.4-24.5) | 19.3 (18.8-19.8) |
| 35-45 | 18.5 (18.3-18.7) | 31.2 (30.7-31.7) | 24.4 (24.0-24.9) | 14.6 (14.2-14.9) |
| 45-49 | 26.5 (26.2-26.8) | 37.6 (37.0-38.2) | 29.8 (29.2-30.5) | 16.0 (15.5-16.5) |
| **Sex** |  |  |  |  |
| Men | 14.8 (14.6-15.0) | 19.2 (18.6-19.8) | 14.6 (14.0-15.2) | 8.7 (8.3-9.2) |
| Women | 11.0 (10.9-11.0) | 32.5 (32.2-32.8) | 28.8 (28.4-29.1) | 19.6 (19.3-19.8) |
| **Educational status** |  |  |  |  |
| No education | 14.3 (14.2-14.5) | 27.7 (27.2-28.2) | 24.0 (23.5-24.5) | 15.5 (15.1-15.9) |
| Primary education | 13.5 (13.3-13.7) | 29.9 (29.2-30.7) | 25.6 (24.9-26.3) | 15.9 (15.3-16.6) |
| Secondary education | 10.0 (9.9-10.1) | 31.6 (31.1-32.0) | 27.7 (27.3-28.2) | 19.0 (18.6-19.4) |
| Higher education | 9.4 (9.2-9.6) | 33.4 (32.5-34.3) | 29.7 (28.8-30.6) | 22.1 (21.2-22.9) |
| **Marital status** |  |  |  |  |
| Never married | 4.4 (4.3-4.5) | 17.6 (16.8-18.5) | 24.9 (24.0-25.8) | 22.5 (21.6-23.4) |
| Married | 13.5 (13.4-13.6) | 31.6 (31.2-31.9) | 26.6 (26.3-26.9) | 17.4 (17.1-17.7) |
| Widowed | 20.3 (19.8-20.8) | 34.2 (32.8-35.6) | 27.7 (26.4-29.1) | 15.8 (14.8-17.0) |
| Divorced | 14.5 (13.2-16.0) | 30.1 (25.6-35.1) | 20.9 (17.0-25.4) | 12.2 (9.2-16.1) |
| Not Living together | 13.3 (12.4-14.2) | 26.0 (22.9-29.3) | 22.7 (19.7-25.9) | 16.5 (13.9-19.4) |
| **Tobacco consumption** |  |  |  |  |
| No | 10.9 (10.8-10.9) | 31.7 (31.4-32.0) | 28.0 (27.7-28.3) | 19.1 (18.8-19.4) |
| Yes | 15.8 (15.6-16.1) | 23.2 (22.5-23.9) | 18.3 (17.7-19.0) | 10.9 (10.4-11.4) |
| **Alcohol consumption** |  |  |  |  |
| No | 11.1 (11.0-11.1) | 31.2 (30.9-31.5) | 27.5 (27.2-27.9) | 18.7 (18.5-19.0) |
| Yes | 19.0 (18.6-19.4) | 20.3 (19.4-21.3) | 13.8 (13.0-14.6) | 6.8 (6.2-7.4) |
| **Body mass index** |  |  |  |  |
| Underweight | 5.6 (5.5-5.7) | 23.3 (22.5-24.2) | 28.1 (27.2-29.0) | 24.2 (23.4-25.1) |
| Normal | 9.6 (9.5-9.7) | 26.0 (25.6-26.4) | 24.4 (24.0-24.9) | 17.9 (17.6-18.3) |
| Overweight | 20.9 (20.6-21.1) | 34.3 (33.7-34.8) | 25.5 (24.9-26.0) | 14.6 (14.1-15.0) |
| Obese | 29.6 (29.1-30.0) | 44.3 (43.3-45.2) | 35.0 (34.1-35.9) | 18.9 (18.2-19.6) |
| **Wealth quintile** |  |  |  |  |
| Q1 (Poorest) | 9.4 (9.3-9.6) | 19.2 (18.5-19.9) | 20.8 (20.1-21.5) | 16.1 (15.4-16.7) |
| Q2 | 10.1 (9.9-10.2) | 26.2 (25.5-26.9) | 24.2 (23.5-24.8) | 17.1 (16.5-17.7) |
| Q3 | 11.0 (10.8-11.1) | 29.7 (29.0-30.4) | 25.4 (24.7-26.0) | 17.2 (16.7-17.8) |
| Q4 | 12.9 (12.8-13.1) | 33.0 (32.4-33.7) | 27.5 (26.9-28.1) | 17.6 (17.1-18.1) |
| Q5 (Richest) | 13.4 (13.2-13.5) | 37.7 (37.0-38.3) | 31.2 (30.6-31.9) | 19.9 (19.4-20.5) |
| **Availability of salt** |  |  |  |  |
| Salt available | 11.5 (11.4-11.6) | 30.5 (30.2-30.8) | 26.5 (26.2-26.8) | 17.7 (17.4-17.9) |
| No salt in house | 11.0 (9.6-12.6) | 22.4 (17.0-28.9) | 20.2 (15.1-26.6) | 14.6 (10.3-20.4) |
| **Place of residence** |  |  |  |  |
| Urban | 12.7 (12.5-12.8) | 34.8 (34.3-35.3) | 29.0 (28.5-29.4) | 18.5 (18.1-19.0) |
| Rural | 10.8 (10.7-10.9) | 27.6 (27.2-28.0) | 24.9 (24.5-25.3) | 17.3 (17.0-17.6) |
| **Region** |  |  |  |  |
| North | 12.1 (11.9-12.3) | 35.9 (35.1-36.7) | 28.5 (27.7-29.3) | 18.5 (17.9-19.2) |
| Central | 9.7 (9.5-9.8) | 24.0 (23.4-24.7) | 21.7 (21.1-22.3) | 15.8 (15.2-16.3) |
| East | 10.5 (10.4-10.7) | 30.8 (30.1-31.5) | 27.7 (27.1-28.4) | 18.5 (18.0-19.1) |
| Northeast | 17.4 (17.0-17.9) | 28.7 (27.4-30.0) | 21.8 (20.7-23.0) | 11.2 (10.3-12.1) |
| West | 12.2 (12.0-12.4) | 23.9 (23.2-24.6) | 24.3 (23.6-25.0) | 17.2 (16.6-17.8) |
| South | 12.4 (12.2-12.5) | 36.3 (35.7-37.0) | 30.5 (29.9-31.1) | 20.1 (19.6-20.7) |
| **State** |  |  |  |  |
| Andaman and Nicobar | 13.7 (10.0-18.4) | 38.1 (23.7-55.0) | 36.7 (22.6-53.7) | 17.5 (8.1-33.7) |
| Andre Pradesh | 13.2 (12.8-13.5) | 31.5 (30.1-32.9) | 26.3 (25.0-27.6) | 17.1 (16.0-18.3) |
| Arunachal Pradesh | 17.4 (14.7-20.5) | 26.9 (19.6-35.8) | 18.2 (12.1-26.3) | 9.6 (5.4-16.5) |
| Assam | 18.3 (17.8-18.9) | 26.7 (25.2-28.1) | 20.6 (19.3-21.9) | 9.7 (8.7-10.7) |
| Bihar | 8.5 (8.3-8.8) | 30.7 (29.4-31.9) | 33.9 (32.6-35.2) | 26.7 (25.5-27.9) |
| Chandigarh | 11.5 (9.2-14.3) | 40.1 (29.1-52.1) | 26.5 (17.4-38.2) | 14.2 (7.7-24.7) |
| Chhattisgarh | 10.3 (9.9-10.8) | 16.7 (15.1-18.4) | 15.7 (14.1-17.4) | 10.4 (9.1-11.8) |
| Dadra and Nagar Haveli | 10.1 (6.7-15.0) | 26.9 (12.2-49.4) | 26.7 (12.0-49.2) | 17.1 (6.1-39.5) |
| Daman and Diu | 10.5 (6.2-17.4) | 28.6 (10.1-58.7) | 36.1 (14.7-65.0) | 29.4 (10.6-59.3) |
| Goa | 12.7 (11.0-14.7) | 26.4 (20.1-33.8) | 31.1 (24.4-38.8) | 20.5 (14.9-27.6) |
| Gujarat | 12.0 (11.7-12.3) | 20.5 (19.3-21.6) | 20.8 (19.7-22.0) | 14.4 (13.5-15.5) |
| Haryana | 14.1 (13.6-14.6) | 46.1 (44.1-48.0) | 37.0 (35.1-38.9) | 27.0 (25.3-28.8) |
| Himachal Pradesh | 16.0 (15.0-17.1) | 30.3 (27.1-33.8) | 22.9 (20.0-26.1) | 13.6 (11.3-16.3) |
| Jammu and Kashmir | 16.0 (15.2-16.8) | 45.0 (42.4-47.7) | 38.7 (36.1-41.3) | 25.2 (22.9-27.6) |
| Jharkhand | 9.5 (9.1-9.9) | 20.0 (18.2-21.8) | 17.4 (15.7-19.2) | 13.0 (11.5-14.6) |
| Karnataka | 12.2 (11.9-12.5) | 23.3 (22.2-24.6) | 22.6 (21.4-23.8) | 14.3 (13.3-15.3) |
| Kerala | 9.5 (9.2-9.9) | 38.2 (36.1-40.2) | 33.0 (31.1-35.1) | 24.1 (22.3-25.9) |
| Lakshadweep | 13.8 (6.6-26.7) | 31.0 (7.2-72.4) | 23.5 (4.3-67.7) | 13.7 (1.5-62.9) |
| Madhya Pradesh | 10.1 (9.8-10.3) | 23.8 (22.6-25.0) | 24.7 (23.5-25.9) | 17.3 (16.3-18.4) |
| Maharashtra | 12.3 (12.1-12.6) | 25.6 (24.7-26.5) | 25.9 (25.0-26.8) | 18.6 (17.8-19.4) |
| Manipur | 13.6 (11.9-15.5) | 29.1 (23.0-36.0) | 15.3 (10.8-21.3) | 7.8 (4.7-12.7) |
| Meghalaya | 14.0 (12.4-15.7) | 42.1 (36.0-48.4) | 39.6 (33.6-45.9) | 28.6 (23.2-34.6) |
| Mizoram | 12.8 (10.5-15.7) | 37.8 (27.9-48.7) | 26.7 (18.2-37.3) | 16.9 (10.2-26.7) |
| Nagaland | 17.5 (15.1-20.2) | 29.6 (22.9-37.4) | 12.4 (8.0-18.7) | 4.9 (2.4-9.8) |
| Delhi | 8.6 (8.1-9.2) | 37.1 (33.9-40.5) | 34.1 (30.9-37.4) | 22.7 (20.0-25.6) |
| Odisha | 12.1 (11.8-12.5) | 36.6 (34.9-38.2) | 31.9 (30.4-33.5) | 23.3 (21.9-24.8) |
| Puducherry | 12.8 (10.8-15.2) | 53.6 (44.5-62.4) | 36.7 (28.5-45.8) | 22.2 (15.6-30.7) |
| Punjab | 16.1 (15.5-16.6) | 37.7 (35.9-39.5) | 22.4 (20.9-24.0) | 10.5 (9.4-11.7) |
| Rajasthan | 9.1 (8.9-9.4) | 26.5 (25.1-27.9) | 24.1 (22.8-25.5) | 17.0 (15.9-18.3) |
| Sikkim | 19.9 (16.1-24.4) | 42.1 (31.2-53.8) | 24.2 (15.6-35.4) | 9.9 (4.8-19.3) |
| Tamil Nadu | 12.4 (12.1-12.7) | 45.1 (44.0-46.3) | 36.5 (35.4-37.6) | 24.3 (23.4-25.3) |
| Tripura | 15.7 (14.3-17.2) | 34.2 (29.7-39.1) | 28.2 (24.0-32.9) | 17.7 (14.2-21.8) |
| Uttar Pradesh | 9.4 (9.2-9.6) | 25.4 (24.6-26.2) | 21.4 (20.7-22.2) | 16.0 (15.3-16.7) |
| Uttarakhand | 12.7 (11.9-13.6) | 31.0 (28.0-34.3) | 26.8 (23.9-29.9) | 17.2 (14.8-19.9) |
| West Bengal | 12.1 (11.9-12.4) | 31.0 (30.0-32.1) | 24.0 (23.1-25.0) | 12.0 (11.3-12.8) |
| Telangana | 14.0 (13.6-14.5) | 39.7 (38.1-41.4) | 31.9 (30.3-33.5) | 20.5 (19.2-21.9) |

**Supplementary Table 2. Prevalence, awareness, treatment and control of hypertension in Nepal,2016**

| **Characteristics** | **Proportion (95% confidence interval)** | | | |
| --- | --- | --- | --- | --- |
|  | **Hypertension (n= 14,494)** | **Awareness (n=2,836)** | **Treatment (n=2,836)** | **Control (n=2,836)** |
| **National** | 19.6 (18.9 - 20.2) | 40.0 (38.2 - 41.8) | 20.2 (18.8 - 21.7) | 10.4 (9.3 - 11.6) |
| **Age group (years)** |  |  |  |  |
| 15-25 | 3.8 (3.2-4.4) | 13.9 (9.3-20.5) | 6.5 (3.5-11.8) | 5.9 (3.1-11.0) |
| 25-35 | 11.5 (10.4-12.7) | 26.9 (22.5-31.8) | 7.4 (5.0-10.6) | 5.0 (3.1-7.8) |
| 35-45 | 22.5 (20.9-24.2) | 36.1 (32.2-40.2) | 13.8 (11.2-16.9) | 7.1 (5.2-9.5) |
| 45-59 | 31.0 (28.1-33.9) | 36.9 (31.5-42.6) | 17.2 (13.3-22.0) | 9.2 (6.4-13.1) |
| **Sex** |  |  |  |  |
| Men | 23.4 (22.4-24.5) | 36.9 (34.4-39.4) | 18.0 (16.1-20.1) | 9.6 (8.2-11.2) |
| Women | 16.8 (16.0-17.6) | 43.2 (40.6-45.8) | 22.4 (20.3-24.6) | 11.2 (9.7-13.0) |
| **Educational status** |  |  |  |  |
| No education | 24.6 (23.5-25.8) | 41.9 (39.2-44.5) | 21.3 (19.2-23.5) | 10.7 (9.1-12.4) |
| Primary education | 20.8 (19.2-22.5) | 38.4 (34.3-42.8) | 18.2 (15.1-21.9) | 8.8 (6.6-11.6) |
| Secondary education | 14.7 (13.7-15.7) | 37.5 (33.9-41.2) | 19.1 (16.4-22.3) | 10.0 (7.9-12.4) |
| Higher education | 15.6 (14.0-17.2) | 40.5 (35.1-46.1) | 21.2 (17.0-26.1) | 12.8 (9.5-17.0) |
| **Marital status** |  |  |  |  |
| Never married | 4.9 (4.1-5.7) | 15.4 (10.2-22.6) | 7.7 (4.2-13.7) | 6.2 (3.1-11.8) |
| Married | 21.4 (20.7-22.2) | 40.5 (38.5-42.5) | 19.5 (17.9-21.2) | 10.2 (9.1-11.6) |
| Widowed | 38.5 (35.6-41.5) | 47.1 (42.2-52.0) | 28.9 (24.7-33.6) | 13.1 (10.2-16.9) |
| Divorced | 23.2 (16.6-31.3) | 20.9 (9.7-39.5) | 16.3 (6.7-34.5) | 5.3 (1.1-22.3) |
| **Tobacco consumption** |  |  |  |  |
| No | 18.6 (17.9-19.2) | 41.2 (39.3-43.2) | 21.4 (19.8-23.0) | 11.2 (10.0-12.5) |
| Yes | 28.6 (26.3-31.0) | 33.0 (28.7-37.7) | 13.5 (10.5-17.1) | 5.7 (3.9-8.4) |
| **Alcohol consumption** |  |  |  |  |
| No | 19.4 (18.8-20.1) | 40.2 (38.4-42.0) | 20.5 (19.0-22.0) | 10.6 (9.5-11.8) |
| Yes | 31.8 (25.8-38.5) | 33.4 (23.1-45.7) | 8.2 (3.6-17.9) | 1.1 (0.1-10.3) |
| **Body mass index** |  |  |  |  |
| Underweight | 11.9 (10.7-13.1) | 30.6 (25.8-35.9) | 13.9 (10.5-18.2) | 7.7 (5.2-11.3) |
| Normal | 16.6 (15.8-17.4) | 36.1 (33.6-38.5) | 18.0 (16.1-20.1) | 10.1 (8.7-11.8) |
| Overweight | 33.8 (31.9-35.8) | 47.4 (43.9-50.9) | 23.9 (21.0-27.0) | 10.5 (8.5-12.8) |
| Obese | 42.1 (38.1-46.3) | 53.2 (46.7-59.6) | 28.3 (22.8-34.5) | 14.9 (10.9-20.1) |
| **Wealth Quintile** |  |  |  |  |
| Q1 (Poorest) | 18.1 (16.6-19.6) | 26.8 (23.0-31.1) | 9.3 (7.0-12.4) | 4.2 (2.7-6.5) |
| Q2 | 17.4 (16.1-18.8) | 33.9 (29.9-38.1) | 12.6 (10.0-15.7) | 5.1 (3.5-7.4) |
| Q3 | 18.8 (17.5-20.2) | 38.7 (34.9-42.7) | 17.2 (14.3-20.4) | 8.9 (6.9-11.5) |
| Q4 | 18.7 (17.3-20.2) | 42.6 (38.5-46.9) | 22.5 (19.2-26.3) | 12.5 (9.9-15.6) |
| Q5 (Richest) | 24.5 (23.0-26.1) | 51.8 (48.1-55.3) | 33.1 (29.8-36.6) | 17.7 (15.1-20.6) |
| **Food Security** |  |  |  |  |
| Food secure | 22.1 (21.1-23.1) | 44.8 (42.3-47.3) | 23.7 (21.7-25.9) | 13.2 (11.6-15.0) |
| Mildly insecure | 17.9 (16.7-19.3) | 34.4 (30.7-38.3) | 16.4 (13.6-19.5) | 7.8 (5.9-10.3) |
| Moderately insecure | 17.2 (15.9-18.5) | 34.6 (30.7-38.8) | 16.8 (13.8-20.2) | 6.4 (4.6-8.8) |
| Severely insecure | 15.9 (13.9-18.0) | 35.3 (28.8-42.3) | 13.8 (9.6-19.5) | 7.7 (4.7-12.5) |
| **Availability of salt** |  |  |  |  |
| Salt available | 15.4 (12.9-18.3) | 23.8 (16.6-32.9) | 9.6 (5.3-17.0) | 4.3 (1.7-10.4) |
| No salt in house | 13.3 (7.8-21.9) | 44.7 (20.2-72.1) | NA | NA |
| **Place of residence** |  |  |  |  |
| Urban | 20.6 (19.7-21.4) | 42.7 (40.4-45.0) | 22.8 (21.0-24.8) | 11.6 (10.2-13.2) |
| Rural | 18.0 (17.0-19.0) | 35.2 (32.3-38.2) | 15.5 (13.4-17.8) | 8.2 (6.7-10.1) |
| **Geographical region** |  |  |  |  |
| Himal | 17.2 (14.9-19.8) | 35.9 (28.7-43.8) | 12.4 (8.1-18.6) | 6.4 (3.4-11.5) |
| Pahaad | 22.5 (21.5-23.5) | 38.9 (36.4-41.5) | 18.6 (16.7-20.7) | 9.9 (8.5-11.6) |
| Terai | 17.3 (16.5-18.2) | 41.8 (39.1-44.5) | 23.0 (20.7-25.4) | 11.5 (9.8-13.4) |
| **Region of residence** |  |  |  |  |
| Province 1 | 19.0 (17.5-20.6) | 44.1 (39.7-48.5) | 23.0 (19.5-27.0) | 12.8 (10.1-16.1) |
| Province 2 | 15.0 (13.8-16.3) | 43.4 (38.8-48.0) | 24.5 (20.7-28.7) | 13.3 (10.4-16.7) |
| Province 3 | 23.2 (21.8-24.8) | 42.6 (39.1-46.2) | 23.4 (20.5-26.6) | 13.6 (11.3-16.3) |
| Province 4 | 26.7 (24.5-29.0) | 40.6 (35.9-45.5) | 18.3 (14.8-22.4) | 7.8 (5.6-10.9) |
| Province 5 | 21.3 (19.7-23.0) | 35.2 (31.2-39.5) | 15.5 (12.6-18.9) | 4.4 (2.9-6.6) |
| Province 6 | 14.8 (12.4-17.5) | 25.7 (18.3-34.8) | 7.3 (3.7-14.0) | 5.4 (2.4-11.7) |
| Province 7 | 13.3 (11.5-15.3) | 30.0 (23.4-37.6) | 13.7 (9.2-20.0) | 9.5 (5.8-15.1) |

**Supplementary Table 3. Risk factor of prevalence, awareness, treatment and control of hypertension in India, 2016**

| **Characteristics** | **Adjusted odds ratio (95% confidence interval), n=781,841** | | | |
| --- | --- | --- | --- | --- |
|  | **Hypertension** | **Awareness** | **Treatment** | **Control** |
| **Age group (years)** |  |  |  |  |
| 15-24 | 1.00 | 1.00 | 1.00 | 1.00 |
| 25-34 | 1.96 (1.87–2.05) *** | 1.06 (0.94–1.19) | 0.59 (0.53–0.66) *** | 0.53 (0.47–0.60) *** |
| 35-44 | 4.02 (3.83–4.21) *** | 1.43 (1.27–1.61) *** | 0.60 (0.54–0.67) *** | 0.36 (0.32–0.41) *** |
| 45-49 | 6.40 (6.08–6.74) *** | 2.05 (1.81–2.32) *** | 0.85 (0.75–0.96) * | 0.41 (0.36–0.47) *** |
| **Sex** |  |  |  |  |
| Men | 1.00 | 1.00 | 1.00 | 1.00 |
| Women | 0.68 (0.66–0.71) *** | 2.29 (2.06–2.55) *** | 2.41 (2.16–2.70) *** | 2.61 (2.28–3.00) *** |
| **Educational status** |  |  |  |  |
| No formal education | 1.00 |  |  |  |
| Primary education | 0.99 (0.96–1.03) | 1.18 (1.09–1.29) *** | 1.15 (1.06–1.26) ** | 1.12 (1.01–1.24) * |
| Secondary education | 0.90 (0.87–0.93) *** | 1.31 (1.21–1.41) *** | 1.22 (1.13–1.32) *** | 1.26 (1.14–1.38) *** |
| Higher education | 0.82 (0.78–0.87) *** | 1.41 (1.25–1.60) *** | 1.32 (1.17–1.49) *** | 1.51 (1.32–1.74) *** |
| **Marital status** |  |  |  |  |
| Never married | 1.00 | 1.00 | 1.00 | 1.00 |
| Married | 1.14 (1.09–1.20) *** | 1.79 (1.58–2.04) *** | 1.33 (1.18–1.50) *** | 1.22 (1.07–1.40) *** |
| Widowed | 1.29 (1.20–1.39) *** | 1.86 (1.56–2.22) *** | 1.34 (1.12–1.61) * | 1.19 (0.97–1.47) |
| Divorced | 1.29 (1.07–1.55) ** | 1.91 (1.18–3.10) * | 1.12 (0.68–1.84) | 1.01 (0.59–1.72) |
| Not Living together | 1.09 (0.94–1.26) | 1.42 (0.98–2.06) * | 1.04 (0.70–1.54) | 1.23 (0.77–1.94) |
| **Tobacco consumption** |  |  |  |  |
| No | 1.00 | 1.00 | 1.00 | 1.00 |
| Yes | 1.01 (0.97–1.04) | 1.02 (0.93–1.11) | 0.97 (0.89–1.06) | 0.93 (0.84–1.04) |
| **Alcohol consumption** |  |  |  |  |
| No | 1.00 | 1.00 | 1.00 | 1.00 |
| Yes | 1.32 (1.25–1.39) *** | 0.83 (0.72–0.95) * | 0.70 (0.61–0.81) *** | 0.54 (0.46–0.65) *** |
| **Body mass index** |  |  |  |  |
| Normal weight | 1.00 | 1.00 | 1.00 | 1.00 |
| Underweight | 0.68 (0.66–0.71) *** | 1.06 (0.97–1.16) | 1.32 (1.21–1.44) *** | 1.49 (1.36–1.63) *** |
| Overweight | 2.04 (1.98–2.10) *** | 1.30 (1.21–1.39) *** | 0.93 (0.87–1.00) | 0.69 (0.63–0.75) *** |
| Obese | 3.29 (3.15–3.44) *** | 2.00 (1.81–2.20) *** | 1.49 (1.35–1.64) *** | 0.95 (0.84–1.07) |
| **Wealth quintile** |  |  |  |  |
| Q1 (Poorest) | 1.00 | 1.00 | 1.00 | 1.00 |
| Q2 | 1.03 (0.99–1.07) | 1.24 (1.13–1.36) *** | 1.12 (1.02–1.24) * | 1.01 (0.91–1.12) |
| Q3 | 1.04 (1.00–1.08) * | 1.35 (1.23–1.50) *** | 1.17 (1.06–1.29) * | 1.07 (0.96–1.20) |
| Q4 | 1.10 (1.06–1.15) *** | 1.51 (1.36–1.67) *** | 1.25 (1.12–1.38) *** | 1.04 (0.93–1.18) |
| Q5 (Richest) | 1.11 (1.06–1.16) *** | 1.82 (1.63–2.05) *** | 1.58 (1.41–1.77) *** | 1.30 (1.14–1.48) *** |
| **Place of residence** |  |  |  |  |
| Rural | 1.00 | 1.00 | 1.00 | 1.00 |
| Urban | 0.97 (0.94–1.00) | 1.13 (1.06–1.21) *** | 1.09 (1.02–1.16) * | 1.07 (0.99–1.16) |

*** p-value < 0.001 ** p-value < 0.01 * p-value < 0.05

**Supplementary Table 4. Risk factor of prevalence, awareness, treatment and control of hypertension in Nepal, 2016**

| **Characteristics** | **Adjusted odds ratio (95% confidence interval), n=14,494** | | | |
| --- | --- | --- | --- | --- |
|  | **Hypertension** | **Awareness** | **Treatment** | **Control** |
| **Age group(years)** |  |  |  |  |
| 15–24 | 1.00 | 1.00 | 1.00 | 1.00 |
| 25–34 | 2.37 (1.86–3.03) *** | 1.66 (0.70–3.93) | 0.66 (0.18–2.43) | 0.50 (0.12–2.14) |
| 35–44 | 4.66 (3.62–5.99) *** | 3.00 (1.28–7.04) * | 2.02 (0.55–7.44) | 0.94 (0.24–3.72) |
| 45–49 | 7.07 (5.82–10.20) *** | 4.17 (1.78–9.76) ** | 3.28 (0.97–11.15) *** | 1.27 (0.31–5.25) |
| **Sex** |  |  |  |  |
| Men | 1.00 | 1.00 | 1.00 | 1.00 |
| Women | 0.68 (0.57–0.81) *** | 1.44 (1.04–1.99) * | 1.76 (1.12–2.76) * | 1.32 (0.73–2.39) |
| **Educational status** |  |  |  |  |
| No formal education | 1.00 | 1.00 | 1.00 | 1.00 |
| Primary education | 1.25 (1.00–1.55) * | 1.34 (0.89–2.01) | 1.50 (0.84–2.66) | 1.12 (0.50–2.53) |
| Secondary education | 1.30 (1.04–1.62) * | 0.94 (0.62–1.44) | 1.07 (0.57–2.01) | 0.78 (0.35–1.74) |
| Higher education | 1.23 (0.91–1.65) | 1.35 (0.78–2.32) | 1.59 (0.70–3.62) | 1.62 (0.63–4.20) |
| **Marital status** |  |  |  |  |
| Never married | 1.00 | 1.00 | 1.00 | 1.00 |
| Married | 1.25 (0.90–1.74) | 2.72 (1.10–6.68) * | 1.97 (0.51–7.63) | 1.91 (0.43–8.55) |
| Widowed | 1.66 (1.14–2.44) ** | 2.42 (0.94–6.24) | 2.17 (0.50–9.42) | 1.63 (0.31–8.55) |
| Divorced | 1.23 (0.65–2.34) | 0.66 (0.14–3.18) | 1.68 (0.19–14.45) | 0.85 (0.05–13.63) |
| **Tobacco consumption** |  |  |  |  |
| No | 1.00 | 1.00 | 1.00 | 1.00 |
| Yes | 1.16 (0.96–1.39) | 0.76 (0.45–1.28) | 0.56 (0.25–1.22) | 0.28 (0.08–0.98) * |
|  |  |  |  |  |
| **Alcohol consumption** |  |  |  |  |
| No | 1.00 | 1.00 | 1.00 | 1.00 |
| Yes | 1.09 (0.69–1.71) | 0.81 (0.34–1.92) | 0.41 (0.11–1.53) | 0.07 (0.00–1.66) |
| **Body mass index** |  |  |  |  |
| Normal | 1.00 | 1.00 | 1.00 | 1.00 |
| Underweight | 0.56 (0.46–0.68) *** | 0.57 (0.34–0.95) * | 0.46 (0.22–0.99) * | 0.54 (0.20–1.50) |
| Overweight | 2.59 (2.20–3.05) *** | 1.62 (1.19–2.21) ** | 1.42 (0.88–2.29) | 0.71 (0.36–1.40) |
| Obese | 3.83 (2.87–5.11) *** | 2.37 (1.33–4.22) ** | 2.20 (1.13–4.29) * | 1.36 (0.60–3.05) |
| **Wealth Quintile** |  |  |  |  |
| Q1 (Poorest) | 1.00 | 1.00 | 1.00 | 1.00 |
| Q2 | 0.93 (0.76–1.14) | 1.57 (1.00–2.48) | 1.64 (0.77–3.46) | 1.54 (0.54–4.42) |
| Q3 | 1.09 (0.87–1.37) | 2.02 (1.26–3.24) ** | 2.90 (1.38–6.10) ** | 3.95 (1.37–11.41) * |
| Q4 | 1.02 (0.79–1.31) | 2.15 (1.33–3.47) ** | 3.23 (1.45–7.20) ** | 4.61 (1.50–14.12) ** |
| Q5 (Richest) | 1.14 (0.87–1.50) | 3.70 (2.20–6.21) *** | 9.75 (3.97–23.92) *** | 10.28 (2.98–35.49) *** |
| **Place of residence** |  |  |  |  |
| Rural | 1.00 | 1.00 | 1.00 | 1.00 |
| Urban | 1.20 (1.00–1.46) | 1.48 (1.08–2.02) * | 2.21 (1.34–3.63) ** | 1.74 (0.90–3.39) |

*** p-value < 0.001 ** p-value < 0.01 * p-value < 0.0

**Supplementary Table 5. Wealth–based inequality in prevalence of hypertension, India, 2016**

| **Categorization** | **Prevalence of hypertension** | | **SII (95% CI)** | **RII (95% CI)** | **CIX*100 (95% CI)** |
| --- | --- | --- | --- | --- | --- |
|  | **Q1 (95% CI)** | **Q5 (95% CI)** |  |  |  |
| **National level** | 9.4 (9.3–9.6) | 13.4 (13.2–13.5) | 5.22 (4.77–5.67) | 1.53 (1.48–1.59) | 7.28 (6.75–7.81) |
| **Place of residence** |  |  |  |  |  |
| Urban | 10.4 (9.7–11.1) | 13.1 (13.0–13.3) | 2.63 (1.74–3.52) | 1.22 (1.14–1.30) | 3.79 (2.71–4.88) |
| Rural | 9.4 (9.2–9.5) | 14.0 (13.7–14.3) | 4.71 (4.27–5.15) | 1.50 (1.45–1.56) | 6.21 (5.67–6.75) |
| **Region** |  |  |  |  |  |
| North | 7.2 (6.6–7.8) | 14.1 (13.8–14.4) | 4.44 (3.51–5.38) | 1.41 (1.31–1.52) | 10.37 (9.36–11.39) |
| Central | 9.0 (8.7–9.2) | 11.9 (11.6–12.3) | 3.19 (2.56–3.82) | 1.36 (1.28–1.44) | 4.77 (4.02–5.53) |
| East | 9.3 (9.1–9.5) | 13.2 (12.5–13.8) | 3.51 (2.52–4.51) | 1.36 (1.25–1.48) | 6.62 (5.45–7.79) |
| Northeast | 16.6 (15.6–17.7) | 19.4 (17.7–21.1) | 4.07 (2.56–5.58) | 1.25 (1.15–1.36) | 4.97 (3.17–6.76) |
| West | 9.3 (8.7–10.0) | 14.2 (13.8–14.6) | 5.98 (4.49–7.47) | 1.59 (1.41–1.76) | 8.15 (6.28–10.01) |
| South | 11.6 (10.8–12.4) | 12.9 (12.5–13.2) | 4.15 (3.08–5.22) | 1.37 (1.26–1.48) | 4.26 (2.94–5.58) |
| **State** |  |  |  |  |  |
| Andaman and Nicobar Islands | 14.0 (8.3–19.7) | 15.5 (12.7–18.3) | 4.69 (-2.10–11.47) | 1.38 (0.75–2.02) | 8.23 (-0.07–16.52) |
| Andhra Pradesh | 12.4 (9.2–15.5) | 16.4 (14.7–18.1) | 8.22 (5.58–10.86) | 1.81 (1.47–2.15) | 11.41 (8.01–14.81) |
| Arunachal Pradesh | 17.7 (16.2–19.1) | 20.2 (18.1–22.4) | 3.33 (0.46–6.21) | 1.20 (1.01–1.39) | 3.67 (0.27–7.07) |
| Assam | 17.8 (16.9–18.7) | 20.6 (18.7–22.5) | 4.04 (1.99–6.09) | 1.24 (1.10–1.37) | 4.74 (2.31–7.16) |
| Bihar | 7.6 (7.3–8.0) | 9.0 (7.7–10.3) | 2.79 (1.65–3.93) | 1.35 (1.18–1.51) | 3.64 (2.23–5.05) |
| Chandigarh | 0.0 (0.0–0.0) | 12.9 (10.3–15.5) | 22.18 (4.66–39.71) | 5.79 (-1.63–13.20) | 14.68 (3.16–26.20) |
| Chhattisgarh | 10.9 (10.3–11.6) | 12.2 (11.2–13.1) | -1.39 (-3.32–0.55) | 0.88 (0.73–1.04) | -1.42 (-3.63–0.79) |
| Dadra and Nagar Haveli | 5.1 (2.0–8.2) | 15.6 (10.2–20.9) | 12.67 (5.99–19.36) | 3.24 (1.17–5.30) | 17.80 (8.37–27.24) |
| Daman and Diu | 0.0 (0.0–0.0) | 11.5 (8.9–14.0) | 8.21 (1.22–15.19) | 2.08 (0.87–3.29) | 9.24 (-0.13–18.60) |
| Goa | 0.0 (0.0–0.0) | 14.1 (12.2–16.0) | 5.18 (0.34–10.02) | 1.47 (0.93–2.00) | 6.72 (-0.33–13.76) |
| Gujarat | 9.2 (8.1–10.3) | 14.8 (14.0–15.7) | 7.78 (5.87–9.70) | 1.84 (1.57–2.11) | 11.19 (8.57–13.82) |
| Haryana | 13.8 (10.2–17.3) | 14.9 (14.3–15.5) | 2.94 (0.17–5.70) | 1.22 (0.99–1.45) | 4.78 (2.32–7.24) |
| Himachal Pradesh | 17.2 (11.8–22.6) | 17.6 (16.3–18.9) | 4.52 (1.75–7.29) | 1.31 (1.09–1.53) | 5.71 (1.69–9.73) |
| Jammu & Kashmir | 14.3 (13.0–15.7) | 16.5 (15.5–17.5) | 4.08 (1.74–6.42) | 1.28 (1.10–1.45) | 5.55 (2.67–8.42) |
| Jharkhand | 9.2 (8.7–9.6) | 10.6 (9.5–11.7) | 1.13 (-0.26–2.53) | 1.12 (0.97–1.27) | 1.69 (-0.13–3.51) |
| Karnataka | 9.7 (8.3–11.0) | 13.9 (12.8–15.0) | 1.66 (-1.11–4.42) | 1.14 (0.90–1.38) | 2.09 (-0.66–4.84) |
| Kerala | 15.8 (6.2–25.3) | 9.4 (8.6–10.2) | -2.07 (-4.20–0.06) | 0.82 (0.65–0.99) | -2.05 (-4.91–0.80) |
| Lakshadweep | 0.0 (0.0–0.0) | 12.8 (9.9–15.7) | -4.19 (-12.38–4.00) | 0.75 (0.32–1.18) | -2.54 (-12.61–7.53) |
| Madhya Pradesh | 9.0 (8.6–9.4) | 13.0 (12.4–13.6) | 4.69 (3.63–5.75) | 1.54 (1.40–1.69) | 6.79 (5.53–8.04) |
| Maharashtra | 9.9 (8.9–10.9) | 14.5 (13.7–15.3) | 5.11 (3.06–7.16) | 1.48 (1.25–1.70) | 7.05 (4.43–9.67) |
| Manipur | 10.3 (8.8–11.9) | 15.5 (13.4–17.5) | 3.29 (0.82–5.76) | 1.26 (1.04–1.47) | 3.54 (0.36–6.72) |
| Meghalaya | 11.6 (9.6–13.7) | 15.0 (12.3–17.6) | 3.63 (-0.22–7.49) | 1.28 (0.95–1.62) | 6.63 (2.67–10.58) |
| Mizoram | 10.0 (7.8–12.2) | 13.2 (12.1–14.3) | 3.96 (0.18–7.73) | 1.34 (0.98–1.70) | 5.13 (0.36–9.90) |
| Nagaland | 13.3 (11.4–15.3) | 19.6 (17.4–21.8) | 7.91 (5.06–10.76) | 1.55 (1.30–1.79) | 11.33 (7.46–15.20) |
| New Delhi | 0.0 (0.0–0.0) | 10.0 (9.0–11.0) | 2.59 (-1.64–6.82) | 1.32 (0.71–1.92) | 2.67 (-2.06–7.41) |
| Odisha | 10.4 (9.9–10.9) | 15.4 (14.1–16.7) | 3.75 (1.92–5.59) | 1.34 (1.15–1.53) | 4.83 (2.77–6.89) |
| Puducherry | 16.7 (8.0–25.3) | 13.9 (12.3–15.4) | 7.95 (1.66–14.24) | 1.81 (0.95–2.68) | 11.63 (3.53–19.72) |
| Punjab | 11.5 (6.2–16.8) | 17.0 (16.4–17.7) | 1.90 (-0.64–4.43) | 1.12 (0.95–1.29) | 2.15 (-0.79–5.08) |
| Rajasthan | 5.6 (5.1–6.1) | 11.4 (10.8–12.0) | 7.45 (6.25–8.65) | 2.12 (1.87–2.37) | 10.78 (9.34–12.22) |
| Sikkim | 23.1 (6.6–39.6) | 22.9 (19.6–26.1) | 5.51 (0.83–10.20) | 1.31 (1.01–1.61) | 8.18 (2.52–13.83) |
| Tamil Nadu | 10.1 (8.3–11.9) | 15.3 (14.4–16.1) | 5.65 (4.02–7.28) | 1.54 (1.35–1.72) | 7.74 (5.46–10.02) |
| Tripura | 9.6 (7.5–11.7) | 20.8 (16.1–25.4) | 8.00 (3.70–12.30) | 1.63 (1.18–2.08) | 9.33 (3.93–14.72) |
| Uttar Pradesh | 8.1 (7.8–8.4) | 11.4 (10.9–11.8) | 3.66 (2.82–4.50) | 1.43 (1.31–1.55) | 5.41 (4.39–6.43) |
| Uttarakhand | 9.0 (7.0–11.0) | 15.4 (14.4–16.4) | 6.72 (4.38–9.07) | 1.65 (1.36–1.94) | 9.60 (6.73–12.46) |
| West Bengal | 11.3 (10.4–12.2) | 14.7 (12.8–16.5) | 5.45 (3.16–7.75) | 1.53 (1.26–1.79) | 7.60 (4.86–10.33) |
| Telangana | 11.8 (9.1–14.5) | 14.5 (12.6–16.3) | 4.05 (0.94–7.15) | 1.31 (1.04–1.59) | 5.46 (0.81–10.11) |

Note: Q1, poorest; Q5, richest; CI, confidence interval; SII, slope index of inequality; RII, relative index of inequality; CIX, concentration index

**Supplementary Table 6. Wealth–based inequality in awareness of hypertension, India, 2016**

| **Categorization** | **Awareness of hypertension** | | **SII (95% CI)** | **RII (95% CI)** | **CIX*100 (95% CI)** |
| --- | --- | --- | --- | --- | --- |
|  | **Q1 (95% CI)** | **Q5 (95% CI)** |  |  |  |
| **National level** | 19.2 (18.5–19.9) | 37.7 (37.0–38.3) | 20.25 (18.47–22.03) | 1.95 (1.84–2.07) | 11.91 (11.08–12.74) |
| **Place of residence** |  |  |  |  |  |
| Urban | 19.1 (16.2–21.9) | 37.9 (37.1–38.6) | 12.20 (8.77–15.64) | 1.42 (1.28–1.56) | 8.43 (6.73–10.12) |
| Rural | 19.2 (18.5–19.9) | 37.1 (35.9–38.3) | 18.82 (16.90–20.75) | 1.98 (1.84–2.12) | 10.48 (9.62–11.34) |
| **Region** |  |  |  |  |  |
| North | 21.9 (21.9–21.9) | 40.0 (40.0–40.0) | 9.06 (5.32–12.80) | 1.29 (1.15–1.42) | 9.00 (7.43–10.57) |
| Central | 13.5 (13.5–13.5) | 33.6 (33.6–33.6) | 25.16 (22.59–27.73) | 2.87 (2.55–3.19) | 13.30 (12.06–14.54) |
| East | 23.7 (23.7–23.7) | 48.0 (48.0–48.0) | 22.02 (17.94–26.09) | 2.06 (1.77–2.34) | 11.76 (9.78–13.74) |
| Northeast | 18.8 (18.8–18.8) | 37.4 (37.4–37.4) | 22.09 (18.32–25.87) | 2.17 (1.87–2.46) | 11.81 (9.96–13.66) |
| West | 11.9 (11.9–11.9) | 28.4 (28.4–28.4) | 12.32 (7.55–17.08) | 1.66 (1.34–1.98) | 7.24 (4.58–9.91) |
| South | 23.4 (23.3–23.4) | 42.9 (42.9–42.9) | 12.99 (8.59–17.39) | 1.43 (1.26–1.60) | 8.24 (6.13–10.34) |
| **State** |  |  |  |  |  |
| Andaman and Nicobar Islands | 60.0 (37.9–82.1) | 44.0 (34.2–53.8) | 11.17 (–14.77–37.11) | 1.34 (0.43–2.25) | 7.21 (–4.70–19.11) |
| Andhra Pradesh | 20.8 (9.7–31.8) | 38.6 (33.2–44.0) | 16.18 (6.51–25.85) | 1.67 (1.16–2.18) | 8.08 (3.14–13.03) |
| Arunachal Pradesh | 21.0 (17.3–24.6) | 35.1 (29.5–40.8) | 15.09 (7.63–22.55) | 1.74 (1.27–2.22) | 8.51 (5.04–11.98) |
| Assam | 17.4 (15.3–19.4) | 37.1 (32.1–42.1) | 21.04 (16.17–25.91) | 2.20 (1.79–2.62) | 10.20 (7.81–12.59) |
| Bihar | 25.5 (23.5–27.5) | 37.6 (30.3–44.8) | 14.25 (7.52–20.98) | 1.59 (1.23–1.94) | 6.98 (4.02–9.95) |
| Chandigarh | 0.0 (0.0–0.0) | 42.0 (31.0–52.9) | 39.62 (–11.83–91.07) | 2.83 (–1.18–6.83) | 11.00 (–9.85–31.85) |
| Chhattisgarh | 7.5 (5.9–9.1) | 30.7 (26.8–34.6) | 29.66 (23.50–35.82) | 6.02 (3.69–8.36) | 14.96 (12.07–17.86) |
| Dadra and Nagar Haveli | 20.0 (–6.5–46.5) | 42.9 (24.0–61.8) | 31.07 (–2.82–64.97) | 3.29 (–1.72–8.29) | 15.84 (–2.17–33.85) |
| Daman and Diu | 0.0 (0.0–0.0) | 21.1 (11.5–30.8) | –0.94 (–25.55–23.67) | 0.97 (0.15–1.79) | –2.01 (–20.40–16.38) |
| Goa | 0.0 (0.0–0.0) | 26.4 (19.9–32.8) | –0.83 (–26.66–25.01) | 0.97 (0.04–1.90) | 2.21 (–7.76–12.17) |
| Gujarat | 9.0 (5.5–12.5) | 26.1 (23.3–28.9) | 16.38 (9.24–23.52) | 2.20 (1.50–2.90) | 8.45 (5.31–11.58) |
| Haryana | 32.0 (18.9–45.1) | 42.7 (40.5–45.0) | –2.89 (–14.32–8.54) | 0.94 (0.71–1.17) | –0.16 (–3.72–3.40) |
| Himachal Pradesh | 21.9 (7.3–36.4) | 35.4 (31.6–39.2) | 15.53 (6.18–24.88) | 1.67 (1.16–2.17) | 6.92 (2.14–11.69) |
| Jammu & Kashmir | 41.1 (35.9–46.2) | 44.1 (40.9–47.4) | 2.28 (–6.02–10.58) | 1.05 (0.86–1.24) | 0.97 (–2.61–4.55) |
| Jharkhand | 14.0 (12.1–15.8) | 35.3 (29.9–40.7) | 22.88 (17.02–28.74) | 3.13 (2.19–4.06) | 12.73 (9.81–15.66) |
| Karnataka | 14.0 (9.0–19.0) | 28.4 (24.6–32.2) | 13.98 (7.76–20.20) | 1.81 (1.34–2.27) | 7.33 (3.47–11.19) |
| Kerala | 11.1 (–10.7–32.9) | 41.7 (37.6–45.9) | 5.97 (–5.03–16.98) | 1.17 (0.83–1.50) | 2.08 (–3.48–7.63) |
| Lakshadweep | 0.0 (0.0–0.0) | 36.4 (24.6–48.1) | 10.68 (–14.11–35.47) | 1.41 (0.30–2.52) | 4.44 (–9.48–18.35) |
| Madhya Pradesh | 11.8 (10.3–13.2) | 35.2 (32.9–37.5) | 27.17 (23.11–31.22) | 3.18 (2.62–3.74) | 14.51 (12.54–16.49) |
| Maharashtra | 14.0 (10.3–17.8) | 31.1 (28.3–33.9) | 12.73 (6.06–19.41) | 1.64 (1.23–2.04) | 7.35 (3.56–11.15) |
| Manipur | 17.2 (11.2–23.3) | 31.4 (24.8–38.0) | 15.72 (6.46–24.98) | 1.71 (1.18–2.25) | 7.89 (3.53–12.25) |
| Meghalaya | 32.7 (23.9–41.5) | 52.9 (43.2–62.7) | 20.20 (8.04–32.35) | 1.62 (1.12–2.12) | 9.77 (4.40–15.14) |
| Mizoram | 11.1 (3.8–18.4) | 40.8 (36.5–45.1) | 16.08 (2.85–29.30) | 1.53 (1.00–2.07) | 10.67 (3.15–18.18) |
| Nagaland | 13.5 (8.1–18.8) | 33.7 (27.8–39.7) | 18.29 (10.26–26.31) | 1.85 (1.35–2.36) | 11.29 (7.22–15.37) |
| New Delhi | 0.0 (0.0–0.0) | 42.3 (37.2–47.4) | 39.22 (21.15–57.28) | 3.03 (1.29–4.77) | 12.04 (1.58–22.51) |
| Odisha | 23.2 (21.0–25.4) | 50.1 (45.5–54.7) | 29.09 (22.64–35.54) | 2.26 (1.82–2.70) | 15.90 (12.91–18.88) |
| Puducherry | 58.3 (29.1–87.5) | 46.3 (40.2–52.4) | 2.42 (–24.69–29.54) | 1.05 (0.52–1.58) | –4.68 (–17.90–8.54) |
| Punjab | 43.8 (18.6–68.9) | 39.3 (37.3–41.2) | 11.22 (3.43–19.00) | 1.35 (1.06–1.63) | 5.40 (1.79–9.00) |
| Rajasthan | 15.8 (12.3–19.3) | 34.4 (31.8–37.1) | 20.79 (15.38–26.20) | 2.19 (1.73–2.65) | 11.93 (9.23–14.62) |
| Sikkim | 50.0 (6.1–93.9) | 41.2 (33.3–49.2) | 3.09 (–9.07–15.24) | 1.08 (0.76–1.39) | 4.35 (–1.50–10.20) |
| Tamil Nadu | 35.1 (26.2–44.1) | 50.5 (47.3–53.6) | 9.06 (0.57–17.54) | 1.22 (0.99–1.45) | 4.54 (0.94–8.14) |
| Tripura | 26.8 (16.4–37.1) | 38.3 (25.9–50.8) | 26.62 (14.33–38.91) | 2.20 (1.36–3.05) | 13.56 (7.11–20.01) |
| Uttar Pradesh | 15.2 (13.7–16.6) | 32.4 (30.4–34.3) | 22.59 (18.95–26.23) | 2.44 (2.08–2.80) | 11.91 (10.15–13.67) |
| Uttarakhand | 24.7 (14.7–34.6) | 37.4 (34.0–40.8) | 15.80 (6.43–25.16) | 1.66 (1.17–2.16) | 8.93 (4.84–13.01) |
| West Bengal | 22.8 (19.2–26.3) | 49.5 (42.7–56.4) | 23.98 (15.94–32.02) | 2.18 (1.59–2.78) | 12.05 (7.94–16.17) |
| Telangana | 29.2 (18.1–40.4) | 51.3 (44.3–58.3) | 18.45 (6.87–30.02) | 1.60 (1.13–2.06) | 9.64 (2.94–16.34) |

Note: Q1, poorest; Q5, richest; CI, confidence interval; SII, slope index of inequality; RII, relative index of inequality; CIX, concentration index

**Supplementary Table 7. Wealth–based inequality in treatment of hypertension, India, 2016**

| **Categorization** | **Treatment of hypertension** | | **SII (95% CI)** | **RII (95% CI)** | **CIX*100 (95% CI)** |
| --- | --- | --- | --- | --- | --- |
|  | **Q1 (95% CI)** | **Q5 (95% CI)** |  |  |  |
| **National level** | 20.8 (20.1–21.5) | 31.2 (30.6–31.8) | 11.68 (10.01–13.35) | 1.55 (1.45–1.64) | 7.25 (6.33–8.18) |
| **Place of residence** |  |  |  |  |  |
| Urban | 18.0 (15.2–20.7) | 31.9 (31.2–32.6) | 11.24 (8.08–14.40) | 1.47 (1.31–1.63) | 6.29 (4.47–8.12) |
| Rural | 21.0 (20.3–21.7) | 29.5 (28.3–30.6) | 8.88 (7.02–10.74) | 1.42 (1.32–1.52) | 5.37 (4.41–6.34) |
| **Region** |  |  |  |  |  |
| North | 23.5 (23.5–23.5) | 29.7 (29.7–29.8) | 5.07 (1.54–8.59) | 1.19 (1.04–1.34) | 3.15 (1.42–4.89) |
| Central | 16.0 (16.0–16.0) | 27.0 (27.0–27.0) | 14.37 (11.85–16.89) | 1.92 (1.70–2.14) | 8.39 (6.99–9.78) |
| East | 25.0 (25.0–25.0) | 38.8 (38.8–38.8) | 13.33 (9.16–17.50) | 1.61 (1.37–1.85) | 6.11 (3.86–8.37) |
| Northeast | 16.2 (16.2–16.2) | 28.2 (28.2–28.2) | 14.69 (11.10–18.28) | 1.94 (1.62–2.26) | 8.29 (6.31–10.27) |
| West | 17.0 (17.0–17.0) | 28.0 (28.0–28.0) | 8.13 (3.44–12.83) | 1.39 (1.13–1.65) | 4.73 (1.76–7.71) |
| South | 24.6 (24.6–24.6) | 36.3 (36.3–36.3) | 8.83 (4.84–12.82) | 1.33 (1.16–1.50) | 7.09 (4.81–9.38) |
| **State** |  |  |  |  |  |
| Andaman and Nicobar Islands | 60.0 (37.9–82.1) | 36.0 (26.5–45.5) | –1.06 (–27.49–25.38) | 0.97 (0.28–1.67) | 2.61 (–10.75–15.96) |
| Andhra Pradesh | 20.8 (9.7–31.8) | 32.6 (27.4–37.8) | 11.43 (1.93–20.93) | 1.54 (0.99–2.09) | 6.43 (1.02–11.83) |
| Arunachal Pradesh | 15.8 (12.5–19.0) | 25.4 (20.2–30.5) | 9.98 (4.04–15.93) | 1.70 (1.17–2.24) | 6.55 (3.11–9.99) |
| Assam | 13.9 (12.0–15.8) | 27.5 (22.9–32.1) | 13.78 (9.26–18.30) | 1.93 (1.51–2.35) | 8.05 (5.54–10.55) |
| Bihar | 32.1 (30.0–34.3) | 33.5 (26.5–40.6) | 2.42 (–4.34–9.18) | 1.07 (0.86–1.29) | 1.87 (–1.57–5.30) |
| Chandigarh | 0.0 (0.0–0.0) | 27.2 (17.3–37.0) | 50.41 (–14.84–115.66) | 7.28 (–13.40–27.96) | –4.70 (–30.71–21.32) |
| Chhattisgarh | 9.9 (8.0–11.7) | 26.1 (22.4–29.8) | 22.44 (16.21–28.67) | 4.09 (2.50–5.69) | 12.89 (9.69–16.09) |
| Dadra and Nagar Haveli | 20.0 (–6.5–46.5) | 32.1 (14.3–50.0) | 19.38 (–14.45–53.21) | 2.07 (–0.76–4.89) | 13.76 (–4.49–32.00) |
| Daman and Diu | 16.9 (12.3–21.5) | 29.6 (18.8–40.3) | 7.22 (–22.61–37.06) | 1.22 (0.21–2.23) | 1.20 (–20.80–23.20) |
| Goa | 0.0 (0.0–0.0) | 33.5 (26.6–40.4) | 13.80 (–8.39–35.99) | 1.55 (0.45–2.65) | 6.10 (–6.72–18.92) |
| Gujarat | 0.0 (0.0–0.0) | 25.6 (22.8–28.3) | 12.60 (5.85–19.34) | 1.81 (1.26–2.36) | 7.68 (3.87–11.50) |
| Haryana | 38.0 (24.4–51.6) | 34.4 (32.3–36.6) | –11.33 (–22.89–0.22) | 0.74 (0.51–0.96) | –6.52 (–10.54– -2.50) |
| Himachal Pradesh | 15.6 (2.8–28.4) | 27.3 (23.8–30.9) | 13.58 (5.80–21.37) | 1.79 (1.20–2.39) | 7.94 (3.23–12.64) |
| Jammu & Kashmir | 36.3 (31.2–41.3) | 37.8 (34.6–40.9) | 3.27 (–4.42–10.96) | 1.09 (0.87–1.30) | 1.48 (–2.46–5.43) |
| Jharkhand | 14.7 (12.8–16.6) | 25.8 (20.9–30.7) | 11.24 (5.43–17.05) | 1.87 (1.26–2.48) | 6.40 (3.11–9.69) |
| Karnataka | 21.0 (15.1–26.8) | 26.6 (22.9–30.3) | 9.23 (3.21–15.25) | 1.49 (1.11–1.88) | 4.27 (0.23–8.31) |
| Kerala | 0.0 (0.0–0.0) | 38.2 (34.1–42.3) | 3.95 (–7.22–15.11) | 1.13 (0.75–1.50) | 2.41 (–3.63–8.46) |
| Lakshadweep | 16.3 (14.6–17.9) | 31.8 (20.4–43.2) | 18.65 (–3.31–40.61) | 2.20 (0.18–4.22) | 14.90 (1.34–28.46) |
| Madhya Pradesh | 0.0 (0.0–0.0) | 32.2 (29.9–34.5) | 18.21 (14.01–22.42) | 2.08 (1.73–2.44) | 10.93 (8.58–13.27) |
| Maharashtra | 18.2 (14.1–22.3) | 30.3 (27.6–33.1) | 8.00 (1.92–14.07) | 1.36 (1.05–1.66) | 6.11 (1.86–10.37) |
| Manipur | 7.9 (3.6–12.3) | 20.4 (14.7–26.2) | 14.07 (7.78–20.36) | 2.43 (1.46–3.40) | 9.36 (5.29–13.43) |
| Meghalaya | 40.0 (30.8–49.2) | 38.2 (28.7–47.7) | 8.27 (–4.45–20.99) | 1.23 (0.83–1.63) | 1.31 (–4.99–7.61) |
| Mizoram | 8.3 (1.9–14.8) | 27.1 (23.1–31.0) | 8.24 (–4.78–21.26) | 1.36 (0.72–1.99) | 7.42 (–0.61–15.46) |
| Nagaland | 11.5 (6.5–16.6) | 16.9 (12.2–21.6) | 11.59 (5.32–17.85) | 2.43 (1.29–3.56) | 6.70 (2.89–10.52) |
| New Delhi | 20.4 (18.3–22.5) | 36.7 (31.8–41.7) | 27.27 (9.03–45.51) | 2.24 (0.97–3.51) | 13.03 (0.81–25.25) |
| Odisha | 0.0 (0.0–0.0) | 42.8 (38.2–47.4) | 25.95 (19.73–32.17) | 2.28 (1.79–2.77) | 16.10 (12.70–19.50) |
| Puducherry | 25.0 (–0.6–50.6) | 34.4 (28.6–40.2) | –2.01 (–24.94–20.92) | 0.95 (0.36–1.54) | 0.20 (–15.99–16.38) |
| Punjab | 31.3 (7.8–54.7) | 24.8 (23.0–26.5) | 9.76 (2.78–16.75) | 1.53 (1.06–2.00) | 4.21 (1.08–7.34) |
| Rajasthan | 20.0 (16.1–23.8) | 28.0 (25.5–30.6) | 8.09 (3.05–13.12) | 1.39 (1.10–1.67) | 2.74 (–2.95–8.42) |
| Sikkim | 50.0 (6.1–93.9) | 23.0 (16.2–29.8) | 5.41 (–3.94–14.76) | 1.25 (0.77–1.72) | 5.87 (1.88–9.85) |
| Tamil Nadu | 29.7 (21.2–38.3) | 42.0 (38.9–45.1) | 10.87 (3.68–18.07) | 1.35 (1.08–1.61) | 14.69 (7.72–21.66) |
| Tripura | 25.4 (15.1–35.6) | 35.0 (22.8–47.2) | 22.41 (10.18–34.64) | 2.22 (1.23–3.21) | 6.45 (4.52–8.39) |
| Uttar Pradesh | 16.9 (15.3–18.5) | 24.0 (22.2–25.8) | 10.17 (6.71–13.63) | 1.59 (1.34–1.84) | 3.43 (–1.08–7.93) |
| Uttarakhand | 21.9 (12.4–31.5) | 29.2 (26.0–32.4) | 6.13 (–2.19–14.44) | 1.25 (0.87–1.63) | 8.55 (4.00–13.10) |
| West Bengal | 18.5 (15.2–21.8) | 36.4 (29.8–43.0) | 15.58 (7.22–23.94) | 1.90 (1.25–2.55) | 7.03 (–0.44–14.50) |
| Telangana | 32.3 (20.8–43.8) | 40.6 (33.7–47.5) | 8.30 (–3.45–20.04) | 1.29 (0.83–1.76) | 3.19 (–0.29–6.67) |

Note: Q1, poorest; Q5, richest; CI, confidence interval; SII, slope index of inequality; RII, relative index of inequality; CIX, concentration index

**Supplementary Table 8. Wealth–based inequality in control of hypertension, India, 2016**

| **Categorization** | **Control of hypertension** | | **SII (95% CI)** | **RII (95% CI)** | **CIX*100 (95% CI)** |
| --- | --- | --- | --- | --- | --- |
|  | **Q1 (95% CI)** | **Q5 (95% CI)** |  |  |  |
| **National level** | 16.0 (15.4–16.7) | 19.9 (19.4–20.4) | 6.24 (3.39–9.08) | 1.39 (1.18–1.59) | 3.53 (2.32–4.74) |
| **Place of residence** |  |  |  |  |  |
| Urban | 14.8 (13.4–16.2) | 18.0 (16.3–19.7) | 1.56 (–16.60–19.72) | 1.09 (0.00–2.18) | 4.73 (2.42–7.04) |
| Rural | 19.6 (18.1–21.1) | 17.8 (17.1–18.5) | 6.80 (–1.05–14.65) | 1.47 (0.82–2.12) | 1.92 (0.65–3.18) |
| **Region** |  |  |  |  |  |
| North | 16.2 (13.0–19.3) | 17.7 (16.8–18.7) | –1.10 (–4.20–2.00) | 0.94 (0.79–1.10) | –2.48 (–4.72– -0.23) |
| Central | 13.3 (12.3–14.3) | 17.3 (16.1–18.5) | 5.06 (2.83–7.28) | 1.36 (1.18–1.54) | 4.65 (2.81–6.49) |
| East | 19.3 (18.3–20.3) | 22.7 (20.6–24.8) | 5.31 (1.83–8.79) | 1.32 (1.08–1.56) | –0.37 (–3.17–2.43) |
| Northeast | 8.9 (7.0–10.9) | 15.3 (11.8–18.8) | 6.01 (3.12–8.89) | 1.65 (1.25–2.05) | 5.73 (3.45–8.00) |
| West | 14.2 (11.8–16.7) | 19.1 (18.0–20.3) | 2.24 (–2.22–6.70) | 1.13 (0.85–1.42) | 3.89 (–0.20–7.97) |
| South | 17.1 (14.4–19.8) | 24.1 (23.0–25.2) | 3.92 (0.25–7.59) | 1.21 (1.00–1.42) | 6.11 (3.01–9.21) |
| **State** |  |  |  |  |  |
| Andaman and Nicobar Islands | 40.0 (17.9–62.1) | 15.0 (8.0–22.0) | 1.55 (–17.00–20.11) | 1.09 (–0.02–2.20) | –1.52 (–14.74–11.70) |
| Andhra Pradesh | 13.2 (4.0–22.4) | 22.8 (18.1–27.4) | 6.79 (–1.00–14.59) | 1.47 (0.82–2.11) | 0.60 (–4.85–6.06) |
| Arunachal Pradesh | 9.5 (6.9–12.2) | 10.1 (6.6–13.7) | 1.40 (–2.89–5.68) | 1.14 (0.67–1.61) | –1.31 (–4.60–1.97) |
| Assam | 7.1 (5.7–8.5) | 13.5 (9.9–17) | 5.47 (1.97–8.97) | 1.69 (1.12–2.26) | 3.27 (0.79–5.76) |
| Bihar | 27.3 (25.3–29.4) | 24.9 (18.4–31.3) | –5.27 (–11.69–1.14) | 0.82 (0.63–1.02) | 2.69 (–0.91–6.29) |
| Chandigarh | 0.0 (0.0–0.0) | 14.8 (6.9–22.7) | 15.83 (–39.55–71.21) | 2.87 (–7.33–13.07) | 7.94 (–10.99–26.87) |
| Chhattisgarh | 6.8 (5.3–8.4) | 15 (12–18) | 12.10 (6.91–17.29) | 2.98 (1.62–4.35) | 9.53 (6.09–12.97) |
| Dadra and Nagar Haveli | 20.0 (–6.5–46.5) | 14.3 (0.9–27.6) | –1.13 (–35.24–32.98) | 0.94 (–0.86–2.73) | 3.87 (–14.76–22.49) |
| Daman and Diu | 0.0 (0.0–0.0) | 22.5 (12.7–32.4) | 2.55 (–27.80–32.90) | 1.09 (–0.02–2.20) | –1.20 (–18.94–16.54) |
| Goa | 0.0 (0.0–0.0) | 22 (15.9–28) | 10.70 (–5.41–26.80) | 1.66 (0.37–2.95) | 0.54 (–11.42–12.50) |
| Gujarat | 15.3 (10.9–19.7) | 18.2 (15.8–20.6) | 7.55 (1.36–13.74) | 1.65 (1.01–2.29) | 3.07 (–0.61–6.74) |
| Haryana | 32.0 (18.9–45.1) | 24.2 (22.2–26.1) | –15.23 (–25.61– -4.85) | 0.57 (0.36–0.78) | –5.01 (–8.94––1.07) |
| Himachal Pradesh | 12.5 (0.9–24.1) | 15.5 (12.6–18.4) | 4.67 (–1.92–11.25) | 1.39 (0.75–2.02) | –5.05 (–9.83––0.27) |
| Jammu & Kashmir | 25.2 (20.7–29.8) | 24.7 (21.9–27.5) | 0.00 (–6.58–6.59) | 1.00 (0.74–1.26) | –6.89 (–10.80––2.97) |
| Jharkhand | 11.8 (10.1–13.6) | 15.4 (11.3–19.4) | 3.91 (–0.91–8.73) | 1.33 (0.86–1.80) | 6.99 (3.81–10.16) |
| Karnataka | 15.6 (10.4–20.8) | 16.5 (13.4–19.5) | 5.08 (–0.46–10.63) | 1.41 (0.90–1.91) | 0.09 (–3.85–4.03) |
| Kerala | 0.0 (0.0–0.0) | 28.3 (24.5–32.1) | 5.18 (–5.01–15.38) | 1.24 (0.72–1.75) | –0.07 (–6.45–6.31) |
| Lakshadweep | 0.0 (0.0–0.0) | 18.2 (8.7–27.6) | 8.69 (–12.97–30.34) | 1.83 (–0.91–4.57) | –2.09 (–14.83–10.64) |
| Madhya Pradesh | 13.6 (12.0–15.1) | 19.6 (17.6–21.5) | 6.44 (2.86–10.02) | 1.43 (1.15–1.72) | 9.93 (7.51–12.35) |
| Maharashtra | 14.3 (10.6–18.1) | 20.1 (17.7–22.5) | 1.71 (–4.35–7.77) | 1.09 (0.75–1.44) | 3.38 (–0.95–7.70) |
| Manipur | 3.3 (0.4–6.2) | 11.5 (7–16.1) | 7.73 (2.24–13.22) | 2.45 (0.98–3.91) | 2.15 (–1.63–5.92) |
| Meghalaya | 32.7 (23.9–41.5) | 27.5 (18.7–36.2) | 0.84 (–12.55–14.23) | 1.03 (0.55–1.50) | 11.46 (5.44–17.49) |
| Mizoram | 4.2 (–0.5–8.8) | 15.2 (12–18.3) | 0.95 (–10.24–12.14) | 1.06 (0.39–1.73) | 5.87 (–1.94–13.68) |
| Nagaland | 7.1 (3.0–11.1) | 5.8 (2.8–8.7) | 2.28 (–1.75–6.30) | 1.48 (0.46–2.50) | 0.07 (–3.28–3.42) |
| New Delhi | 0.0 (0.0–0.0) | 21.8 (17.6–26.1) | 8.42 (–8.58–25.42) | 1.44 (0.37–2.51) | 5.99 (–5.40–17.38) |
| Odisha | 15.5 (13.6–17.3) | 29.7 (25.5–33.9) | 17.56 (11.95–23.17) | 2.11 (1.58–2.64) | 9.53 (6.05–13.01) |
| Puducherry | 16.7 (–5.4–38.7) | 21.2 (16.2–26.2) | 9.92 (–9.35–29.19) | 1.55 (0.27–2.83) | –0.72 (–16.13–14.69) |
| Punjab | 12.5 (–4.2–29.2) | 11.1 (9.8–12.4) | 0.24 (–4.61–5.09) | 1.02 (0.58–1.46) | –2.40 (–5.76–0.97) |
| Rajasthan | 13.6 (10.3–16.9) | 18.4 (16.2–20.5) | 1.11 (–3.54–5.75) | 1.06 (0.79–1.34) | 1.39 (–1.67–4.46) |
| Sikkim | 16.7 (–16.0–49.4) | 10.1 (5.3–15) | 2.62 (–4.16–9.41) | 1.28 (0.47–2.08) | –0.31 (–6.10–5.49) |
| Tamil Nadu | 16.7 (–16.0–49.4) | 10.1 (5.3–15) | 2.14 (–4.97–9.25) | 1.09 (0.78–1.40) | 3.69 (–0.30–7.69) |
| Tripura | 16.9 (8.1–25.7) | 21.7 (11.1–32.2) | 10.73 (–0.32–21.79) | 1.80 (0.72–2.88) | 1.82 (–5.20–8.84) |
| Uttar Pradesh | 14.2 (12.8–15.7) | 15.5 (14–17) | 2.68 (–0.48–5.83) | 1.17 (0.95–1.40) | 5.54 (3.56–7.53) |
| Uttarakhand | 15.1 (6.8–23.3) | 17.4 (14.7–20.1) | –0.67 (–7.27–5.93) | 0.96 (0.61–1.32) | –4.56 (–8.95– -0.17) |
| West Bengal | 10.7 (8.1–13.4) | 17.5 (12.3–22.7) | 6.00 (–0.31–12.32) | 1.60 (0.82–2.39) | 6.06 (1.81–10.31) |
| Telangana | 21.5 (11.5–31.6) | 24.9 (18.8–30.9) | 7.75 (–3.15–18.66) | 1.45 (0.71–2.18) | 0.15 (–7.77–8.07) |

Note: Q1, poorest; Q5, richest; CI, confidence interval; SII, slope index of inequality; RII, relative index of inequality; CIX, concentration index

**Supplementary Table 9. Wealth–based inequality in prevalence of hypertension, Nepal, 2016**

| **Categorization** | **Prevalence of hypertension** | | | **SII (95% CI)** | | **RII (95% CI)** | | **CIX*100 (95% CI)** | |
| --- | --- | --- | --- | --- | --- | --- | --- | --- | --- |
|  | **Q1 (95% CI)** | **Q5 (95% CI)** | |  |  |  |  |  |  |
| **National** | 18.1 (16.6–19.6) | | 24.5 (23.0–26.0) | | 7.14 (2.90–11.41) | | 1.43 (1.13–1.72) | | 5.73 (3.46–8.01) |
| **Place of residence** |  | |  | |  | |  | |  |
| Urban | 18.0 (15.6–20.4) | | 18.2 (16.0–20.4) | | 12.78 (7.50–17.99) | | 1.83 (1.39–2.28) | | 10.59 (7.41–13.77) |
| Rural | 18.1 (16.2–20.0) | | 28.8 (26.7–30.9) | | –1.52 (–7.24–4.21) | | 0.92 (0.64–1.21) | | –1.40 (–4.55–1.74) |
| **Region** |  | |  | |  | |  | |  |
| Himal | 15.0 (11.3–18.6) | | 24.4 (12.2–36.6) | | 7.30 (–3.12–17.71) | | 1.51 (0.61–2.40) | | 4.82 (–2.70–12.33) |
| Pahaad | 19.5 (17.6–21.5) | | 31.5 (28.8–34.2) | | 13.10 (6.34–19.86) | | 1.78 (1.27–2.28) | | 10.39 (6.40–14.38) |
| Terai | 16.1 (13.2–19.1) | | 20.2 (18.3–22.0) | | 4.72 (0.01–9.44) | | 1.30 (0.96–1.64) | | 3.96 (1.07–6.85) |
| **Province** |  | |  | |  | |  | |  |
| Province 1 | 17.8 (14.6–21.0) | | 28.8 (26.7–30.9) | | 2.37 (–3.66–8.39) | | 1.13 (0.78–1.48) | | 2.37 (–2.63–7.38) |
| Province 2 | 16.8 (11.2–22.3) | | 18.2 (16.0–20.4) | | 2.68 (–4.31–9.65) | | 1.19 (0.66–1.71) | | 2.01 (–2.51–6.52) |
| Province 3 | 19.4 (15.3–23.5) | | 32.6 (29.6–35.6) | | 19.48 (9.14–29.92) | | 2.30 (1.31–3.29) | | 17.26 (10.87–23.65) |
| Province 4 | 28.4 (23.1–33.7) | | 29.6 (24.1–35.1) | | 0.89 (–8.73–10.5) | | 1.03 (0.67–1.40) | | –0.40 (–6.71–5.90) |
| Province 5 | 20.6 (16.4–24.8) | | 20.1 (16.6–23.5) | | –0.69 (–10.89–9.51) | | 0.97 (0.52–1.42) | | 0.02 (–5.08–5.12) |
| Province 6 | 12.8 (9.7–15.8) | | 42.2 (20.4–63.9) | | 13.50 (2.39–24.6) | | 2.40 (0.65–4.15) | | 11.82 (6.62–17.02) |
| Province 7 | 14.2 (10.7–17.7) | | 19.3 (13.1–25.5) | | 3.12 (–4.85–11.11) | | 1.25 (0.54–1.96) | | 3.84 (–1.28–8.97) |

Note: Q1, poorest; Q5, richest; CI, confidence interval; SII, slope index of inequality; RII, relative index of inequality; CIX, concentration index

**Supplementary Table 10. Wealth–based inequality in awareness of hypertension, Nepal, 2016**

| **Categorization** | **Awareness of hypertension** | | **SII (95% CI)** | **RII (95% CI)** | **CIX*100 (95% CI)** |
| --- | --- | --- | --- | --- | --- |
|  | **Q1 (95% CI)** | **Q5 (95% CI)** |  |  |  |
| **National** | 26.8 (22.8–30.9) | 51.7 (48.1–55.4) | 28.92 (21.27–36.57) | 2.10 (1.67–2.52) | 11.44 (8.48–14.40) |
| **Place of residence** |  |  |  |  |  |
| Urban | 30.7 (25.4–36.0) | 54.1 (49.8–58.4) | 28.14 (18.12–38.15) | 1.97 (1.48–2.45) | 11.631 (7.87–15.39) |
| Rural | 20.3 (14.3–26.4) | 46.3 (39.7–52.9) | 28.91 (17.27–40.56) | 2.32 (1.51–3.12) | 12.037 (7.51–16.57) |
| **Region** |  |  |  |  |  |
| Himal | 19.8 (9.1–30.4) | 71.1 (44.0–98.2) | 47.02 (23.88 –70.17) | 4.12 (0.92–7.32) | 19.45 (8.92–29.98) |
| Pahaad | 25.9 (21.0–30.8) | 50.3 (45.1–55.5) | 30.33 (19.37 –41.29) | 2.23 (1.57–2.89) | 12.68 (8.23–17.14) |
| Terai | 33.7 (24.3–43.2) | 52.6 (47.5–57.7) | 24.60 (12.62 –36.58) | 1.82 (1.27–2.38) | 9.53 (5.26–13.80) |
| **Province** |  |  |  |  |  |
| Province 1 | 26.6 (17.8–35.4) | 56.4 (46.2–66.6) | 33.07 (16.98–49.16) | 2.18 (1.29–3.07) | 13.36 (6.41–20.31) |
| Province 2 | 48.5 (30.0–66.9) | 53.0 (43.9–62.2) | 18.08 (–3.45–39.62) | 1.52 (0.75–2.30) | 6.60 (–0.93–14.14) |
| Province 3 | 27.8 (17.1–38.4) | 55.8 (50.3–61.4) | 43.47 (26.08–60.85) | 2.98 (1.53–4.44) | 16.59 (9.77–23.42) |
| Province 4 | 35.9 (25.3–46.5) | 40.9 (29.9–52.0) | 9.17 (–6.11–24.46) | 1.25 (0.77–1.74) | 4.14 (–2.09–10.36) |
| Province 5 | 26.8 (16.5–37.0) | 43.4 (33.9–52.9) | 13.80 (–4.75–32.36) | 1.48 (0.71–2.24) | 5.70 (–0.99–12.38) |
| Province 6 | 17.0 (7.2–26.9) | 45.8 (10.4–81.1) | 32.85 (14.52–51.18) | 3.71 (0.58–6.83) | 9.03 (1.66–16.40) |
| Province 7 | 11.6 (3.0–20.3) | 49.6 (31.3–68.0) | 46.19 (25.66–66.72) | 5.32 (0.91–9.73) | 17.27 (9.53–25.00) |

Note: Q1, poorest; Q5, richest; CI, confidence interval; SII, slope index of inequality; RII, relative index of inequality; CIX, concentration index

**Supplementary Table 11. Wealth–based inequality in treatment of hypertension, Nepal, 2016**

| **Categorization** | **Treatment of hypertension** | | **SII (95% CI)** | **RII (95% CI)** | **CIX*100 (95% CI)** |
| --- | --- | --- | --- | --- | --- |
|  | **Q1 (95% CI)** | **Q5 (95% CI)** |  |  |  |
| **National** | 9.3 (6.7–12.0) | 33.1 (29.7–36.5) | 30.24 (23.39–37.08) | 4.57 (3.00–6.13) | 23.29 (18.41–28.17) |
| **Place of residence** |  |  |  |  |  |
| Urban | 12.3 (8.5–16.0) | 35.4 (31.3–39.5) | 30.28 (21.25–39.32) | 3.86 (2.28–5.43) | 23.40 (16.94–29.85) |
| Rural | 4.5 (1.4–7.5) | 27.8 (21.9–33.7) | 27.09 (16.97–37.21) | 5.79 (2.29–9.28) | 21.09 (14.21–27.97) |
| **Region** |  |  |  |  |  |
| Himal | 4.5 (–1.0–10.0) | 57.9 (28.4–87.4) | 38.41 (5.17–71.64) | 24.82 (–37.12–86.77) | 31.05 (15.49–46.61) |
| Pahaad | 7.1 (4.2–10.0) | 31.1 (26.3–35.9) | 32.67 (23.77–41.58) | 6.03 (3.07–9.00) | 24.05 (16.85–31.24) |
| Terai | 19.3 (11.4–27.1) | 34.3 (29.4–39.1) | 22.04 (10.77–33.30) | 2.60 (1.32–3.88) | 16.26 (8.94–23.57) |
| **Province** |  |  |  |  |  |
| Province 1 | 8.1 (2.7–13.5) | 45.6 (35.3–55.8) | 43.48 (29.56–57.41) | 7.56 (1.40–13.73) | 33.42 (21.66–45.18) |
| Province 2 | 35.2 (17.6–52.8) | 31.5 (23.0–40.1) | 15.91 (–2.86–34.69) | 1.90 (0.52–3.29) | 12.80 (–0.42–26.01) |
| Province 3 | 5.1 (–0.1–10.3) | 35.0 (29.6–40.3) | 43.36 (27.66–59.05) | 7.35 (1.65–13.04) | 28.93 (18.28–39.59) |
| Province 4 | 14.3 (6.5–22.0) | 23.6 (14.0–33.1) | 15.67 (3.26–28.09) | 2.31 (0.65–3.97) | 10.47 (0.34–20.60) |
| Province 5 | 5.1 (0.0–10.2) | 30.0 (21.3–38.8) | 18.15 (2.80–33.49) | 3.14 (0.12–6.17) | 15.11 (5.38–24.85) |
| Province 6 | 5.2 (–0.6–11.0) | 6.8 (–11.1–24.7) | 8.34 (–3.40–20.08) | 2.79 (–1.20–6.78) | 6.54 (–0.59–13.66) |
| Province 7 | 5.9 (–0.4–12.3) | 25.6 (9.6–41.6) | 20.38 (–0.74–41.50) | 4.27 (–1.71–10.26) | 15.17 (2.72–27.63) |

Note: Q1, poorest; Q5, richest; CI, confidence interval; SII, slope index of inequality; RII, relative index of inequality; CIX, concentration index

**Supplementary Table 12. Wealth–based inequality in control of hypertension, Nepal, 2016**

| **Categorization** | **Control of hypertension** | | **SII (95% CI)** | **RII (95% CI)** | **CIX*100 (95% CI)** |
| --- | --- | --- | --- | --- | --- |
|  | **Q1 (95% CI)** | **Q5 (95% CI)** |  |  |  |
| **National** | 4.2 (2.4–6.0) | 17.7 (14.9–20.5) | 18.32 (12.96–23.69) | 5.33 (2.96–7.70) | 27.41 (19.89–34.93) |
| **Place of residence** |  |  |  |  |  |
| Urban | 4.7 (2.2–7.1) | 19.5 (16.1–22.9) | 20.01 (13.17–26.85) | 5.27 (2.45–8.09) | 29.86 (19.87–39.85) |
| Rural | 3.4 (0.7–6.1) | 13.5 (9.0–18.0) | 13.97 (5.51–22.44) | 4.81 (1.03–8.60) | 19.90 (9.88–29.92) |
| **Region** |  |  |  |  |  |
| Himal | 1.1 (–1.7–4.0) | 39.7 (10.4–68.9) | 27.59 (5.66–49.51) | 61.59 (–95.07–218.24) | 31.66 (10.71–52.61) |
| Pahaad | 4.1 (1.9–6.3) | 18.9 (14.8–22.9) | 20.97 (12.87–29.07) | 7.48 (2.36–12.59) | 29.03 (17.27–40.79) |
| Terai | 6.4 (1.5–11.3) | 15.8 (12.1–19.6) | 11.91 (4.27–19.55) | 2.67 (1.02–4.32) | 19.24 (8.74–29.74) |
| **Province** |  |  |  |  |  |
| Province 1 | 1.3 (–1.0–3.5) | 25.8 (16.8–34.8) | 29.66 (18.56–40.76) | 10.17 (0.37–19.97) | 42.33 (24.87–59.80) |
| Province 2 | 17.1 (3.2–31.0) | 16.9 (10.0–23.8) | 12.15 (–0.47–24.77) | 2.40 (0.32–4.49) | 21.87 (2.59–41.15) |
| Province 3 | 5.1 (–0.1–10.3) | 21.1 (16.5–25.7) | 29.55 (15.89–43.21) | 8.58 (0.81–16.35) | 37.40 (19.80–55.00) |
| Province 4 | 5.4 (0.4–10.3) | 10.7 (3.8–17.6) | 6.80 (–2.05–15.66) | 2.20 (–0.10–4.50) | 9.98 (–3.70–23.66) |
| Province 5 | 1.5 (–1.3–4.4) | 6.3 (1.6–10.9) | 2.19 (–4.84–9.21) | 1.51 (–0.50–3.52) | 2.34 (–7.43–12.10) |
| Province 6 | 3.9 (–1.2–8.9) | 6.8 (–11.1–24.7) | 7.83 (–2.90–18.55) | 3.50 (–2.71–9.72) | 11.54 (–1.01–24.09) |
| Province 7 | 3.6 (–1.4–8.6) | 22.6 (7.3–37.9) | 19.2 (–1.93–40.34) | 6.85 (–5.12–18.81) | 26.63 (3.01–50.25) |

Note: Q1, poorest; Q5, richest; CI, confidence interval; SII, slope index of inequality; RII, relative index of inequality; CIX, concentration index

**Supplementary Table 13. Education–based inequality in prevalence of hypertension, India, 2016**

| **Categorization** | **Prevalence of hypertension** | | **SII (95% CI)** | **RII (95% CI)** | **CIX*100 (95% CI)** |
| --- | --- | --- | --- | --- | --- |
|  | **No edu (95% CI)** | **H. edu (95% CI)** |  |  |  |
| **National level** | 14.3 (14.1–14.5) | 9.4 (9.2–9.6) | –6.96 (–7.39– -6.52) | 0.57 (0.54–0.59) | –9.26 (–9.78– -8.74) |
| **Place of residence** |  |  |  |  |  |
| Urban | 17.3 (16.9–17.7) | 10.1 (9.9–10.4) | –8.56 (–9.50– -7.62) | 0.53 (0.49–0.56) | –10.30 (–11.41– -9.19) |
| Rural | 13.6 (13.4–13.7) | 8.3 (8.0–8.6) | –7.73 (–8.17– -7.29) | 0.51 (0.49–0.53) | –10.70 (–11.24– -10.16) |
| **Region** |  |  |  |  |  |
| North | 14.4 (14.4–14.4) | 10.0 (10.0–10.0) | –5.93 (–6.79– -5.07) | 0.63 (0.59–0.67) | –7.25 (–8.23– -6.26) |
| Central | 12.7 (12.7–12.7) | 8.4 (8.4–8.4) | –7.56 (–8.19– -6.94) | 0.48 (0.45–0.51) | –9.74 (–10.47– -9.00) |
| East | 12.6 (12.6–12.6) | 9.6 (9.6–9.6) | –6.15 (–7.13– -5.18) | 0.58 (0.53–0.63) | –8.08 (–9.19– -6.97) |
| Northeast | 23.1 (23.1–23.1) | 16.2 (16.2–16.2) | –10.10 (–11.67– -8.52) | 0.57 (0.52–0.62) | –12.70 (–14.50– -10.90) |
| West | 15.5 (15.5–15.5) | 9.8 (9.8–9.8) | –6.75 (–8.26– -5.25) | 0.59 (0.52–0.66) | –7.95 (–9.85– -6.06) |
| South | 17.1 (17.1–17.1) | 8.8 (8.8–8.8) | –10.88 (–11.95– -9.82) | 0.43 (0.40–0.47) | –13.70 (–15.00– -12.40) |
| **State** |  |  |  |  |  |
| Andaman and Nicobar Islands | 24.4 (19.7–29.2) | 12.7 (9.4–15.9) | –9.42 (–15.06– -3.78) | 0.52 (0.31–0.73) | –7.94 (–16.17–0.30) |
| Andhra Pradesh | 16.0 (14.8–17.2) | 10.1 (8.5–11.8) | –9.70 (–12.30– -7.09) | 0.50 (0.40–0.59) | –12.79 (–16.13– -9.44) |
| Arunachal Pradesh | 25.4 (24.2–26.6) | 18.9 (16.8–21.1) | –11.81 (–14.65– -8.97) | 0.52 (0.43–0.60) | –16.20 (–19.62– -12.78) |
| Assam | 24.6 (23.6–25.6) | 16.3 (14.7–17.9) | –11.67 (–13.74– -9.61) | 0.54 (0.48–0.60) | –15.63 (–18.04– 13.23) |
| Bihar | 9.6 (9.2–10.0) | 7.8 (6.9–8.7) | –4.40 (–5.52– -3.29) | 0.62 (0.55–0.70) | –6.00 (–7.38– -4.62) |
| Chandigarh | 15.9 (8.9–22.9) | 10.4 (6.3–14.6) | –6.69 (–15.86–2.48) | 0.58 (0.16–0.99) | –9.11 (–21.37–3.16) |
| Chhattisgarh | 16.0 (15.2–16.9) | 9.1 (8.0–10.2) | –13.84 (–15.79– -11.88) | 0.29 (0.24–0.33) | –16.10 (–18.30– -13.90) |
| Dadra and Nagar Haveli | 11.6 (7.6–15.5) | 12.1 (6.7–17.6) | –3.14 (–10.69–4.42) | 0.75 (0.24–1.26) | –3.46 (–12.98–6.06) |
| Daman and Diu | 15.7 (10.9–20.6) | 8.2 (4.4–11.9) | –1.72 (–9.38–5.95) | 0.86 (0.28–1.44) | –4.14 (–13.16–4.87) |
| Goa | 14.2 (9.0–19.4) | 11.0 (8.2–13.7) | –0.94 (–6.30–4.42) | 0.93 (0.56–1.30) | –0.91 (–7.77–5.95) |
| Gujarat | 13.5 (12.6–14.4) | 11.1 (10.0–12.2) | –2.51 (–4.32– -0.70) | 0.82 (0.70–0.94) | –3.00 (–5.44– -0.56) |
| Haryana | 17.2 (16.2–18.3) | 11.1 (10.2–12.1) | –2.51 (–4.32– -0.70) | 0.58 (0.49–0.68) | –8.06 (–10.51– -5.61) |
| Himachal Pradesh | 22.3 (19.9–24.8) | 13.2 (11.7–14.7) | –10.38 (–13.72– -7.05) | 0.54 (0.43–0.64) | –13.19 (–17.05– -9.32) |
| Jammu & Kashmir | 22.0 (21.1–22.9) | 11.3 (10.3–12.4) | –10.38 (–13.72– -7.05) | 0.37 (0.31–0.42) | –19.57 (–22.39– -16.75) |
| Jharkhand | 12.2 (11.6–12.8) | 7.8 (6.8–8.9) | –10.38 (–13.72– -7.05) | 0.50 (0.43–0.57) | –8.47 (–10.25– -6.68) |
| Karnataka | 16.1 (15.3–17.0) | 10.4 (9.3–11.4) | –10.19 (–12.27– -8.11) | 0.45 (0.38–0.53) | –13.07 (–15.64– -10.49) |
| Kerala | 19.7 (13.5–26.0) | 6.8 (6.0–7.6) | –9.72 (–12.57– -6.86) | 0.39 (0.28–0.50) | –8.63 (–11.50– -5.76) |
| Lakshadweep | 31.3 (7.8–54.7) | 4.3 (1.4–7.3) | –33.51 (–42.81– -24.20) | 0.10 (0.03–0.16) | –29.50 (–40.11– -18.90) |
| Madhya Pradesh | 12.9 (12.5–13.3) | 9.9 (9.1–10.6) | –7.01 (–8.06– -5.95) | 0.52 (0.47–0.58) | –8.68 (–9.91– -7.46) |
| Maharashtra | 16.9 (15.8–18.0) | 10.6 (9.7–11.5) | –9.26 (–11.54– -6.99) | 0.49 (0.40–0.58) | –10.72 (–13.41– -8.03) |
| Manipur | 18.0 (16.1–19.9) | 15.3 (13.9–16.7) | –0.97 (–4.03–2.09) | 0.93 (0.73–1.14) | –2.55 (–5.82–0.72) |
| Meghalaya | 16.8 (14.9–18.7) | 13.7 (11.6–15.9) | –3.05 (–6.53–0.43) | 0.81 (0.62–1.01) | 0.21 (–3.87–4.30) |
| Mizoram | 12.2 (9.9–14.5) | 11.3 (9.5–13.1) | –2.12 (–6.73–2.49) | 0.86 (0.56–1.15) | –2.43 (–7.48–2.63) |
| Nagaland | 21.9 (19.8–23.9) | 17.5 (15.4–19.5) | –5.28 (–8.76– -1.80) | 0.75 (0.60–0.89) | –4.48 (–8.45– -0.52) |
| New Delhi | 13.9 (11.3–16.5) | 7.0 (5.5–8.4) | –7.54 (–11.87– -3.21) | 0.45 (0.27–0.63) | –8.10 (–13.01– -3.19) |
| Odisha | 14.1 (13.4–14.8) | 10.4 (9.3–11.5) | –7.54 (–11.87– -3.21) | 0.53 (0.46–0.60) | –10.44 (–12.45– -8.44) |
| Puducherry | 16.4 (12.7–20.1) | 7.9 (6.4–9.5) | –16.85 (–23.08– -10.63) | 0.28 (0.17–0.40) | –21.56 (–29.95– -13.17) |
| Punjab | 22.4 (21.0–23.7) | 12.1 (11.1–13.1) | –13.31 (–15.69– -10.93) | 0.45 (0.38–0.52) | –15.77 (–18.62– -12.91) |
| Rajasthan | 10.1 (9.6–10.5) | 7.8 (7.1–8.5) | –4.40 (–5.58– -3.22) | 0.64 (0.57–0.72) | –5.44 (–6.86– -4.02) |
| Sikkim | 29.5 (26.0–33.0) | 14.2 (11.6–16.8) | –18.10 (–22.83– -13.38) | 0.41 (0.31–0.51) | –20.89 (–26.52– -15.27) |
| Tamil Nadu | 17.0 (16.0–18.1) | 9.0 (8.3–9.7) | –9.65 (–11.41– -7.88) | 0.48 (0.41–0.54) | –11.67 (–13.84– -9.51) |
| Tripura | 13.8 (11.1–16.4) | 18.5 (14.7–22.3) | –2.38 (–7.44–2.68) | 0.86 (0.60–1.13) | –5.14 (–10.48–0.20) |
| Uttar Pradesh | 11.9 (11.6–12.3) | 7.7 (7.3–8.1) | –7.01 (–7.84– -6.17) | 0.50 (0.46–0.54) | –8.53 (–9.52– -7.54) |
| Uttarakhand | 17.2 (16.0–18.5) | 11.1 (10.0–12.1) | –10.13 (–13.47– -6.79) | 0.47 (0.35–0.59) | –11.99 (–15.25– -8.73) |
| West Bengal | 17.0 (15.9–18.1) | 10.2 (8.7–11.8) | –10.43 (–12.78– -8.08) | 0.44 (0.36–0.53) | –13.16 (–15.86– -10.46) |
| Telangana | 18.8 (17.3–20.2) | 8.6 (6.9–10.4) | –13.87 (–17.70– -10.04) | 0.39 (0.28–0.49) | –17.96 (–22.74– -13.17) |

Note: No edu, No education; H. edu, Higher education; CI, confidence interval; SII, slope index of inequality; RII, relative index of inequality;

CIX, concentration index

**Supplementary Table 14. Education–based inequality in awareness of hypertension, India, 2016**

| **Categorization** | **Awareness of hypertension** | | **SII (95% CI)** | **RII (95% CI)** | **CIX*100 (95% CI)** |
| --- | --- | --- | --- | --- | --- |
|  | **No edu (95% CI)** | **H. edu (95% CI)** |  |  |  |
| **National level** | 27.7 (27.2–28.2) | 33.4 (32.4–34.3) | 6.93 (5.18–8.67) | 1.25 (1.18–1.32) | 4.51 (3.63–5.40) |
| **Place of residence** |  |  |  |  |  |
| Urban | 34.3 (33.1–35.4) | 35.8 (34.6–37.0) | 1.65 (–1.76–5.06) | 1.05 (0.95–1.15) | 2.95 (1.27–4.63) |
| Rural | 25.7 (25.1–26.3) | 29.0 (27.4–30.5) | 5.46 (3.64–7.29) | 1.22 (1.14–1.29) | 3.13 (2.18–4.08) |
| **Region** |  |  |  |  |  |
| North | 36.2 (36.2–36.2) | 31.8 (31.8–31.8) | –3.55 (–6.87– -0.22) | 0.91 (0.82–0.99) | –1.85 (–3.37– -0.32) |
| Central | 21.1 (21.1–21.1) | 30.2 (30.2–30.2) | 9.66 (7.07–12.26) | 1.49 (1.33–1.65) | 4.83 (3.53–6.14) |
| East | 26.9 (26.9–26.9) | 38.6 (38.6–38.6) | 12.66 (8.73–16.60) | 1.51 (1.31–1.70) | 6.06 (4.06–8.07) |
| Northeast | 25.1 (25.1–25.1) | 31.7 (31.7–31.7) | 8.50 (4.42–12.58) | 1.34 (1.15–1.53) | 3.18 (1.36–5.00) |
| West | 22.2 (22.2–22.2) | 25.7 (25.7–25.7) | 3.25 (–1.95–8.45) | 1.14 (0.90–1.39) | 1.18 (–1.50–3.85) |
| South | 34.5 (34.5–34.5) | 38.9 (38.9–38.9) | 4.77 (0.52–9.02) | 1.14 (1.01–1.27) | 1.87 (–0.20–3.94) |
| **State** |  |  |  |  |  |
| Andaman and Nicobar Islands | 26.0 (16.1–35.9) | 28.0 (15.4–40.6) | –11.06 (–33.65–11.52) | 0.75 (0.30–1.20) | –4.02 (–14.85–6.80) |
| Andhra Pradesh | 30.0 (26.2–33.8) | 33.1 (25.1–41.0) | 3.76 (–6.07–13.59) | 1.13 (0.78–1.47) | 2.06 (–2.84–6.97) |
| Arunachal Pradesh | 26.8 (24.3–29.3) | 22.4 (17.2–27.7) | –2.93 (–10.12–4.25) | 0.90 (0.66–1.14) | –1.10 (–4.48–2.28) |
| Assam | 22.9 (20.9–24.9) | 25.3 (20.7–29.8) | 6.24 (1.13–11.35) | 1.26 (1.02–1.50) | 2.16 (–0.20–4.51) |
| Bihar | 28.6 (26.6–30.5) | 30.9 (25.1–36.7) | 6.02 (–0.54–12.58) | 1.21 (0.96–1.47) | 2.94 (–0.18–6.07) |
| Chandigarh | 41.2 (16.7–65.6) | 40.9 (19.6–62.2) | –3.61 (–40.50–33.27) | 0.91 (0.08–1.75) | –0.08 (–18.90–18.74) |
| Chhattisgarh | 12.5 (10.7–14.4) | 31.5 (25.5–37.5) | 15.46 (9.25–21.67) | 2.47 (1.56–3.37) | 7.13 (4.06–10.20) |
| Dadra and Nagar Haveli | 17.2 (3.1–31.4) | 41.2 (16.8–65.6) | 13.99 (–15.72–43.70) | 1.68 (–0.21–3.56) | 5.41 (–11.13–21.95) |
| Daman and Diu | 11.8 (0.7–22.8) | 23.5 (2.6–44.5) | –0.47 (–39.43–38.49) | 0.98 (–0.34–2.31) | 1.15 (–14.75–17.04) |
| Goa | 8.0 (–2.9–18.9) | 24.1 (12.5–35.6) | 10.15 (–8.48–28.77) | 1.46 (0.46–2.46) | 1.15 (–14.75–17.04) |
| Gujarat | 18.0 (15.4–20.7) | 24.9 (20.3–29.4) | 5.46 (–2.21–13.13) | 1.30 (0.83–1.76) | 1.96 (–1.23–5.14) |
| Haryana | 44.4 (41.0–47.7) | 38.0 (33.6–42.4) | –13.82 (–22.80– -4.84) | 0.74 (0.59–0.89) | –5.61 (–9.12– -2.09) |
| Himachal Pradesh | 30.9 (25.2–36.7) | 24.1 (19.0–29.2) | –7.54 (–16.57–1.48) | 0.78 (0.55–1.01) | –4.94 (–9.40– -0.47) |
| Jammu & Kashmir | 51.7 (49.4–54.1) | 30.3 (25.7–34.8) | –19.47 (–26.35– -12.59) | 0.65 (0.54–0.75) | –8.76 (–12.26– -5.26) |
| Jharkhand | 15.7 (13.8–17.6) | 27.3 (21.2–33.3) | 13.63 (7.89–19.37) | 1.95 (1.40–2.50) | 7.81 (4.86–10.76) |
| Karnataka | 20.1 (17.7–22.4) | 24.5 (19.9–29.0) | 1.74 (–4.69–8.18) | 1.08 (0.79–1.36) | 0.98 (–2.82–4.78) |
| Kerala | 35.5 (18.3–52.6) | 37.4 (31.3–43.5) | –4.51 (–17.20–8.18) | 0.89 (0.59–1.18) | –1.22 (–6.90–4.47) |
| Lakshadweep | 20.0 (–19.5–59.5) | 25.0 (–7.3–57.3) | –3.22 (–36.18–29.74) | 0.90 (–0.04–1.85) | –1.97 (–15.79–11.85) |
| Madhya Pradesh | 18.6 (17.2–20.0) | 31.2 (27.6–34.9) | 15.03 (11.00–19.06) | 1.87 (1.56–2.18) | 7.25 (5.18–9.33) |
| Maharashtra | 20.5 (17.7–23.4) | 26.7 (22.6–30.7) | 15.03 (11.00–19.06) | 1.87 (1.56–2.18) | 1.27 (–2.62–5.15) |
| Manipur | 26.5 (21.5–31.6) | 30.3 (25.7–34.9) | 6.93 (–2.42–16.27) | 1.27 (0.86–1.67) | 2.01 (–2.14–6.17) |
| Meghalaya | 32.2 (26.4–38.1) | 53.3 (44.9–61.7) | 23.25 (13.08–33.42) | 1.75 (1.29–2.22) | 12.85 (7.63–18.08) |
| Mizoram | 20.6 (12.5–28.7) | 42.1 (33.9–50.4) | 17.70 (4.49–30.92) | 1.60 (1.04–2.16) | 9.00 (1.50–16.50) |
| Nagaland | 24.8 (20.2–29.4) | 27.5 (21.7–33.3) | 7.47 (–1.28–16.22) | 1.28 (0.91–1.66) | 5.12 (1.13–9.10) |
| New Delhi | 32.6 (23.1–42.1) | 41.0 (30.3–51.6) | 13.75 (–4.55–32.06) | 1.45 (0.74–2.15) | 2.77 (–7.00–12.54) |
| Odisha | 25.4 (23.1–27.7) | 43.2 (37.6–48.8) | 22.13 (15.78–28.49) | 1.84 (1.50–2.18) | 2.77 (–7.00–12.54) |
| Puducherry | 44.4 (32.0–56.8) | 47.3 (37.1–57.5) | 3.74 (–23.14–30.62) | 1.07 (0.54–1.61) | –3.46 (–16.83–9.90) |
| Punjab | 38.7 (35.3–42.1) | 31.1 (27.0–35.2) | –9.21 (–16.55– -1.88) | 0.78 (0.63–0.94) | –4.49 (–7.94– -1.03) |
| Rajasthan | 25.4 (23.4–27.5) | 26.9 (22.8–31.0) | 0.44 (–5.02–5.91) | 1.02 (0.81–1.22) | 0.73 (–2.00–3.47) |
| Sikkim | 43.5 (36.5–50.5) | 40.4 (30.7–50.1) | –8.79 (–20.69–3.11) | 0.81 (0.58–1.04) | –1.29 (–7.11–4.53) |
| Tamil Nadu | 45.2 (41.8–48.5) | 43.2 (39.1–47.2) | –2.69 (–10.45–5.06) | 0.94 (0.78–1.10) | –0.14 (–3.71–3.42) |
| Tripura | 29.5 (19.9–39.1) | 44.6 (33.2–56.0) | 8.10 (–5.47–21.68) | 1.26 (0.77–1.76) | 2.06 (–4.47–8.58) |
| Uttar Pradesh | 23.8 (22.5–25.1) | 27.4 (24.9–30.0) | 6.62 (2.92–10.31) | 1.29 (1.11–1.48) | 3.02 (1.19–4.85) |
| Uttarakhand | 31.6 (27.9–35.3) | 27.5 (23.1–32.0) | –6.45 (–15.14–2.25) | 0.81 (0.59–1.04) | –2.41 (–6.65–1.83) |
| West Bengal | 27.2 (24.0–30.3) | 43.5 (35.5–51.6) | 12.87 (4.93–20.80) | 1.51 (1.12–1.90) | 5.67 (1.56–9.79) |
| Telangana | 38.5 (34.3–42.7) | 43.7 (33.2–54.2) | 10.71 (–2.83–24.26) | 1.31 (0.87–1.75) | 5.05 (–2.10–12.20) |

Note: No edu, No education; H. edu, Higher education; CI, confidence interval; SII, slope index of inequality; RII, relative index of inequality;

CIX, concentration index

**Supplementary Table 15. Education–based inequality in treatment of hypertension, India, 2016**

| **Categorization** | **Treatment of hypertension** | | **SII (95% CI)** | **RII (95% CI)** | **CIX*100 (95% CI)** |
| --- | --- | --- | --- | --- | --- |
|  | **No edu (95% CI)** | **H. edu (95% CI)** |  |  |  |
| **National level** | 24.0 (23.5–24.5) | 29.7 (28.7–30.6) | 6.91 (5.26–8.55) | 1.29 (1.21–1.37) | 3.79 (2.85–4.74) |
| **Place of residence** |  |  |  |  |  |
| Urban | 27.1 (26.0–28.2) | 31.0 (29.8–32.1) | 6.05 (4.31–7.79) | 1.27 (1.18–1.36) | 1.08 (–0.69–2.85) |
| Rural | 23.0 (22.4–23.6) | 27.3 (25.8–28.8) | 6.06 (4.32–7.81) | 1.27 (1.18–1.36) | 3.16 (2.16–4.17) |
| **Region** |  |  |  |  |  |
| North | 29.2 (29.2–29.2) | 26.5 (26.5–26.5) | –2.83 (–5.94–0.27) | 0.91 (0.81–1.00) | –1.34 (–2.99–0.31) |
| Central | 17.8 (17.8–17.8) | 28.5 (28.5–28.5) | 12.73 (10.24–15.21) | 1.78 (1.58–1.98) | 7.57 (6.12–9.02) |
| East | 25.2 (25.2–25.2) | 31.0 (31.0–31.0) | 8.87 (4.91–12.83) | 1.37 (1.18–1.56) | 5.17 (2.98–7.37) |
| Northeast | 19.8 (19.8–19.8) | 24.9 (24.9–24.9) | 5.01 (1.29–8.72) | 1.25 (1.04–1.46) | 2.64 (0.70–4.59) |
| West | 20.8 (20.8–20.8) | 26.3 (26.3–26.3) | 6.16 (1.10–11.23) | 1.28 (1.02–1.54) | 4.13 (1.06–7.20) |
| South | 29.4 (29.4–29.4) | 34.4 (34.4–34.4) | 4.46 (0.45–8.48) | 1.16 (1.01–1.31) | 2.66 (0.36–4.95) |
| **State** |  |  |  |  |  |
| Andaman and Nicobar Islands | 27.3 (17.2–37.3) | 28.0 (15.4–40.6) | –8.97 (–33.21–15.27) | 0.78 (0.26–1.30) | –5.89 (–18.69–6.91) |
| Andhra Pradesh | 26.4 (22.7–30.1) | 27.9 (20.4–35.5) | 2.81 (–6.69–12.31) | 1.11 (0.72–1.51) | 3.12 (–2.23–8.46) |
| Arunachal Pradesh | 18.5 (16.3–20.7) | 13.9 (9.5–18.2) | –4.15 (–10.11–1.81) | 0.80 (0.55–1.06) | –2.02 (–5.29–1.25) |
| Assam | 17.6 (15.7–19.4) | 21.3 (17.0–25.6) | 3.98 (–0.72–8.68) | 1.21 (0.94–1.48) | 2.03 (–0.43–4.49) |
| Bihar | 32.0 (30.0–34.1) | 33.7 (27.8–39.7) | 4.33 (–2.27–10.93) | 1.13 (0.92–1.35) | 1.44 (–2.13–5.01) |
| Chandigarh | 5.9 (–5.8–17.6) | 27.3 (8.0–46.6) | 18.70 (–11.50–48.90) | 2.02 (–0.25–4.30) | 10.19 (–8.08–28.47) |
| Chhattisgarh | 11.1 (9.4–12.9) | 24.6 (19.0–30.1) | 17.10 (11.17–23.03) | 2.88 (1.81–3.96) | 9.06 (5.62–12.50) |
| Dadra and Nagar Haveli | 20.7 (5.5–35.9) | 41.2 (16.8–65.6) | 6.99 (–24.12–38.10) | 1.29 (–0.21–2.80) | 2.42 (–15.81–20.65) |
| Daman and Diu | 14.7 (2.5–26.9) | 29.4 (6.9–51.9) | 5.61 (–33.63–44.85) | 1.17 (–0.09–2.43) | 1.32 (–17.59–20.23) |
| Goa | 32.0 (13.3–50.7) | 31.5 (18.9–44.0) | 1.95 (–16.43–20.33) | 1.06 (0.45–1.68) | 5.94 (–6.22–18.11) |
| Gujarat | 17.8 (15.1–20.4) | 22.9 (18.5–27.3) | 6.34 (–0.68–13.37) | 1.35 (0.91–1.78) | 2.74 (–0.96–6.44) |
| Haryana | 35.5 (32.3–38.7) | 34.4 (30.1–38.7) | –8.98 (–17.73– -0.22) | 0.79 (0.60–0.97) | –6.26 (–10.20– -2.32) |
| Himachal Pradesh | 27.7 (22.1–33.3) | 20.1 (15.3–24.8) | –8.63 (–17.64–0.37) | 0.69 (0.42–0.96) | –3.34 (–8.00–1.32) |
| Jammu & Kashmir | 41.6 (39.4–43.9) | 26.9 (22.5–31.3) | –12.51 (–19.50– -5.51) | 0.72 (0.59–0.86) | –7.38 (–11.28– -3.47) |
| Jharkhand | 13.3 (11.5–15.1) | 19.6 (14.2–25.0) | 12.59 (7.32–17.85) | 2.02 (1.43–2.61) | 7.00 (3.85–10.15) |
| Karnataka | 20.3 (17.9–22.6) | 25.1 (20.5–29.7) | 2.66 (–4.34–9.67) | 1.12 (0.78–1.46) | 0.04 (–3.76–3.85) |
| Kerala | 32.3 (15.5–49.0) | 37.8 (31.7–43.9) | 1.96 (–11.49–15.41) | 1.06 (0.63–1.49) | 0.41 (–6.00–6.82) |
| Lakshadweep | 20.0 (–19.5–59.5) | 12.5 (–12.2–37.2) | 2.33 (–20.25–24.92) | 1.10 (0.05–2.15) | 1.88 (–11.64–15.40) |
| Madhya Pradesh | 18.7 (17.3–20.1) | 31.9 (28.2–35.6) | 18.04 (13.95–22.13) | 2.07 (1.72–2.41) | 9.80 (7.37–12.22) |
| Maharashtra | 21.7 (18.8–24.6) | 29.2 (25.1–33.3) | 5.42 (–1.45–12.29) | 1.23 (0.91–1.55) | 2.01 (–2.14–6.16) |
| Manipur | 9.9 (6.4–13.3) | 17.2 (13.4–21.0) | 8.04 (1.02–15.07) | 1.65 (0.94–2.37) | 3.87 (0.03–7.72) |
| Meghalaya | 29.8 (24.1–35.5) | 43.8 (35.5–52.1) | 19.57 (8.99–30.14) | 1.64 (1.18–2.11) | 10.62 (4.62–16.62) |
| Mizoram | 21.6 (13.4–29.9) | 26.4 (19.1–33.8) | 4.64 (–8.51–17.79) | 1.19 (0.62–1.76) | 4.60 (–3.59–12.79) |
| Nagaland | 11.7 (8.3–15.1) | 10.5 (6.5–14.5) | 1.41 (–4.77–7.58) | 1.11 (0.59–1.64) | –0.20 (–3.48–3.07) |
| New Delhi | 32.6 (23.1–42.1) | 37.3 (26.9–47.8) | 11.87 (–6.68–30.41) | 1.41 (0.66–2.17) | 5.14 (–6.13–16.41) |
| Odisha | 21.5 (19.3–23.7) | 36.6 (31.2–42.1) | 20.86 (14.67–27.06) | 1.93 (1.53–2.32) | 10.34 (6.85–13.82) |
| Puducherry | 22.2 (11.9–32.6) | 26.9 (17.8–36.0) | 1.12 (–22.43–24.67) | 1.03 (0.38–1.68) | 2.56 (–12.75–17.87) |
| Punjab | 24.2 (21.2–27.2) | 22.8 (19.1–26.6) | –4.16 (–10.18–1.87) | 0.83 (0.61–1.05) | –0.85 (–4.26–2.57) |
| Rajasthan | 22.5 (20.6–24.5) | 24.2 (20.2–28.2) | 2.33 (–3.09–7.75) | 1.10 (0.86–1.34) | 1.64 (–1.37–4.65) |
| Sikkim | 23.8 (17.8–29.9) | 29.3 (20.3–38.3) | –0.13 (–10.33–10.07) | 0.99 (0.58–1.41) | 1.33 (–4.58–7.24) |
| Tamil Nadu | 37.2 (33.9–40.4) | 38.8 (34.8–42.8) | 2.82 (–4.50–10.14) | 1.08 (0.87–1.29) | 1.52 (–2.45–5.49) |
| Tripura | 22.7 (13.9–31.5) | 35.1 (24.2–46.1) | 3.13 (–9.90–16.16) | 1.12 (0.61–1.62) | 0.09 (–6.79–6.97) |
| Uttar Pradesh | 18.4 (17.3–19.6) | 25.4 (22.9–27.9) | 10.45 (6.95–13.94) | 1.61 (1.36–1.87) | 5.69 (3.73–7.65) |
| Uttarakhand | 26.9 (23.4–30.5) | 20.0 (16.0–24.0) | –6.62 (–14.25–1.01) | 0.78 (0.57–1.00) | –2.62 (–7.14–1.89) |
| West Bengal | 20.5 (17.6–23.3) | 29.3 (21.9–36.6) | 9.88 (1.84–17.92) | 1.50 (1.00–1.99) | 6.29 (2.01–10.58) |
| Telangana | 34.5 (30.4–38.6) | 28.7 (19.2–38.3) | –2.32 (–14.57–9.93) | 0.93 (0.58–1.29) | 0.15 (–8.12–8.43) |

Note: No edu, No education; H. edu, Higher education; CI, confidence interval; SII, slope index of inequality; RII, relative index of inequality;

CIX, concentration index

**Supplementary Table 16. Education–based inequality in control of hypertension, India, 2016**

| **Categorization** | **Control of hypertension** | | **SII (95% CI)** | **RII (95% CI)** | **CIX*100 (95% CI)** |
| --- | --- | --- | --- | --- | --- |
|  | **No edu (95% CI)** | **H. edu (95% CI)** |  |  |  |
| **National level** | 15.5 (15.0–15.9) | 22 (21.2–22.9) | 7.55 (6.08–9.02) | 1.51 (1.39–1.63) | 6.12 (4.89–7.36) |
| **Place of residence** |  |  |  |  |  |
| Urban | 15.3 (14.4–16.2) | 19.1 (18–20.1) | 6.24 (3.35–9.12) | 1.38 (1.18–1.59) | 5.57 (3.23–7.91) |
| Rural | 14.1 (13.7–14.6) | 19.2 (18–20.4) | 8.04 (6.50–9.59) | 1.57 (1.43–1.70) | 7.20 (5.89–8.51) |
| **Region** |  |  |  |  |  |
| North | 18.8 (17.6–20.1) | 18.7 (16.8–20.6) | 0.12 (–2.64–2.88) | 1.01 (0.86–1.15) | 0.73 (–1.41–2.88) |
| Central | 12.7 (11.9–13.5) | 22.3 (20.5–24.2) | 11.45 (9.14–13.76) | 2.01 (1.73–2.30) | 9.85 (7.92–11.78) |
| East | 16.6 (15.7–17.4) | 21.3 (19.0–23.7) | 7.68 (4.39–10.96) | 1.49 (1.24–1.75) | 6.83 (4.08–9.58) |
| Northeast | 8.2 (6.6–9.8) | 16.0 (12.3–19.8) | 7.90 (4.99–10.81) | 1.93 (1.46–2.41) | 6.08 (3.87–8.30) |
| West | 13.3 (12.0–14.6) | 19.7 (17.8–21.6) | 7.35 (2.75–11.94) | 1.51 (1.12–1.90) | 8.56 (4.39–12.72) |
| South | 18.0 (17.1–19.0) | 26.2 (24.6–27.9) | 8.14 (4.36–11.91) | 1.48 (1.22–1.75) | 5.51 (2.41–8.62) |
| **State** |  |  |  |  |  |
| Andaman and Nicobar Islands | 11.7 (4.4–18.9) | 14 (4.3–23.7) | 11.12 (–12.78–35.02) | 1.85 (–0.55–4.24) | 5.15 (–10.38–20.69) |
| Andhra Pradesh | 16.0 (12.9–19.0) | 22.1 (15.1–29.1) | 6.08 (–1.75–13.91) | 1.41 (0.78–2.03) | 6.23 (–0.75–13.20) |
| Arunachal Pradesh | 8.3 (6.8–9.9) | 6.9 (3.7–10.1) | 0.89 (–3.67–5.45) | 1.09 (0.61–1.57) | 1.08 (–2.65–4.81) |
| Assam | 6.8 (5.6–8.0) | 13.6 (10–17.2) | 7.17 (3.65–10.68) | 1.99 (1.31–2.66) | 5.77 (2.98–8.56) |
| Bihar | 24.8 (23.0–26.7) | 26.4 (20.9–31.9) | 5.21 (–1.08–11.49) | 1.21 (0.93–1.49) | 3.40 (–1.52–8.31) |
| Chandigarh | 5.9 (–5.8–17.6) | 22.7 (4.6–40.9) | 17.17 (–9.39–43.73) | 3.24 (–2.56–9.04) | 12.63 (–10.82–36.07) |
| Chhattisgarh | 6.3 (4.9–7.6) | 14.7 (10.1–19.2) | 13.21 (8.15–18.26) | 3.30 (1.83–4.78) | 9.88 (5.46–14.30) |
| Dadra and Nagar Haveli | 17.2 (3.1–31.4) | 23.5 (2.5–44.6) | –4.85 (–33.22–23.52) | 0.76 (–0.46–1.98) | 0.12 (–23.32–23.56) |
| Daman and Diu | 11.8 (0.7–22.8) | 29.4 (6.9–51.9) | 10.07 (–28.27–48.41) | 1.41 (–0.41–3.22) | 5.66 (–20.19–31.51) |
| Goa | 20.0 (3.9–36.1) | 25.9 (14.1–37.8) | 8.14 (–6.93–23.20) | 1.47 (0.42–2.52) | 6.15 (–9.59–21.88) |
| Gujarat | 12.7 (10.4–15.1) | 18.1 (14.1–22.1) | 7.26 (0.96–13.57) | 1.62 (0.97–2.27) | 5.68 (0.96–10.40) |
| Haryana | 26.6 (23.6–29.6) | 25.4 (21.5–29.4) | –8.15 (–15.94– -0.36) | 0.74 (0.53–0.95) | –8.25 (–13.54– -2.95) |
| Himachal Pradesh | 14.5 (10.1–18.8) | 15 (10.7–19.2) | 2.99 (–4.74–10.71) | 1.23 (0.56–1.90) | –1.57 (–7.21–4.06) |
| Jammu & Kashmir | 24.1 (22.1–26.1) | 19.5 (15.5–23.4) | –0.70 (–7.23–5.82) | 0.97 (0.72–1.22) | –0.74 (–5.77–4.30) |
| Jharkhand | 9.3 (7.8–10.9) | 14.4 (9.6–19.1) | 10.89 (6.25–15.54) | 2.22 (1.47–2.97) | 9.18 (4.99–13.37) |
| Karnataka | 12.4 (10.5–14.4) | 18.4 (14.3–22.5) | 8.02 (1.70–14.34) | 1.71 (1.01–2.41) | 7.55 (2.52–12.57) |
| Kerala | 22.6 (7.6–37.6) | 31.3 (25.5–37.1) | 12.42 (0.13–24.71) | 1.66 (0.84–2.49) | 10.00 (1.10–18.90) |
| Lakshadweep | 20.0 (–19.5–59.5) | 12.5 (–12.2–37.2) | 0.39 (–19.52–20.29) | 1.03 (–0.39–2.44) | –5.38 (–21.91–11.16) |
| Madhya Pradesh | 13.2 (12.0–14.4) | 23.6 (20.2–26.9) | 13.86 (10.13–17.59) | 2.18 (1.72–2.63) | 11.57 (8.35–14.78) |
| Maharashtra | 13.3 (10.9–15.7) | 21.1 (17.4–24.8) | 7.14 (0.79–13.50) | 1.45 (0.97–1.93) | 6.89 (1.27–12.52) |
| Manipur | 2.7 (0.9–4.6) | 7.8 (5.1–10.5) | 6.53 (1.53–11.52) | 2.12 (0.98–3.27) | 4.56 (0.66–8.46) |
| Meghalaya | 17.6 (12.8–22.3) | 30.7 (22.9–38.4) | 16.76 (6.73–26.79) | 1.80 (1.16–2.43) | 15.33 (7.06–23.61) |
| Mizoram | 9.3 (3.5–15.1) | 17.1 (10.9–23.4) | 10.03 (–2.31–22.37) | 1.77 (0.60–2.94) | 6.01 (–4.21–16.23) |
| Nagaland | 4.4 (2.2–6.5) | 4.8 (2–7.6) | 0.97 (–3.17–5.10) | 1.18 (0.34–2.02) | 0.15 (–3.05–3.35) |
| New Delhi | 22.1 (13.7–30.5) | 28.9 (19.1–38.8) | 8.98 (–7.35–25.32) | 1.47 (0.43–2.51) | 10.44 (–4.48–25.36) |
| Odisha | 14.8 (12.9–16.6) | 23.8 (19–28.6) | 18.60 (13.04–24.17) | 2.21 (1.67–2.75) | 15.35 (10.55–20.14) |
| Puducherry | 12.7 (4.4–21.0) | 20.4 (12.2–28.7) | 15.41 (–1.72–32.54) | 1.97 (0.56–3.38) | 18.15 (–1.29–37.58) |
| Punjab | 11.4 (9.2–13.6) | 13 (10–16) | –1.59 (–6.40–3.22) | 0.87 (0.50–1.24) | –1.00 (–4.94–2.94) |
| Rajasthan | 14.9 (13.2–16.5) | 19.5 (15.8–23.2) | 6.49 (1.72–11.26) | 1.44 (1.05–1.84) | 5.13 (1.08–9.17) |
| Sikkim | 7.3 (3.6–10.9) | 15.2 (8–22.3) | 7.38 (–0.42–15.19) | 2.00 (0.55–3.44) | 4.22 (–1.90–10.33) |
| Tamil Nadu | 23.1 (20.2–25.9) | 27.9 (24.2–31.6) | 5.46 (–1.51–12.42) | 1.25 (0.90–1.59) | 4.79 (–0.64–10.22) |
| Tripura | 15.9 (8.2–23.6) | 20.3 (11–29.5) | –0.43 (–11.16–10.30) | 0.98 (0.41–1.55) | 0.05 (–8.62–8.73) |
| Uttar Pradesh | 13.1 (12.1–14.1) | 19.8 (17.5–22) | 10.02 (6.82–13.22) | 1.83 (1.48–2.18) | 7.81 (5.16–10.46) |
| Uttarakhand | 15.9 (13.0–18.8) | 12.7 (9.4–16.1) | –1.36 (–7.94–5.22) | 0.93 (0.59–1.27) | –1.92 (–7.43–3.59) |
| West Bengal | 8.4 (6.4–10.4) | 18.4 (12.1–24.7) | 10.04 (4.17–15.92) | 2.21 (1.21–3.21) | 8.37 (3.34–13.41) |
| Telangana | 21.6 (18.1–25.2) | 19.5 (11.2–27.9) | 1.46 (–10.33–13.25) | 1.07 (0.48–1.67) | 0.99 (–10.08–12.07) |

Note: No edu, No education; H. edu, Higher education; CI, confidence interval; SII, slope index of inequality; RII, relative index of inequality;

CIX, concentration index

**Supplementary Table 17. Education–based inequality in prevalence of hypertension, Nepal, 2016**

| **Categorization** | **Prevalence of hypertension** | | **SII (95% CI)** | **RII (95% CI)** | **CIX*100 (95% CI)** |
| --- | --- | --- | --- | --- | --- |
|  | **No edu (95% CI)** | **H.edu (95% CI)** |  |  |  |
| **National** | 24.6 (23.5–25.8) | 15.5 (13.9–17.1) | –15.13 (–18.53– -11.74) | 0.47 (0.39–0.55) | –11.79 (–14.05– -9.53) |
| **Place of residence** |  |  |  |  |  |
| Urban | 27.3 (25.7–29.0) | 15.8 (14.0–17.7) | –17.64 (–22.05– -13.22) | 0.43 (0.34–0.52) | –14.56 (–17.58– -11.55) |
| Rural | 21.7 (20.1–23.3) | 14.5 (11.2–17.9) | –13.74 (–18.60– -8.88) | 0.48 (0.35–0.60) | –10.63 (–13.83– -7.43) |
| **Region** |  |  |  |  |  |
| Himal | 19.8 (15.9–23.7) | 8.3 (3.1–13.4) | –12.68 (–22.29– -3.07) | 0.49 (0.22–0.75) | –9.58 (–16.82– -2.33) |
| Pahaad | 29.3 (27.3–31.2) | 16.9 (14.6–19.1) | –18.76 (–24.30– -13.23) | 0.44 (0.33–0.54) | –14.63 (–18.41– -10.85) |
| Terai | 22.1 (20.6–23.6) | 14.8 (12.4–17.3) | –14.26 (–18.71– -9.81) | 0.45 (0.34–0.56) | –10.28 (–13.15– -7.42) |
| **Province** |  |  |  |  |  |
| Province 1 | 26.5 (23.6–29.4) | 13.0 (9.3–16.7) | –21.20 (–28.43r– -13.98) | 0.33 (0.21–0.46) | –16.64 (–21.65– -11.64) |
| Province 2 | 17.4 (15.4–19.3) | 18.8 (13.8–23.7) | –5.84 (–12.52–0.85) | 0.69 (0.40–0.98) | –5.29 (–9.84– -0.74) |
| Province 3 | 29.5 (26.6–32.5) | 18.3 (15.4–21.2) | –16.28 (–24.62– -7.94) | 0.50 (0.33–0.67) | –12.05 (–18.42– -5.68) |
| Province 4 | 39.2 (34.9–43.5) | 13.6 (9.1–18.1) | –35.27 (–45.23– -25.30) | 0.25 (0.15–0.35) | –28.63 (–34.56– -22.70) |
| Province 5 | 29.2 (26.2–32.2) | 13.3 (9.0–17.5) | –23.77 (–31.19– -16.35) | 0.33 (0.22–0.43) | –16.08 (–21.26– -10.89) |
| Province 6 | 16.6 (12.3–20.9) | 17.4 (9.5–25.2) | –6.31 (–14.89–2.28) | 0.66 (0.29–1.04) | –4.09 (–9.38–1.20) |
| Province 7 | 15.9 (12.7–19.2) | 8.9 (4.5–13.4) | –10.53 (–18.01– -3.05) | 0.47 (0.24–0.70) | –8.86 (–13.55– -4.17) |

Note: No edu, No education; H. edu, Higher education; CI, confidence interval; SII, slope index of inequality; RII, relative index of inequality;

CIX, concentration index

**Supplementary Table 18. Education–based inequality in awareness of hypertension, Nepal, 2016**

| **Categorization** | **Awareness of hypertension** | | **SII (95% CI)** | **RII (95% CI)** | **CIX*100 (95% CI)** |
| --- | --- | --- | --- | --- | --- |
|  | **No edu (95% CI)** | **H.edu (95% CI)** |  |  |  |
| **National** | 41.9 (39.2–44.5) | 40.5 (35.0–46.0) | –5.39 (–12.87–2.09) | 0.87 (0.71–1.04) | –1.60 (–4.77–1.58) |
| **Place of residence** |  |  |  |  |  |
| Urban | 45.1 (41.6–48.6) | 43.9 (37.6–50.1) | –6.42 (–16.37–3.52) | 0.86 (0.66–1.06) | –1.64 (–5.64–2.36) |
| Rural | 37.4 (33.4–41.4) | 27.1 (16.0–38.3) | –9.61 (–22.23–3.02) | 0.76 (0.49–1.04) | –2.99 (–7.78–1.80) |
| **Region** |  |  |  |  |  |
| Himal | 36.5 (25.8–47.2) | 28.8 (–2.4–59.9) | –3.91 (–26.56 –18.75) | 0.90 (0.34–1.46) | 1.30 (–10.66–13.26) |
| Pahaad | 41.8 (37.8–45.7) | 37.2 (30.1–44.4) | –8.96 (–18.32 –0.40) | 0.79 (0.61–0.98) | –2.55 (–7.13–2.04) |
| Terai | 42.6 (38.8–46.4) | 46.1 (37.2–55.1) | –0.73 (–13.77 –12.30) | 0.98 (0.68–1.29) | 0.84 (–3.63–5.31) |
| **Province** |  |  |  |  |  |
| Province 1 | 46.9 (40.5–53.3) | 40.7 (25.5–55.8) | –13.91 (–32.22–4.40) | 0.73 (0.42–1.03) | –5.59 (–12.70–1.52) |
| Province 2 | 44.7 (38.6–50.8) | 43.0 (28.3–57.7) | –5.94 (–34.02–22.14) | 0.87 (0.30–1.44) | –3.17 (–10.70–4.35) |
| Province 3 | 45.8 (39.9–51.7) | 38.5 (30.0–47.0) | –7.09 (–23.67–9.50) | 0.85 (0.52–1.17) | –0.96 (–8.36–6.45) |
| Province 4 | 47.0 (39.9–54.0) | 36.9 (19.4–54.4) | –22.41 (–37.27– -7.54) | 0.57 (0.36–0.78) | –8.13 (–14.89– -1.38) |
| Province 5 | 34.5 (28.6–40.3) | 41.4 (24.2–58.7) | 2.95 (–12.17–18.06) | 1.09 (0.62–1.55) | 0.58 (–6.12–7.29) |
| Province 6 | 19.1 (7.7–30.5) | 44.5 (18.8–70.2) | 25.35 (13.29–37.40) | 2.71 (1.23–4.2) | 9.53 (1.25–17.80) |
| Province 7 | 28.5 (18.3–38.7) | 50.5 (23.1–77.9) | 13.07 (–8.27–34.41) | 1.54 (0.42–2.66) | 5.07 (–4.13–14.26) |

Note: No edu, No education; H. edu, Higher education; CI, confidence interval; SII, slope index of inequality; RII, relative index of inequality;

CIX, concentration index

**Supplementary Table 19. Education–based inequality in treatment of hypertension, Nepal, 2016**

| **Categorization** | **Treatment of hypertension** | | **SII (95% CI)** | **RII (95% CI)** | **CIX*100 (95% CI)** |
| --- | --- | --- | --- | --- | --- |
|  | **No edu (95% CI)** | **H.edu (95% CI)** |  |  |  |
| **National** | 21.3 (19.1–23.4) | 21.2 (16.6–25.8) | –2.38 (–9.06–4.31) | 0.89 (0.6–1.18) | –2.43 (–7.57–2.72) |
| **Place of residence** |  |  |  |  |  |
| Urban | 24.4 (21.4–27.5) | 22.9 (17.6–28.1) | –4.26 (–13.26–4.74) | 0.83 (0.51–1.15) | –4.20 (–10.97–2.58) |
| Rural | 16.9 (13.9–20.0) | 14.7 (5.8–23.5) | –4.99 (–14.73–4.75) | 0.73 (0.28–1.19) | –3.61 (–11.03–3.81) |
| **Region** |  |  |  |  |  |
| Himal | 11.2 (4.2–18.2) | 24.4 (–5.2–54.0) | 0.95 (–16.46–18.37) | 1.08 (–0.37–2.52) | 5.36 (–8.43–19.16) |
| Pahaad | 20.5 (17.3–23.7) | 17.5 (11.9–23.1) | –4.84 (–13.69–4.02) | 0.78 (0.42–1.14) | –5.53 (–12.64–1.58) |
| Terai | 23.2 (19.9–26.4) | 26.5 (18.5–34.4) | 1.29 (–9.57–12.14) | 1.06 (0.57–1.54) | 2.06 (–5.58–9.69) |
| **Province** |  |  |  |  |  |
| Province 1 | 25.0 (19.5–30.6) | 33.7 (19.1–48.2) | –4.54 (–22.38–13.29) | 0.82 (0.20–1.45) | –2.36 (–15.09–10.36) |
| Province 2 | 23.0 (17.8–28.1) | 24.3 (11.6–37) | 6.38 (–12.95–25.71) | 1.29 (0.30–2.29) | 4.63 (–8.66–17.91) |
| Province 3 | 24.1 (19.1–29.2) | 19.3 (12.5–26.2) | –4.85 (–20.23–10.52) | 0.82 (0.30–1.34) | –5.77 (–17.85–6.31) |
| Province 4 | 22.9 (17.0–28.9) | 13.8 (1.3–26.4) | –14.85 (–28.92– -0.78) | 0.45 (0.10–0.81) | –11.14 (–21.65– -0.62) |
| Province 5 | 16.7 (12.1–21.3) | 16.1 (3.2–29.0) | –3.36 (–17.05–10.33) | 0.81 (0.12–1.50) | –2.59 (–12.62–7.44) |
| Province 6 | 9.1 (0.7–17.4) | 10.3 (–5.4–26.1) | –2.33 (–15.69–11.02) | 0.75 (–0.45–1.95) | –1.16 (–11.32–9.01) |
| Province 7 | 12.3 (4.9–19.7) | 30.6 (5.3–55.8) | 9.35 (–8.92–27.62) | 1.92 (–0.56–4.39) | 9.64 (–4.62–23.89) |

Note: No edu, No education; H. edu, Higher education; CI, confidence interval; SII, slope index of inequality; RII, relative index of inequality;

CIX, concentration index

**Supplementary Table 20. Education–based inequality in control of hypertension, Nepal, 2016**

| **Categorization** | **Control of hypertension** | | **SII (95% CI)** | **RII (95% CI)** | **CIX*100 (95% CI)** |
| --- | --- | --- | --- | --- | --- |
|  | **No edu (95% CI)** | **H.edu (95% CI)** |  |  |  |
| **National** | 10.7 (9.0–12.3) | 12.8 (9.0–16.5) | 0.53 (–4.27–5.34) | 1.05 (0.60–1.50) | 2.29 (–6.00–10.58) |
| **Place of residence** |  |  |  |  |  |
| Urban | 11.6 (9.3–13.8) | 13.2 (8.9–17.4) | 1.68 (–4.95–8.31) | 1.15 (0.53–1.76) | 0.38 (–10.30–11.06) |
| Rural | 9.4 (7.0–11.8) | 11.1 (3.2–19.0) | –4.84 (–11.81–2.12) | 0.59 (0.12–1.05) | –4.78 (–16.06–6.51) |
| **Region** |  |  |  |  |  |
| Himal | 4.5 (–0.1–9.1) | 24.4 (–5.2–54.0) | 6.65 (–4.83–18.13) | 2.51 (–1.75–6.77) | 12.88 (–7.60–33.35) |
| Pahaad | 9.7 (7.3–12.0) | 10.4 (5.9–14.9) | 1.91 (–5.18–9.00) | 1.20 (0.40–2.00) | 1.70 (–10.38–13.79) |
| Terai | 12.3 (9.8–14.8) | 15.3 (8.8–21.8) | –1.64 (–9.07–5.80) | 0.87 (0.35–1.40) | –1.69 (–13.33–9.95) |
| **Province** |  |  |  |  |  |
| Province 1 | 14.3 (9.8–18.7) | 19.0 (6.9–31.1) | –3.53 (–16.87–9.80) | 0.77 (0.01–1.53) | –5.12 (–24.30–14.05) |
| Province 2 | 14.0 (9.7–18.2) | 13.2 (3.2–23.3) | –3.95 (–17.89–9.99) | 0.75 (0.00–1.51) | –8.54 (–30.83–13.74) |
| Province 3 | 14.7 (10.5–18.9) | 11.2 (5.7–16.7) | –2.35 (–14.42–9.72) | 0.85 (0.14–1.55) | –1.41 (–21.37–18.54) |
| Province 4 | 7.6 (3.9–11.4) | 8.9 (–1.4–19.2) | 1.70 (–8.47–11.87) | 1.22 (–0.20–2.64) | 1.93 (–13.79–17.65) |
| Province 5 | 3.7 (1.4–6.0) | 10.3 (–0.3–21.0) | 3.56 (–4.66–11.77) | 1.96 (–1.03–4.94) | 7.81 (–4.47–20.09) |
| Province 6 | 6 (–0.9–12.9) | 10.3 (–5.4–26.1) | 1.96 (–9.26–13.17) | 1.36 (–1.11–3.84) | 8.03 (–7.15–23.21) |
| Province 7 | 7.9 (1.8–14.0) | 23.7 (0.4–47.0) | 10.59 (–3.16–24.33) | 2.82 (–0.48–6.12) | 16.64 (–12.68–45.96) |

Note: No edu, No education; H. edu, Higher education; CI, confidence interval; SII, slope index of inequality; RII, relative index of inequality;

CIX, concentration index

**A. India**


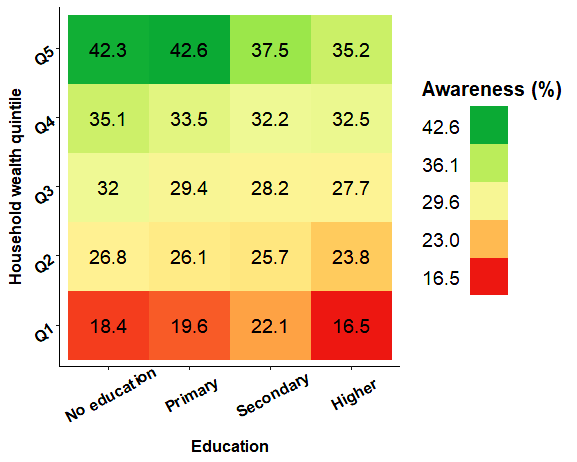

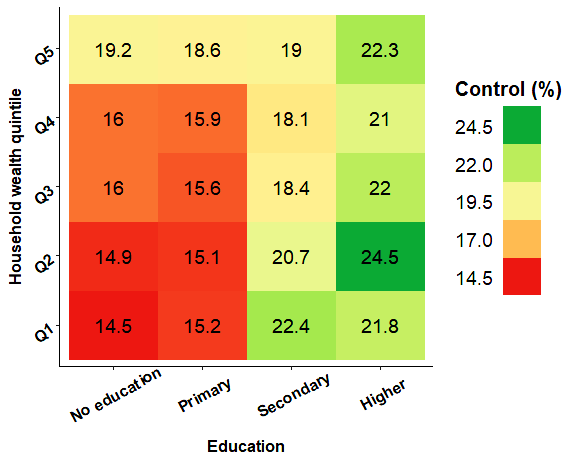


**B. Nepal**


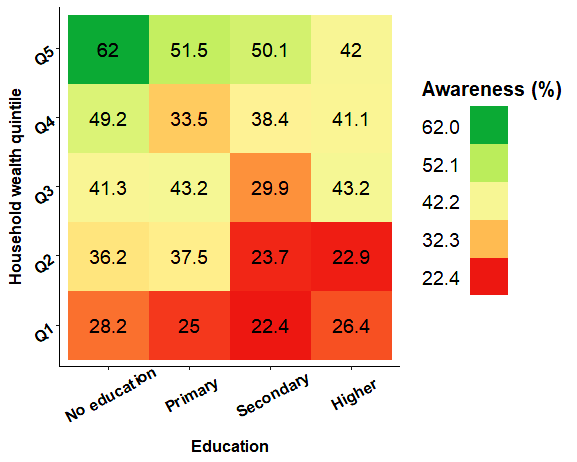

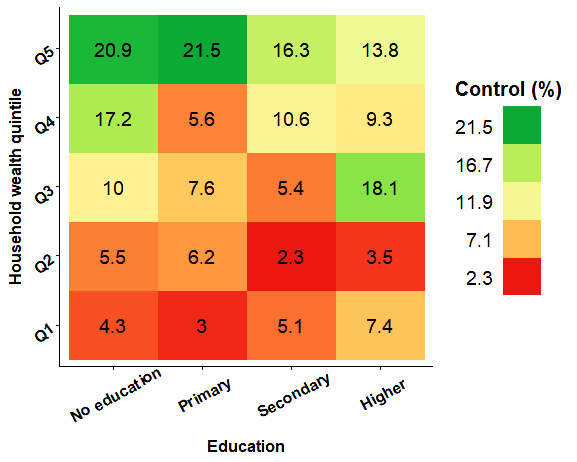
`

**Supplementary Figure 1. Awareness control of hypertension by household wealth quintile and education in India and Nepal, 2016**

***** Q1 = Poorest quintile, Q5 = Richest quintile * All the figures were generated using R programming software.

**A. India**


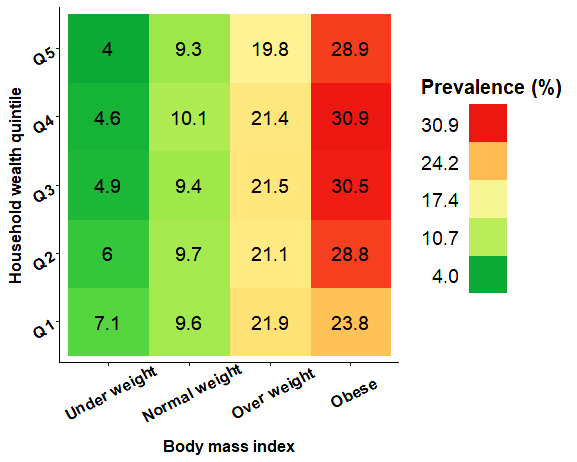

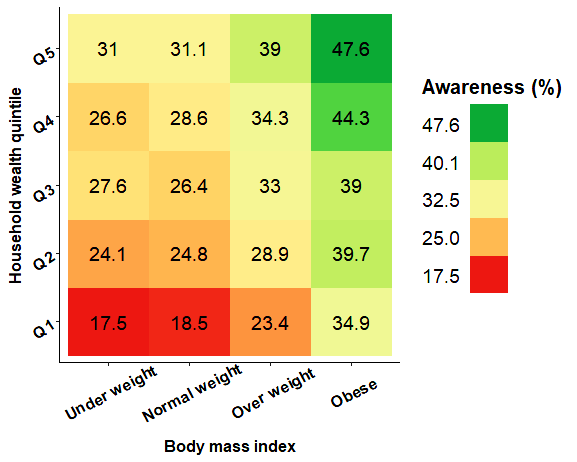


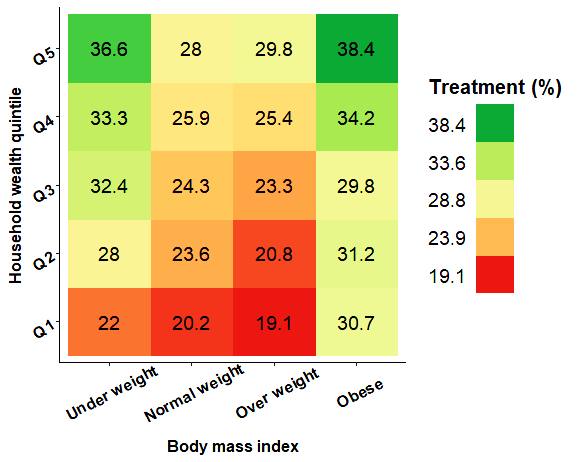

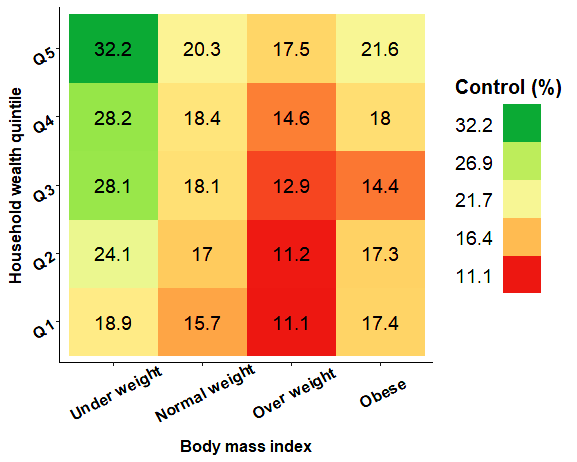


**B. Nepal**


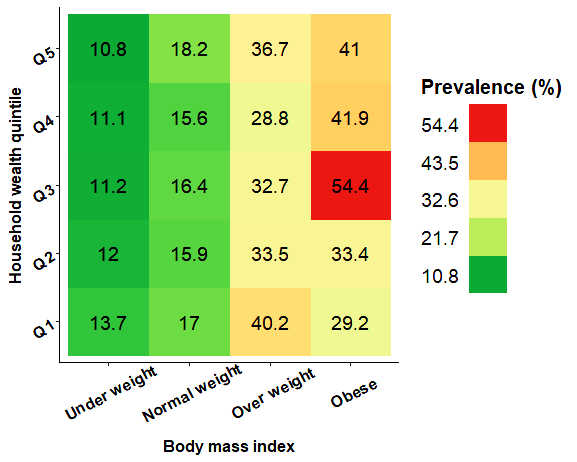

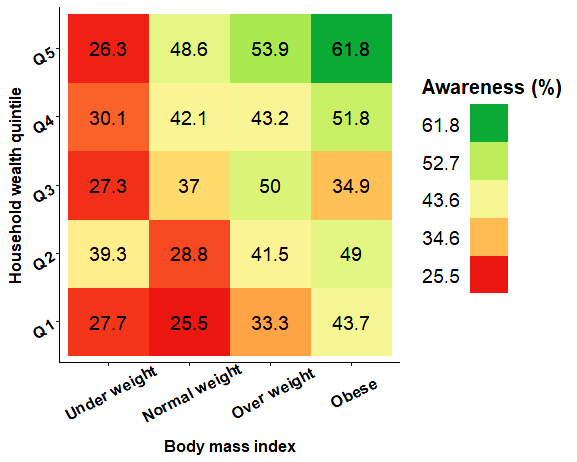


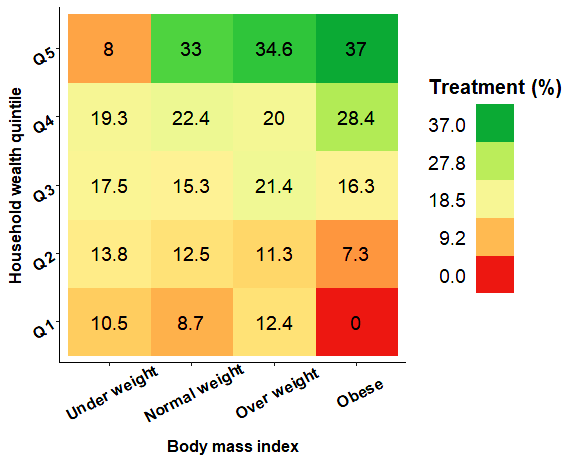

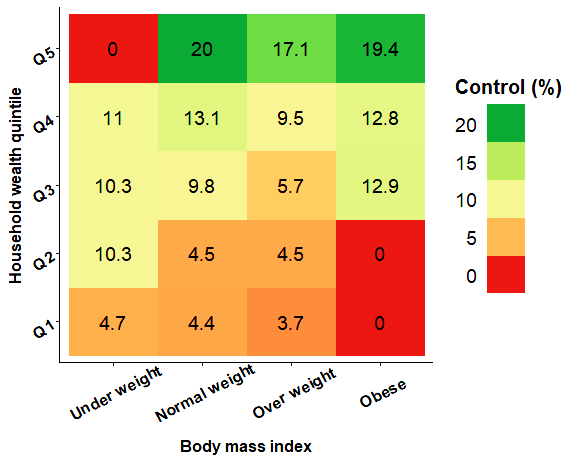


**Supplementary Figure 2. Prevalence, awareness, treatment and control of hypertension based on socio–economic status and body mass index in India and Nepal, 2016**

***** Q1 = Poorest quintile, Q5 = Richest quintile * All the figures were generated using R programming software.

**A. India**


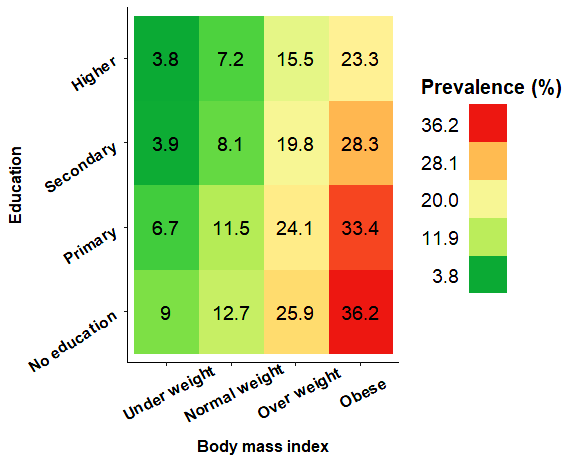

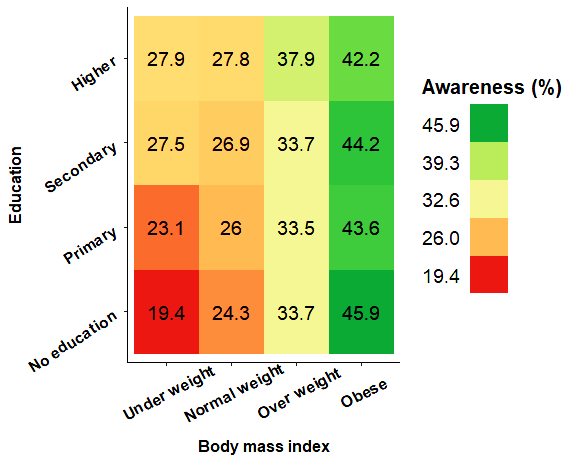


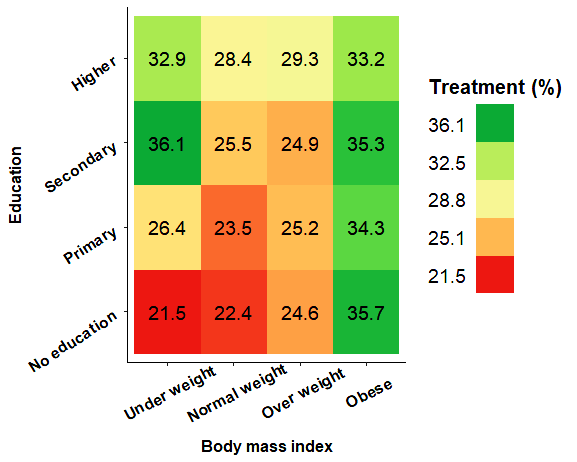

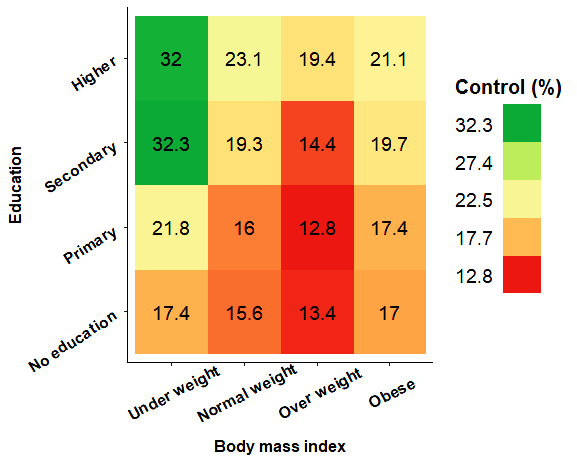
`

**B. Nepal**


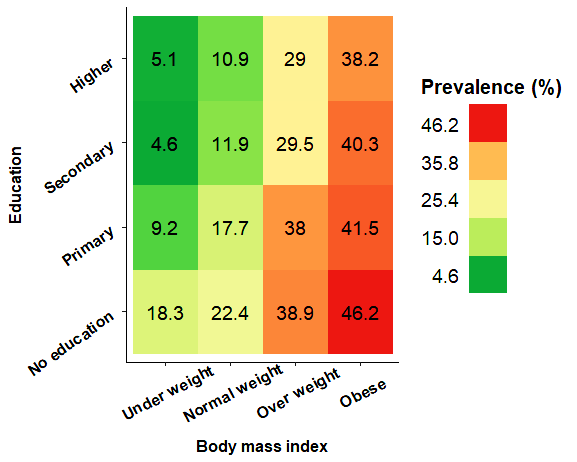

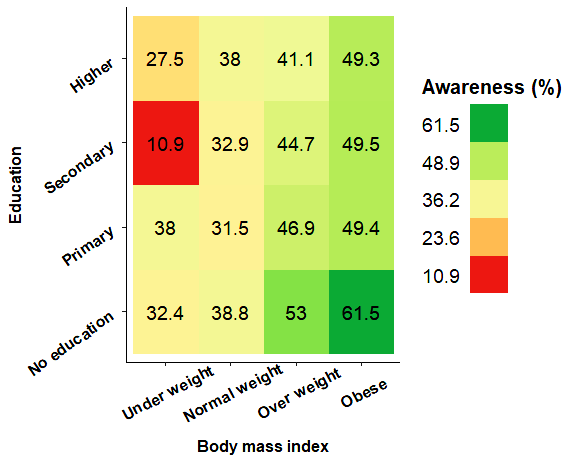


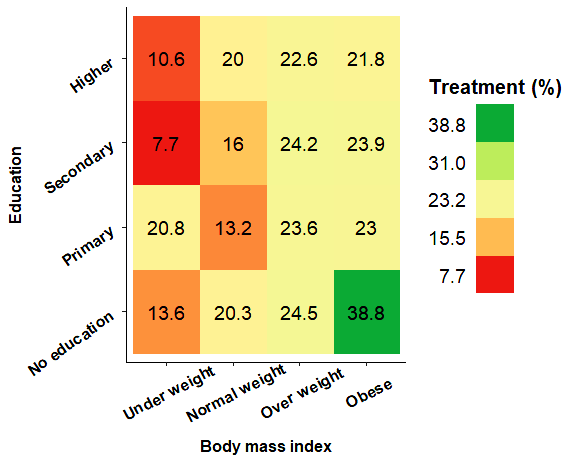

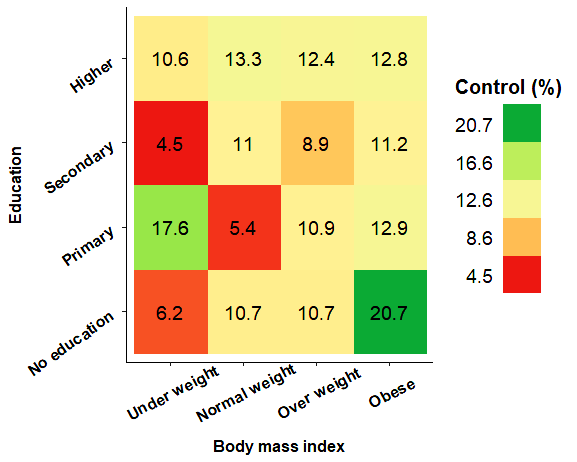


**Supplementary Figure 3. Prevalence, awareness, treatment and control of hypertension based on education-levels and body mass index in India and Nepal, 2016**

* All the figures were generated using R programming software.

**A. India**


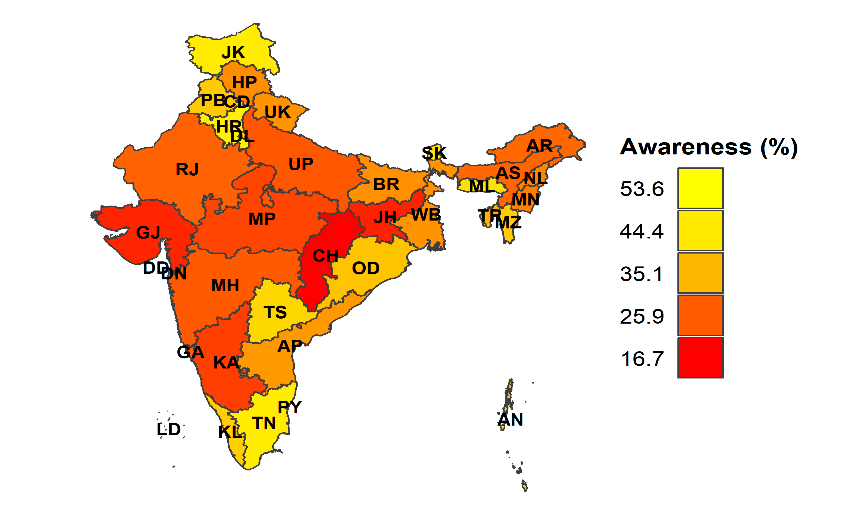

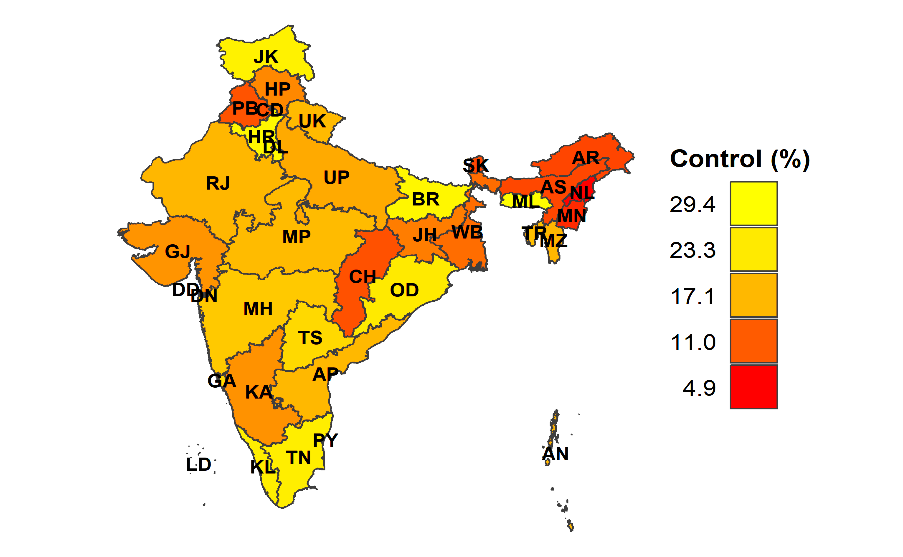


**B. Nepal**


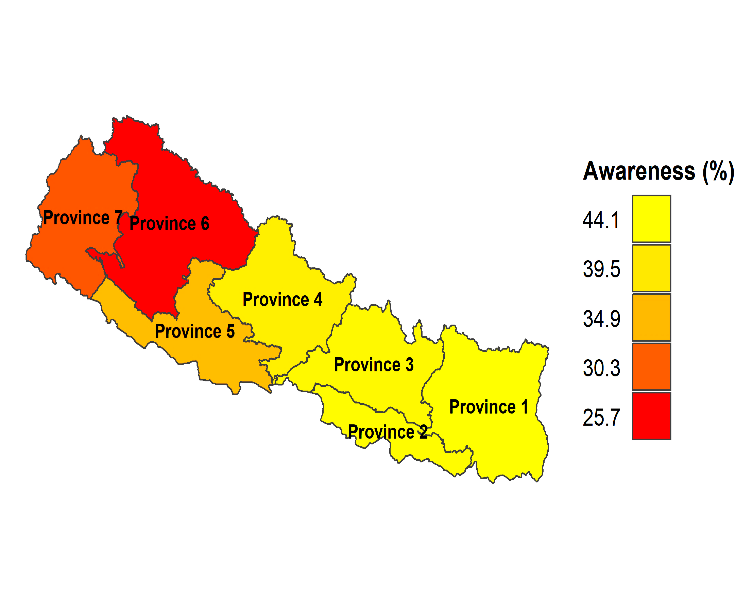

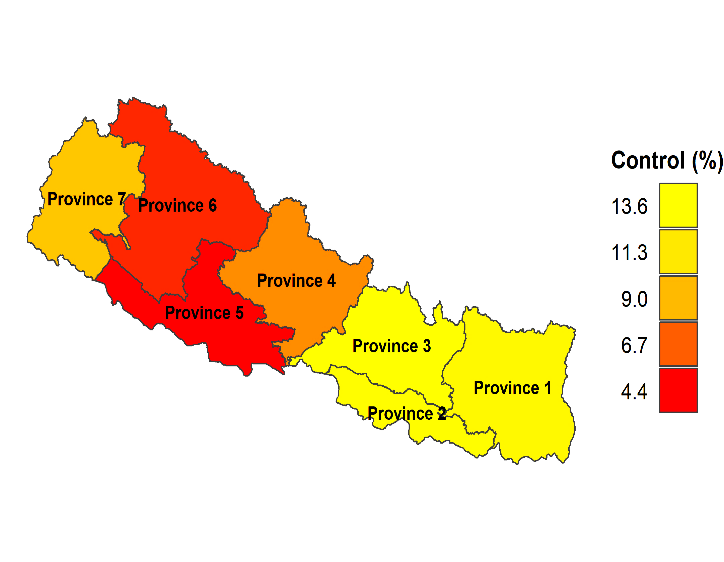


**Supplementary Figure 4. Awareness and control of hypertension at subnational levels in India and Nepal, 2016**

AN, Andaman and Nicobar Island; AP, Andhra Pradesh; AR, Arunachal Pradesh; AS, Assam; BR, Bihar; CD, Chandigarh; CH, Chhattisgarh; DN, Dadra and Nagar Haveli; DD, Daman and Diu; GA, Goa; GJ, Gujarat; HR, Haryana; HP, Himachal Pradesh; JK, Jammu & Kashmir; JH, Jharkhand; KA, Karnataka; KL, Kerala; LD, Lakshadweep; MP, Madhya Pradesh; MH, Maharashtra; MN, Manipur; ML, Meghalaya; MZ, Mizoram; NL, Nagaland; DL, New Delhi; OD, Odisha; PY, Puducherry; PB, Punjab; RJ, Rajasthan; SK, Sikkim; TN, Tamil Nadu; TR, Tripura; UP, Uttar Pradesh; UK, Uttarakhand; WB, West Bengal; TS, Telangana. * All the choropleth maps were generated in R programming software using spatial data from the DHS Spatial Data Repository. Permission to reproduce the map was obtained from DHS Program.

**A. India**

**Urban**


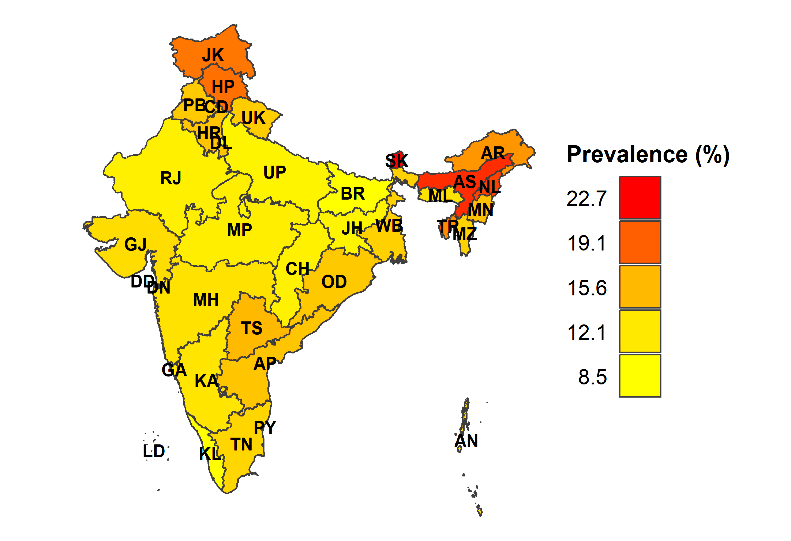

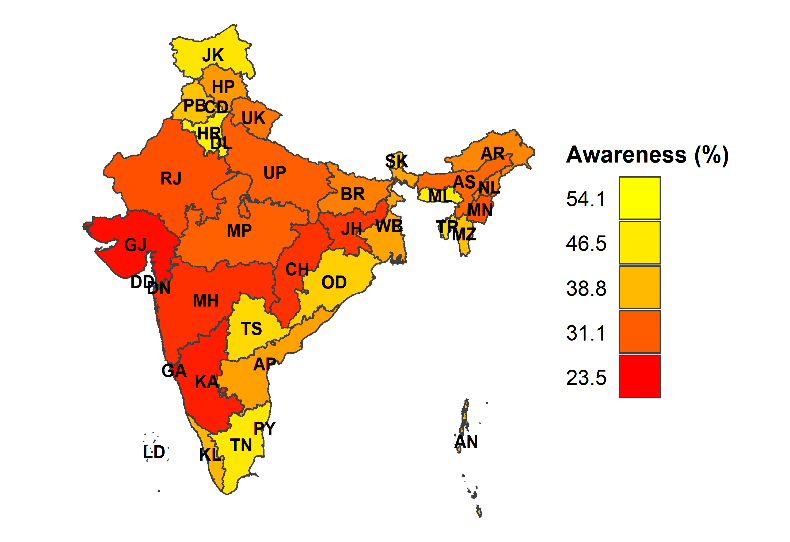

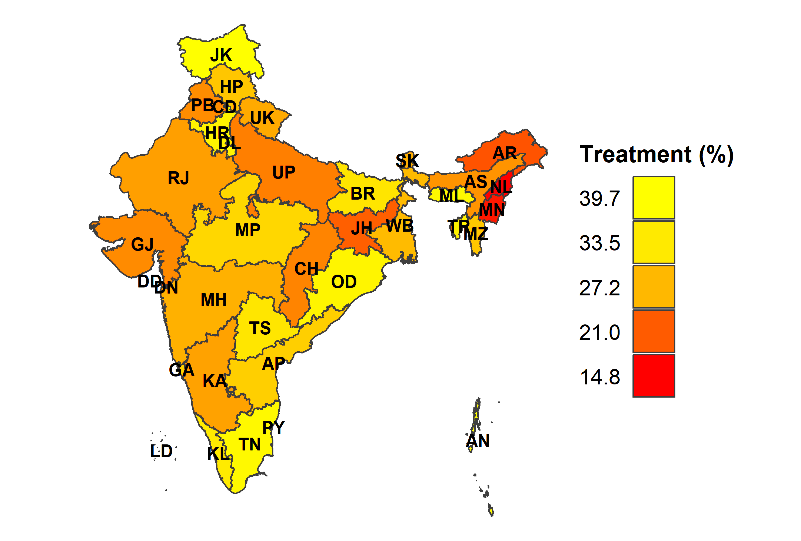

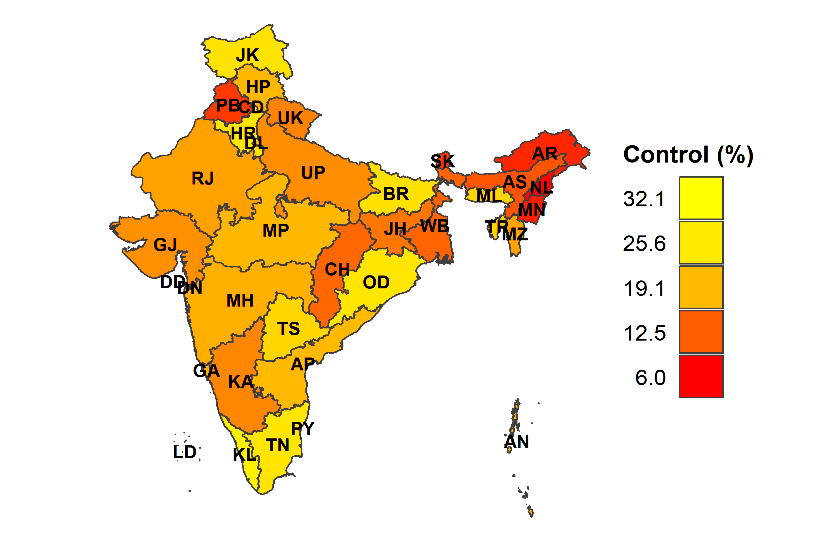


**B. Nepal**

**Urban**


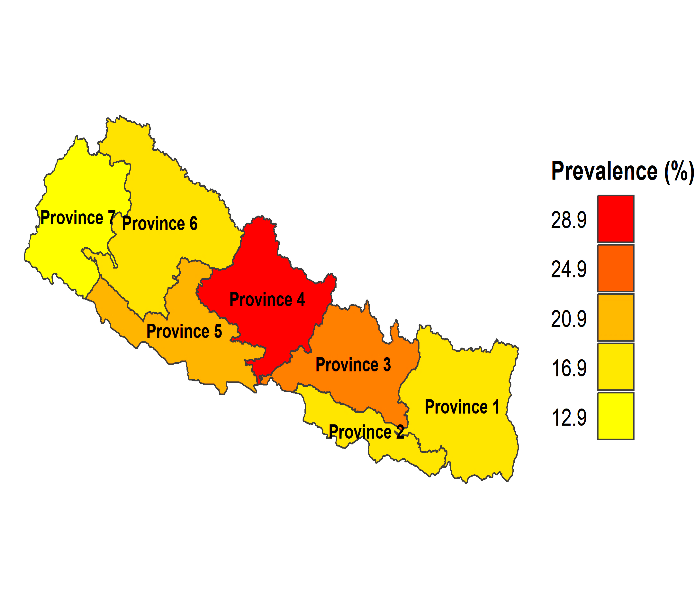

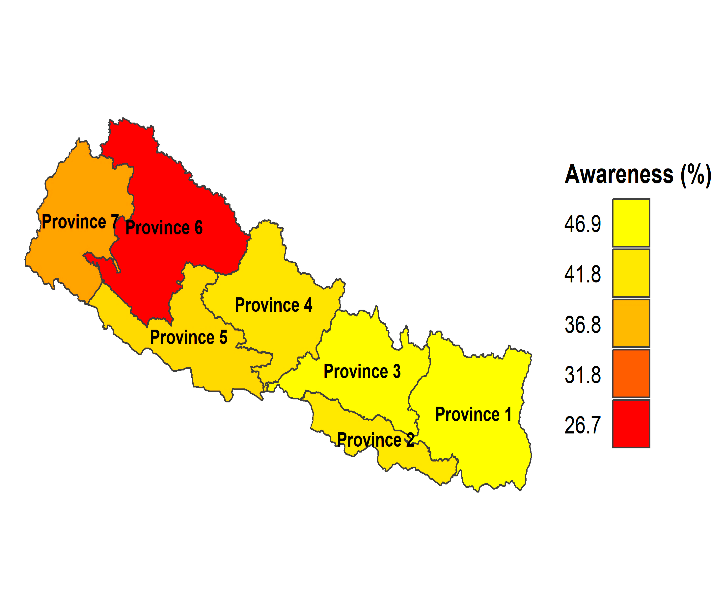


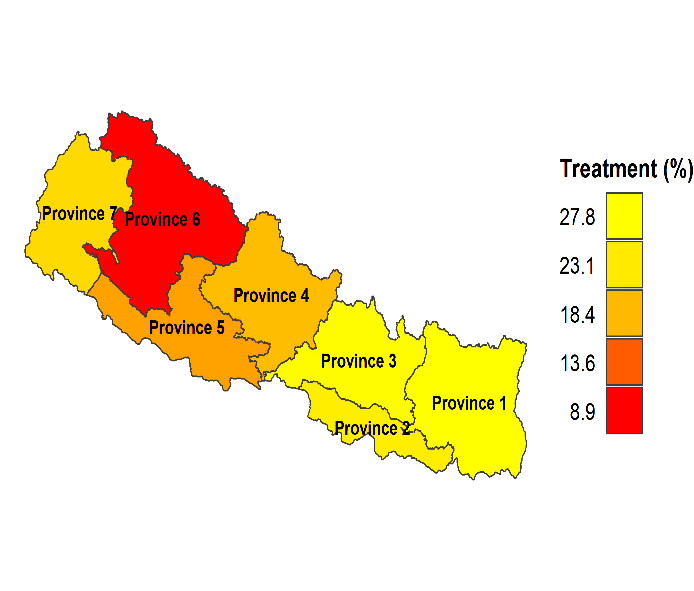

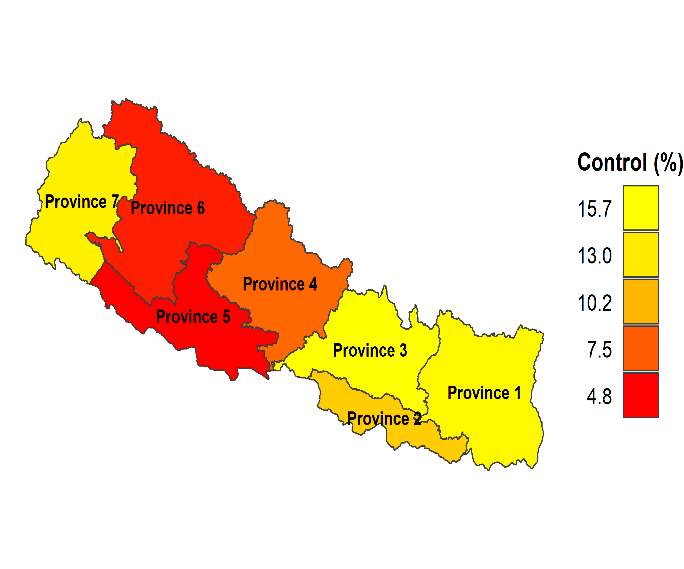


**Supplementary Figure 5. Prevalence and management of hypertension in urban residence in India and Nepal, 2016**

AN, Andaman and Nicobar Island; AP, Andhra Pradesh; AR, Arunachal Pradesh; AS, Assam; BR, Bihar; CD, Chandigarh; CH, Chhattisgarh; DN, Dadra and Nagar Haveli; DD, Daman and Diu; GA, Goa; GJ, Gujarat; HR, Haryana; HP, Himachal Pradesh; JK, Jammu & Kashmir; JH, Jharkhand; KA, Karnataka; KL, Kerala; LD, Lakshadweep; MP, Madhya Pradesh; MH, Maharashtra; MN, Manipur; ML, Meghalaya; MZ, Mizoram; NL, Nagaland; DL, New Delhi; OD, Odisha; PY, Puducherry; PB, Punjab; RJ, Rajasthan; SK, Sikkim; TN, Tamil Nadu; TR, Tripura; UP, Uttar Pradesh; UK, Uttarakhand; WB, West Bengal; TS, Telangana. * All the choropleth maps were generated in R programming software using spatial data from the DHS Spatial Data Repository. Permission to reproduce the map was obtained from DHS Program.

**A. India**

**Rural**

**
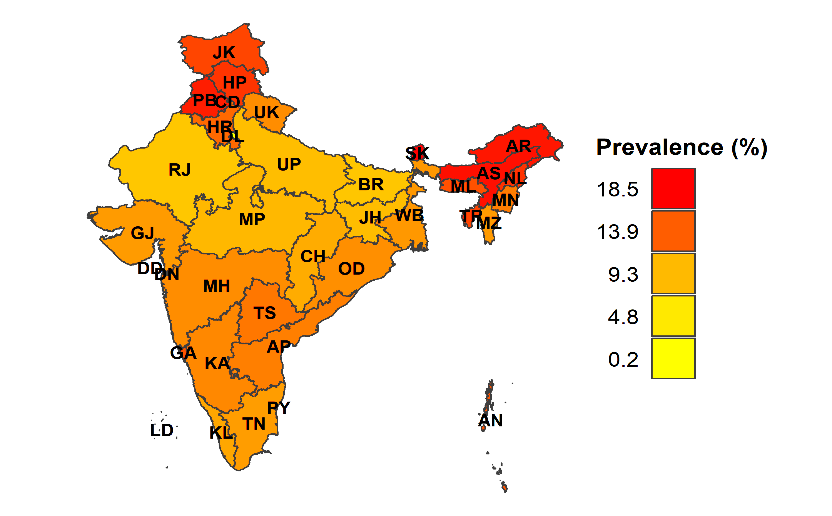

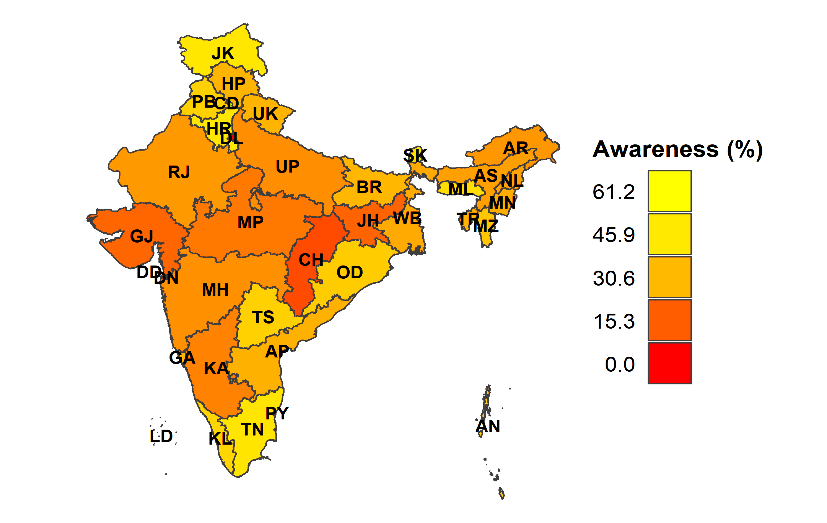

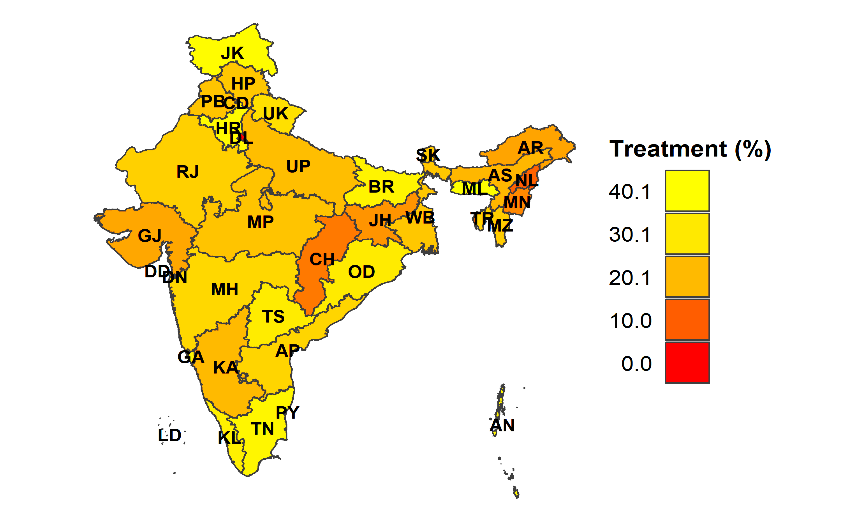

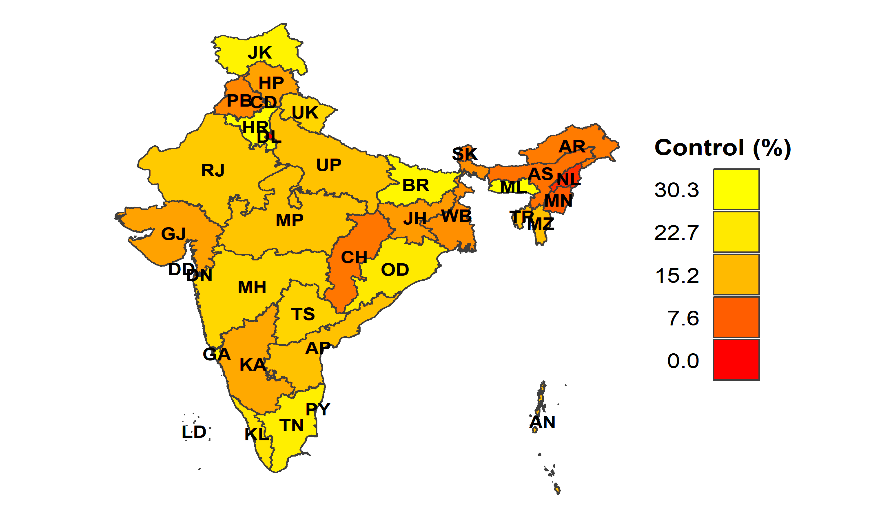
**

**B. Nepal**

**Rural**


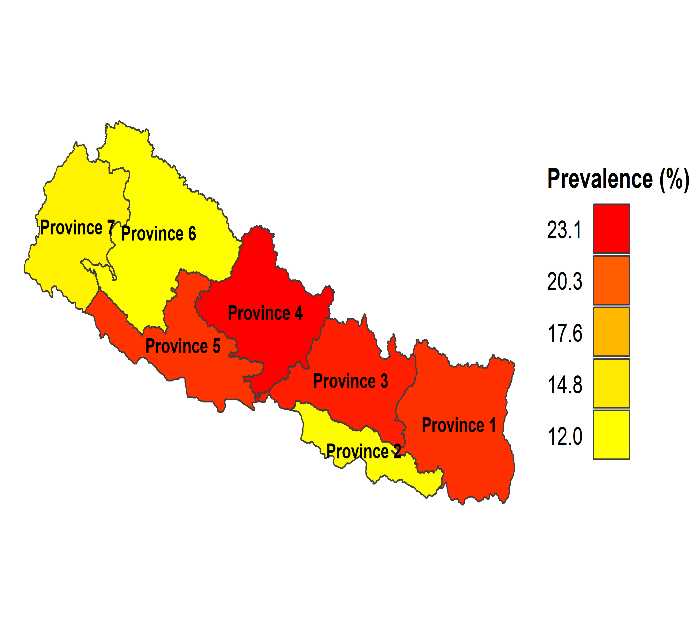

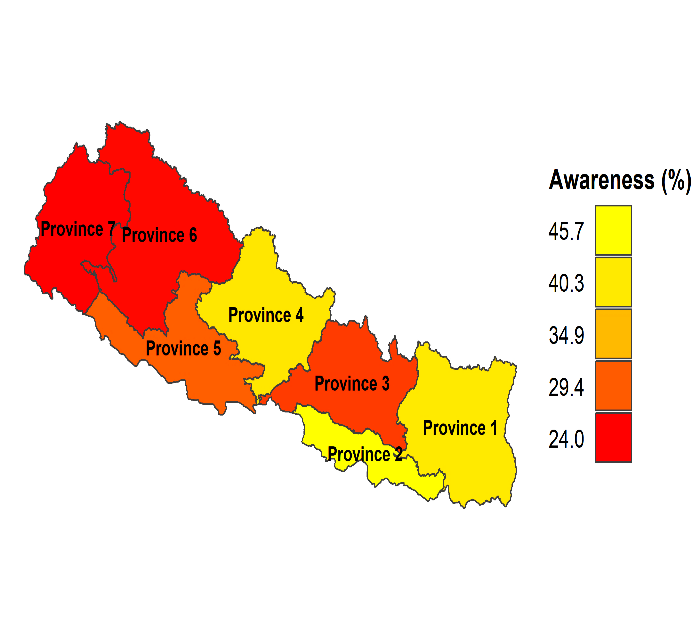

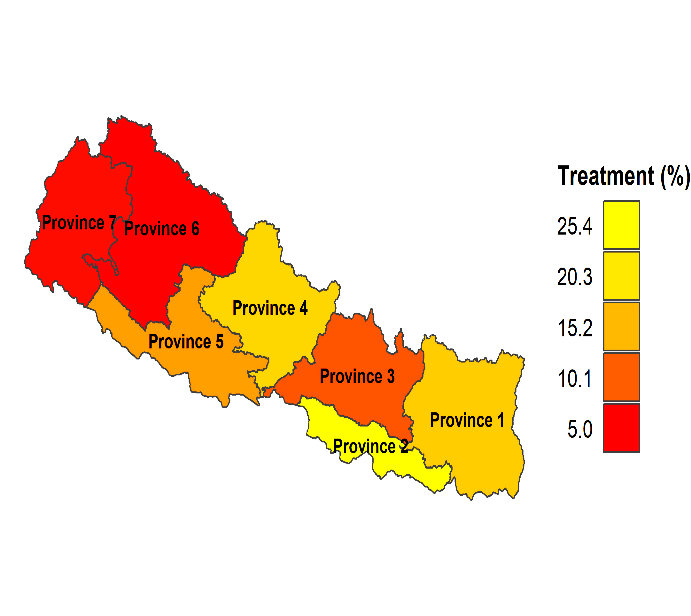

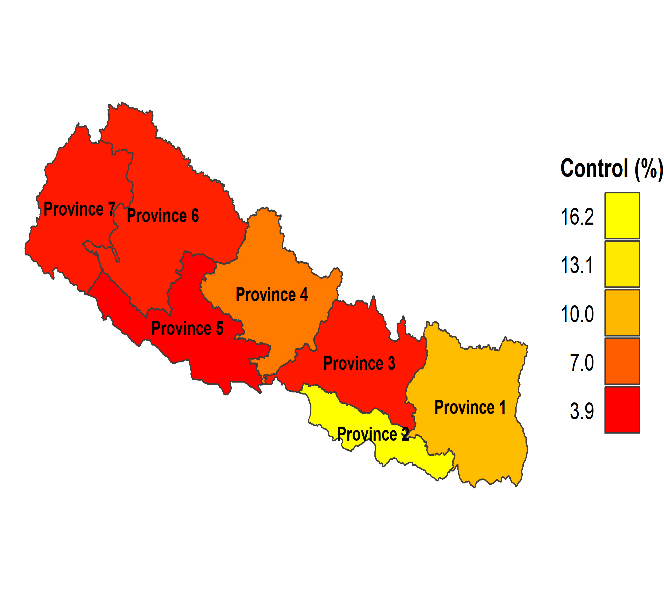


**Supplementary Figure 6. Prevalence and management of hypertension in rural residence in India and Nepal, 2016**

AN, Andaman and Nicobar Island; AP, Andhra Pradesh; AR, Arunachal Pradesh; AS, Assam; BR, Bihar; CD, Chandigarh; CH, Chhattisgarh; DN, Dadra and Nagar Haveli; DD, Daman and Diu; GA, Goa; GJ, Gujarat; HR, Haryana; HP, Himachal Pradesh; JK, Jammu & Kashmir; JH, Jharkhand; KA, Karnataka; KL, Kerala; LD, Lakshadweep; MP, Madhya Pradesh; MH, Maharashtra; MN, Manipur; ML, Meghalaya; MZ, Mizoram; NL, Nagaland; DL, New Delhi; OD, Odisha; PY, Puducherry; PB, Punjab; RJ, Rajasthan; SK, Sikkim; TN, Tamil Nadu; TR, Tripura; UP, Uttar Pradesh; UK, Uttarakhand; WB, West Bengal; TS, Telangana. * All the choropleth maps were generated in R programming software using spatial data from the DHS Spatial Data Repository. Permission to reproduce the map was obtained from DHS Program.

**A. India**

**Female**

**
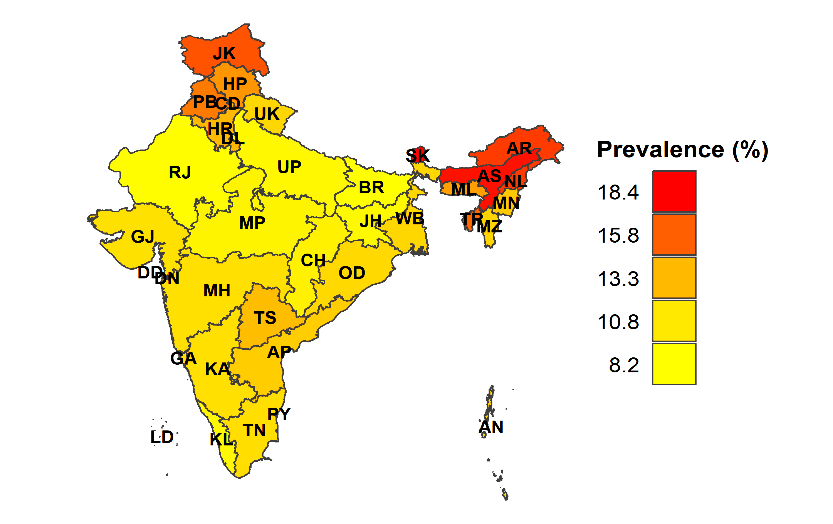

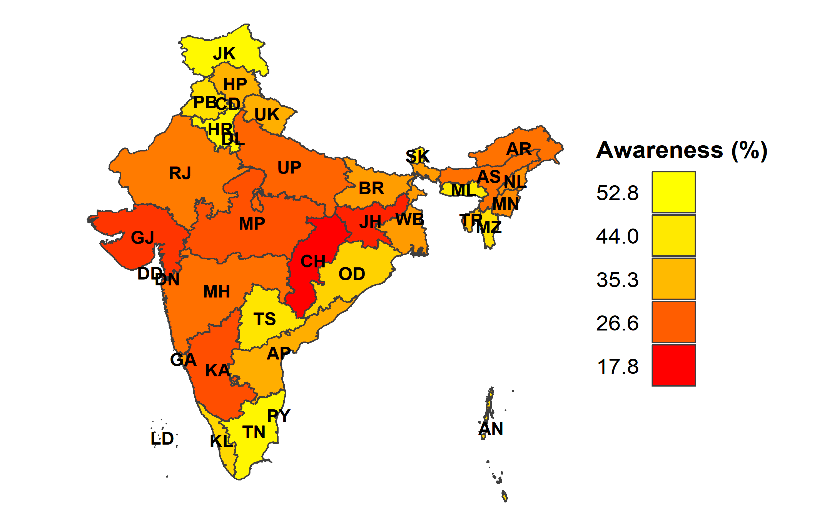

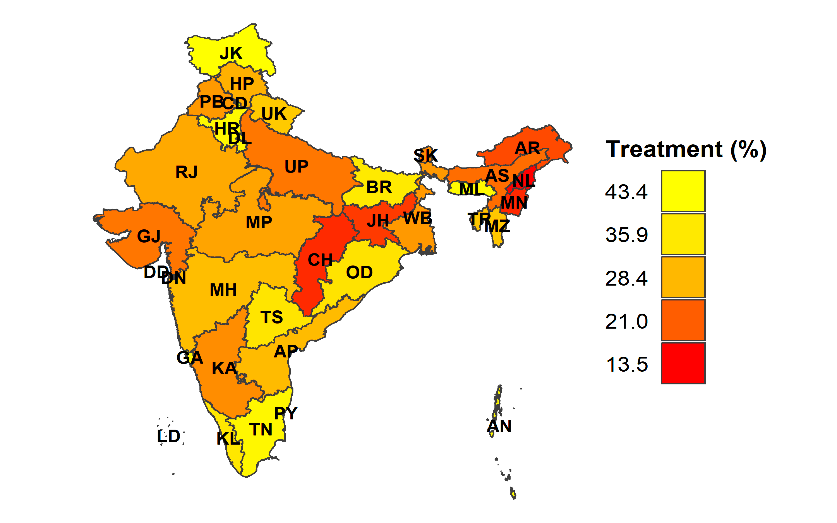

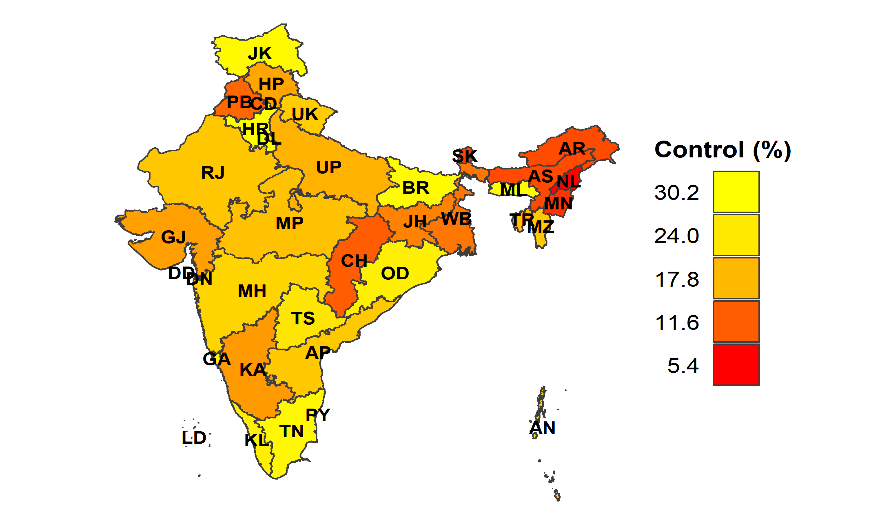
**

**B. Nepal**

**Female**


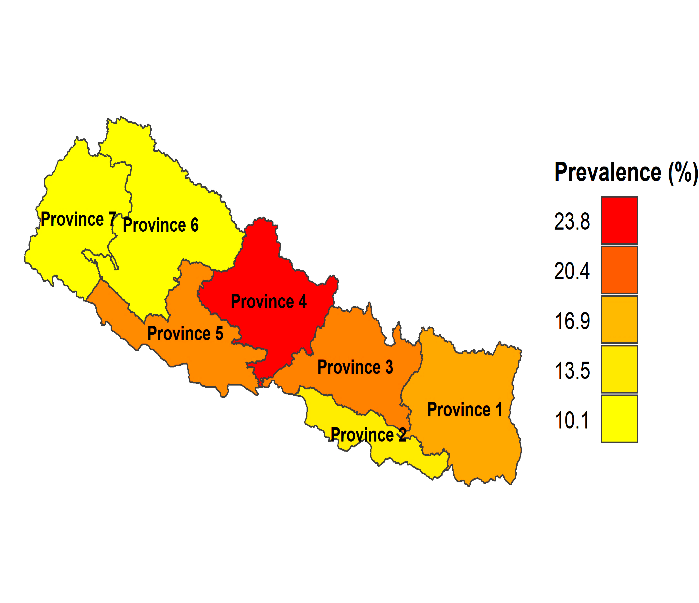

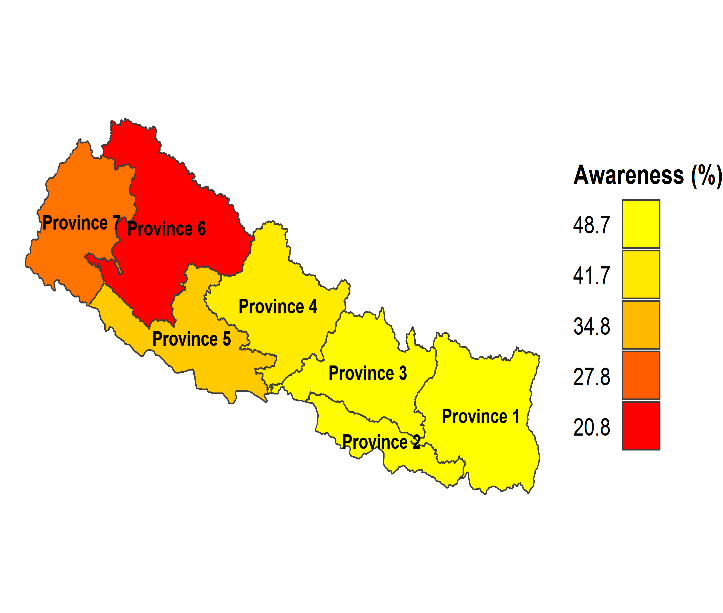

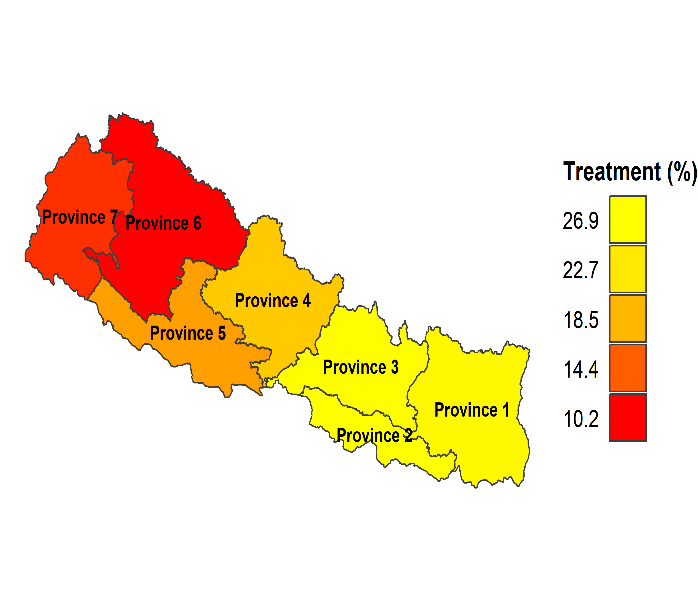

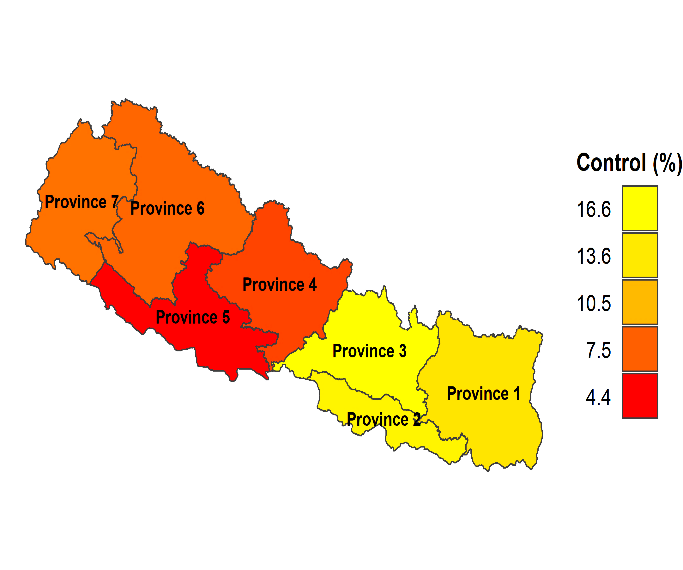


**Supplementary Figure 7. Prevalence and management of hypertenion in females in India and Nepal, 2016**

AN, Andaman and Nicobar Island; AP, Andhra Pradesh; AR, Arunachal Pradesh; AS, Assam; BR, Bihar; CD, Chandigarh; CH, Chhattisgarh; DN, Dadra and Nagar Haveli; DD, Daman and Diu; GA, Goa; GJ, Gujarat; HR, Haryana; HP, Himachal Pradesh; JK, Jammu & Kashmir; JH, Jharkhand; KA, Karnataka; KL, Kerala; LD, Lakshadweep; MP, Madhya Pradesh; MH, Maharashtra; MN, Manipur; ML, Meghalaya; MZ, Mizoram; NL, Nagaland; DL, New Delhi; OD, Odisha; PY, Puducherry; PB, Punjab; RJ, Rajasthan; SK, Sikkim; TN, Tamil Nadu; TR, Tripura; UP, Uttar Pradesh; UK, Uttarakhand; WB, West Bengal; TS, Telangana. * All the choropleth maps were generated in R programming software using spatial data from the DHS Spatial Data Repository. Permission to reproduce the map was obtained from DHS Program.

**A. India**

**Male**


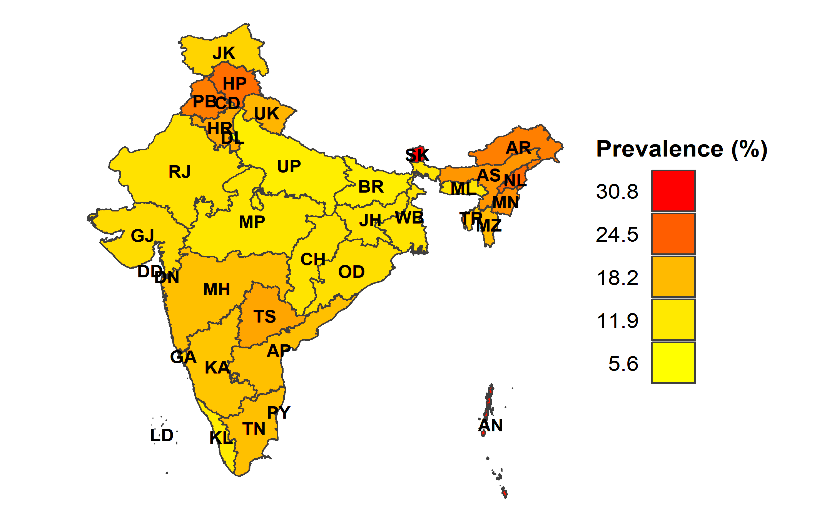

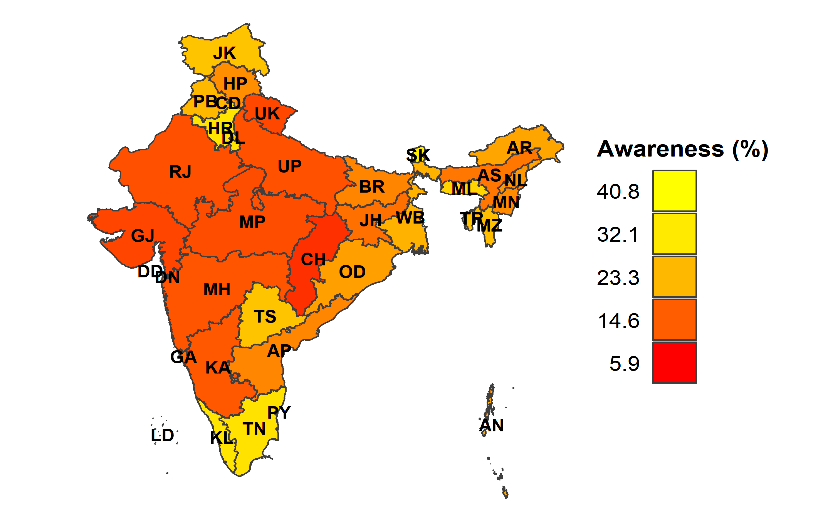


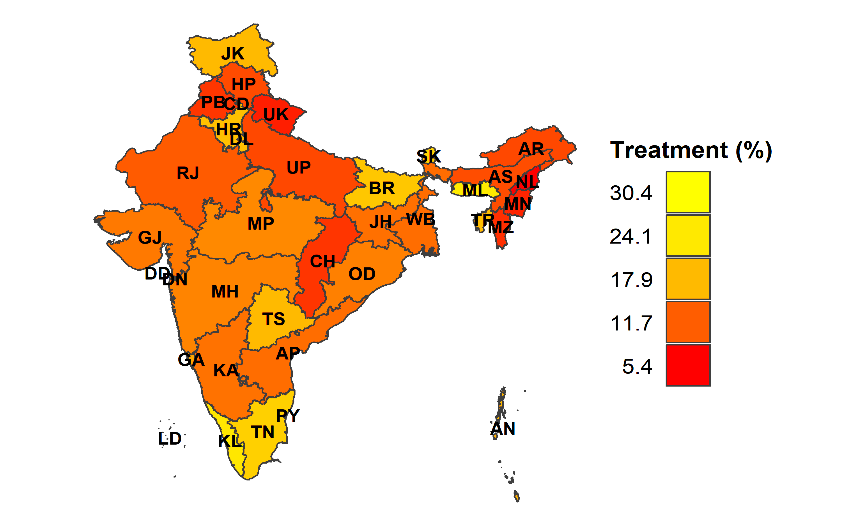

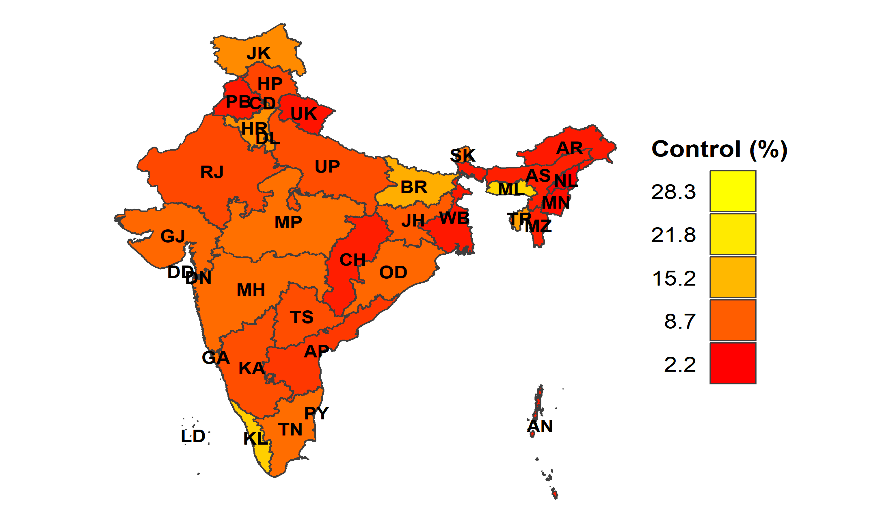


**B. Nepal**

**Male**


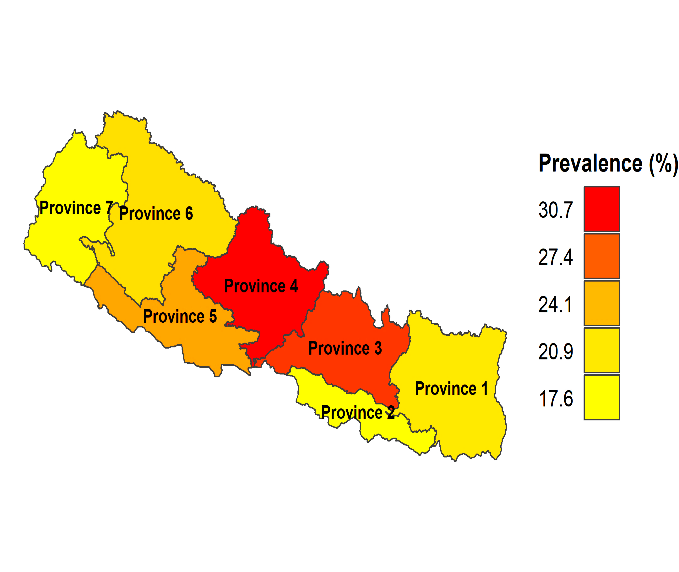

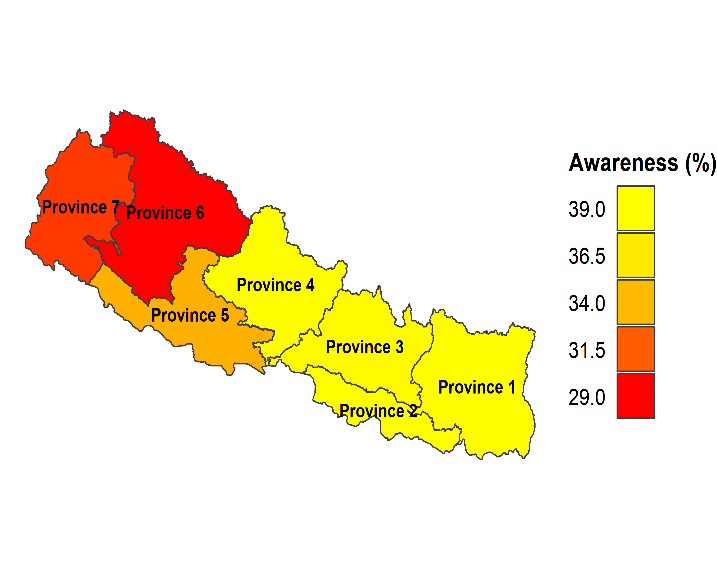

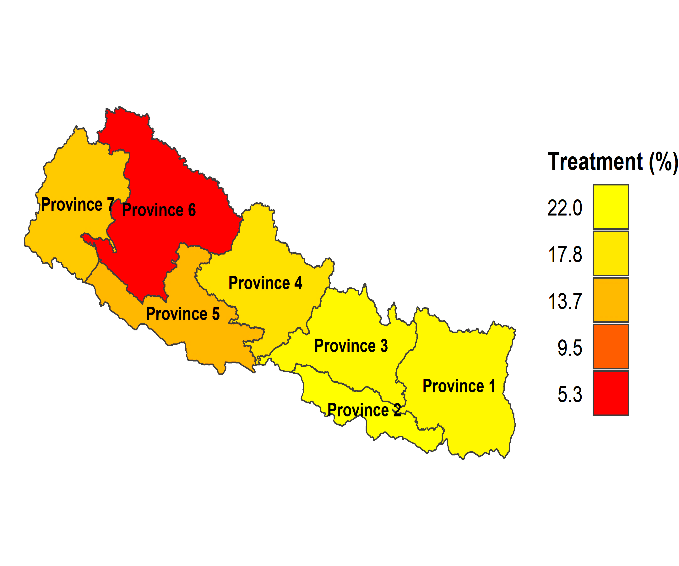

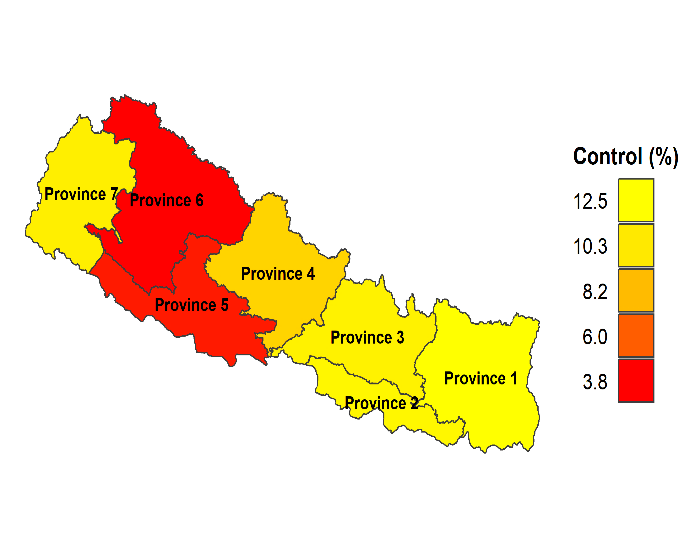


**Supplementary Figure 8. Prevalence and management of hypertenion in males in India and Nepal, 2016**

AN, Andaman and Nicobar Island; AP, Andhra Pradesh; AR, Arunachal Pradesh; AS, Assam; BR, Bihar; CD, Chandigarh; CH, Chhattisgarh; DN, Dadra and Nagar Haveli; DD, Daman and Diu; GA, Goa; GJ, Gujarat; HR, Haryana; HP, Himachal Pradesh; JK, Jammu & Kashmir; JH, Jharkhand; KA, Karnataka; KL, Kerala; LD, Lakshadweep; MP, Madhya Pradesh; MH, Maharashtra; MN, Manipur; ML, Meghalaya; MZ, Mizoram; NL, Nagaland; DL, New Delhi; OD, Odisha; PY, Puducherry; PB, Punjab; RJ, Rajasthan; SK, Sikkim; TN, Tamil Nadu; TR, Tripura; UP, Uttar Pradesh; UK, Uttarakhand; WB, West Bengal; TS, Telangana. * All the choropleth maps were generated in R programming software using spatial data from the DHS Spatial Data Repository. Permission to reproduce the map was obtained from DHS Program.

**A. Urban**


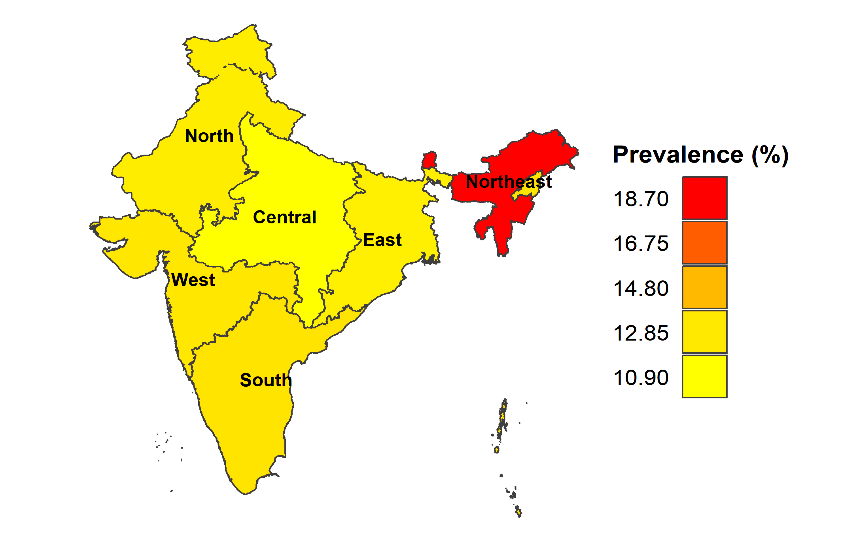

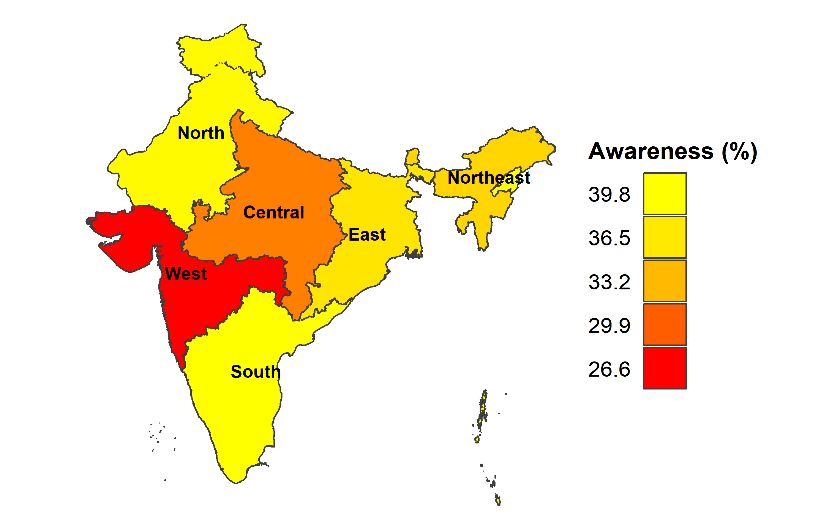

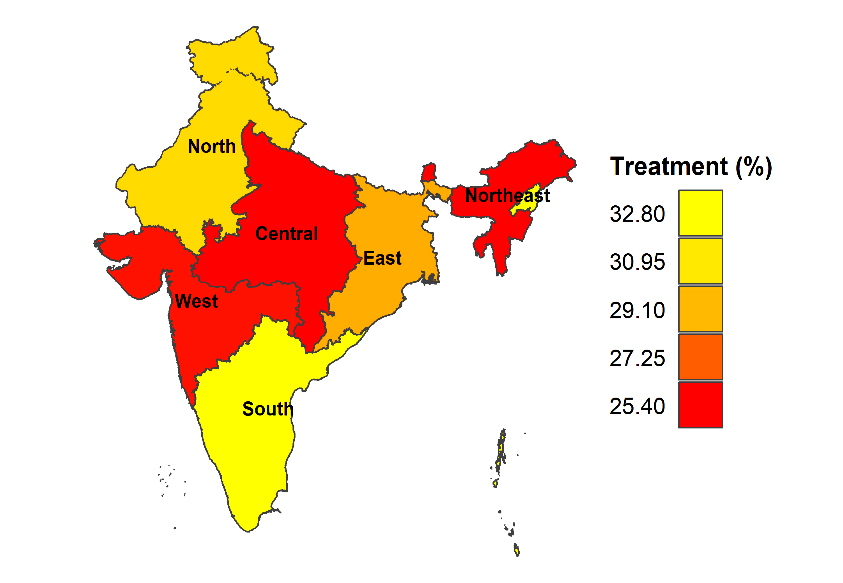

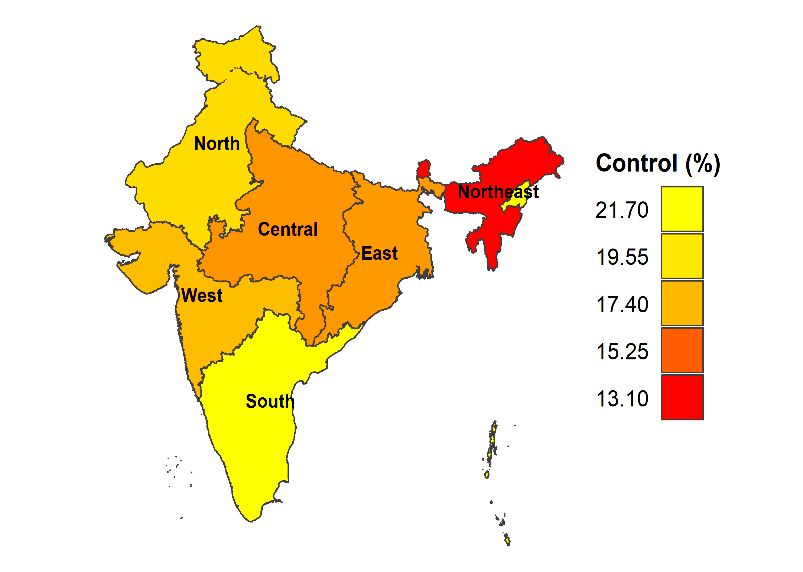


**B. Rural**

**
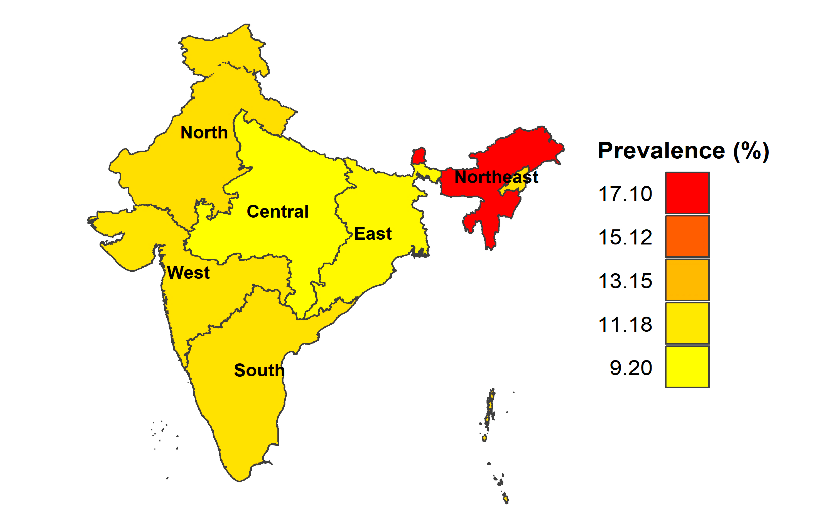

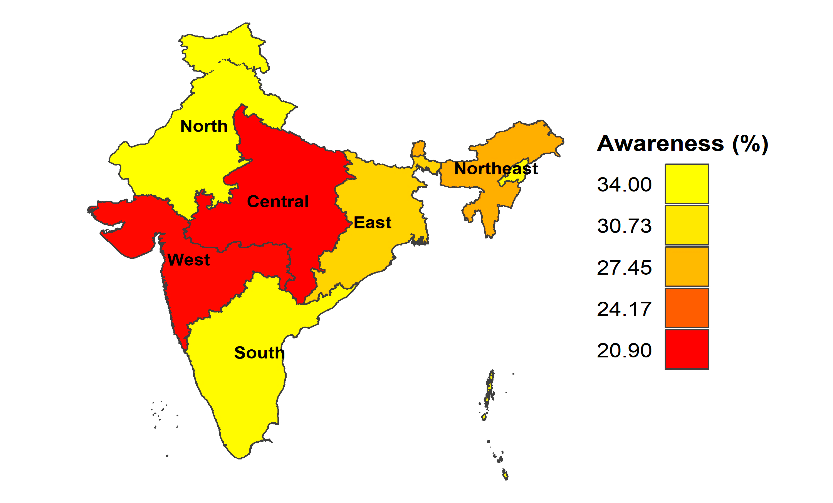

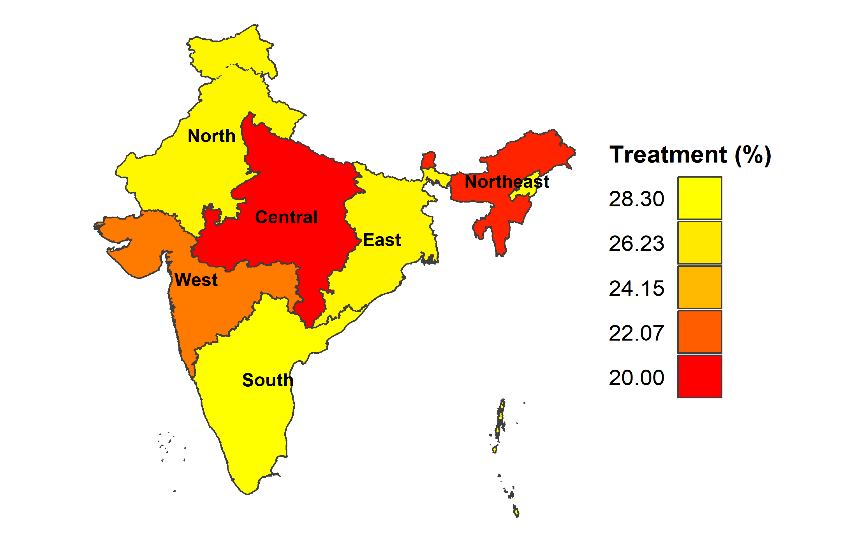

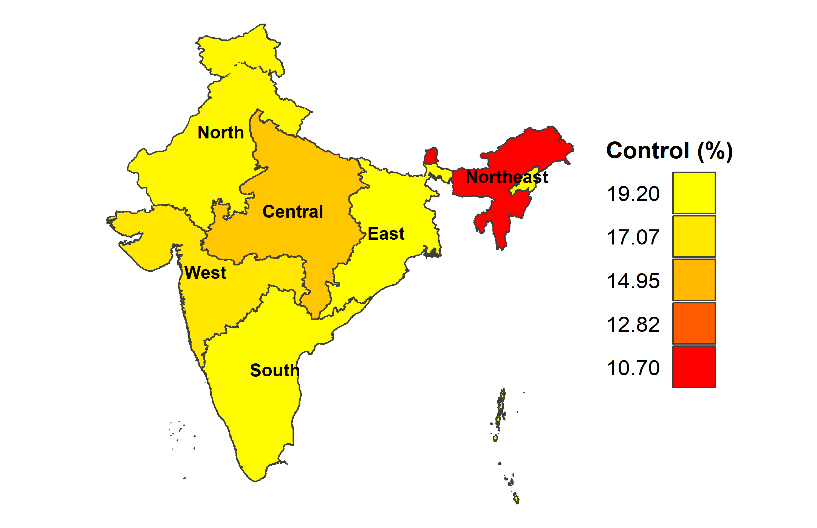
**

**Supplementary Figure 9. Prevalence and managemnet of hypertension at regional level by urban and rural residnece in India, 2016** * All the choropleth maps were generated in R programming software using spatial data from the DHS Spatial Data Repository. Permission to reproduce the map was obtained from DHS Program.

**A. Female**

**
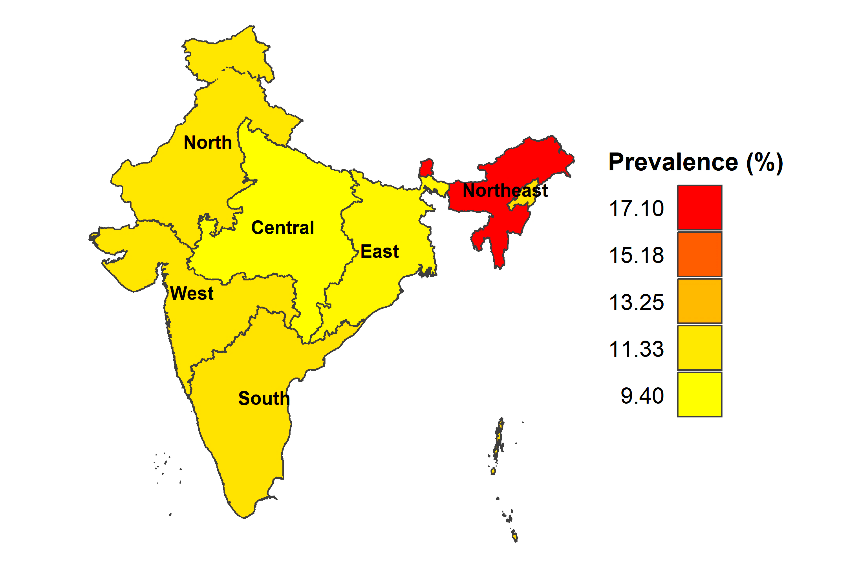

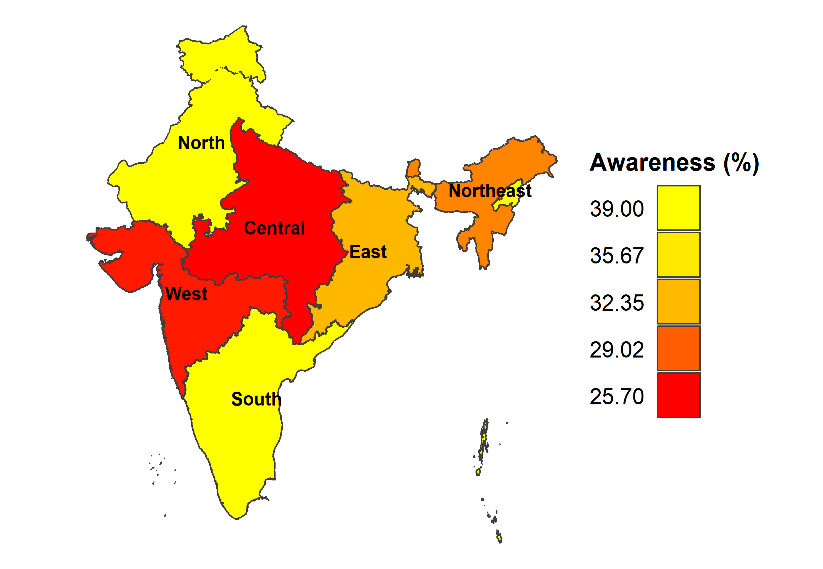

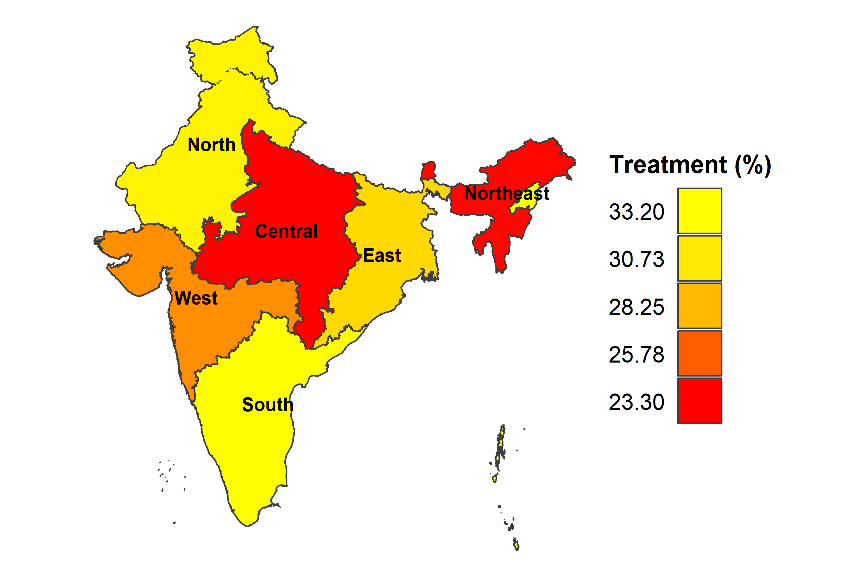

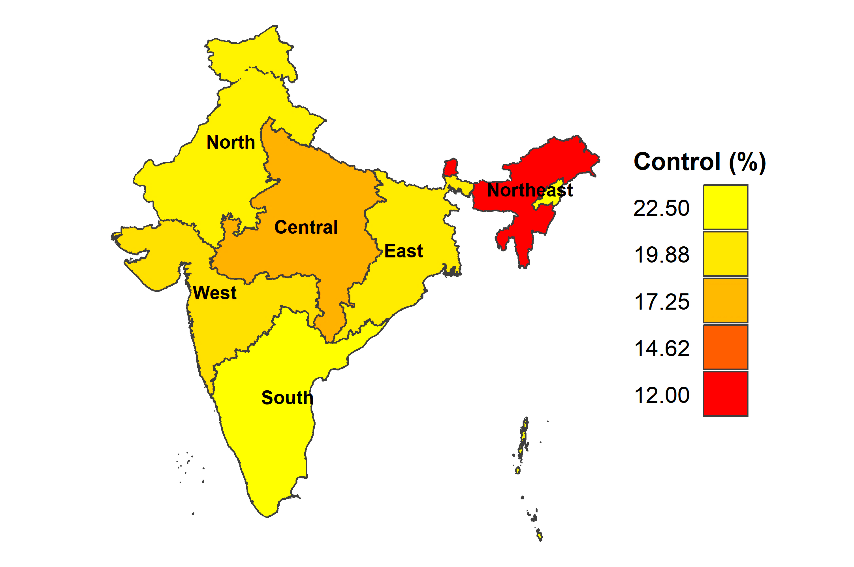
**

**B. Male**


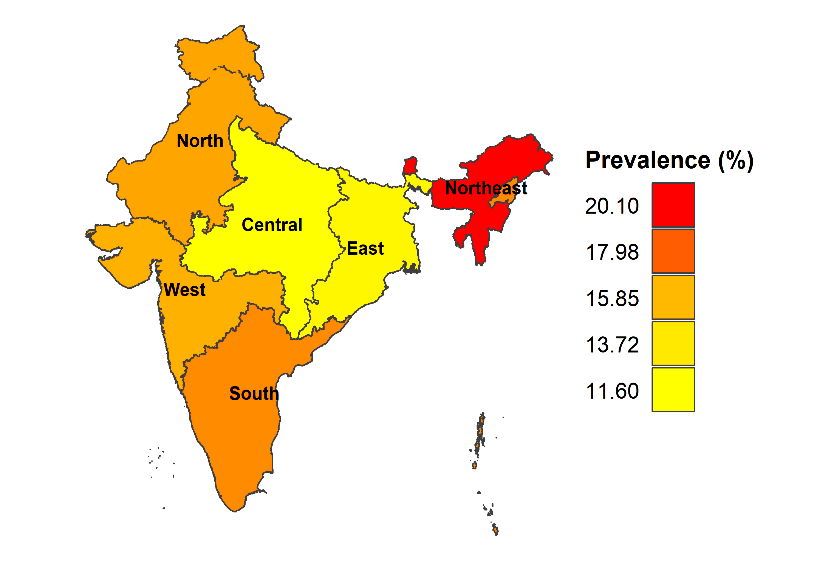

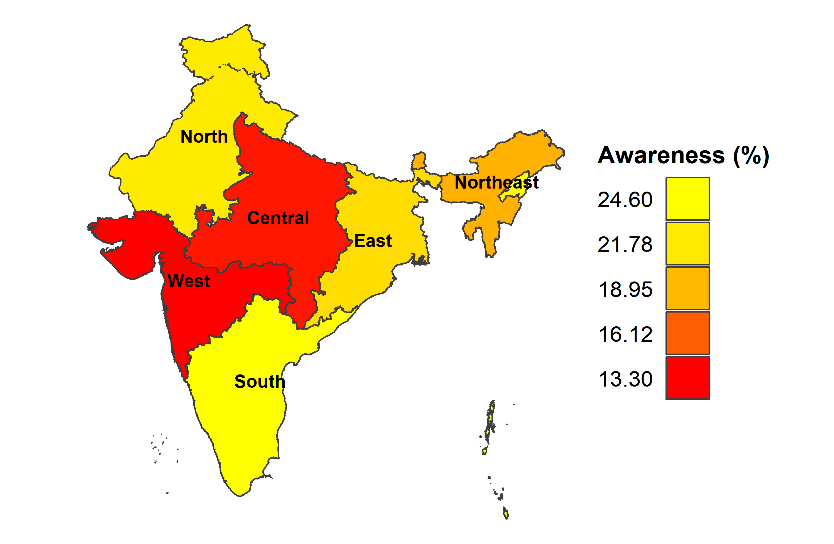


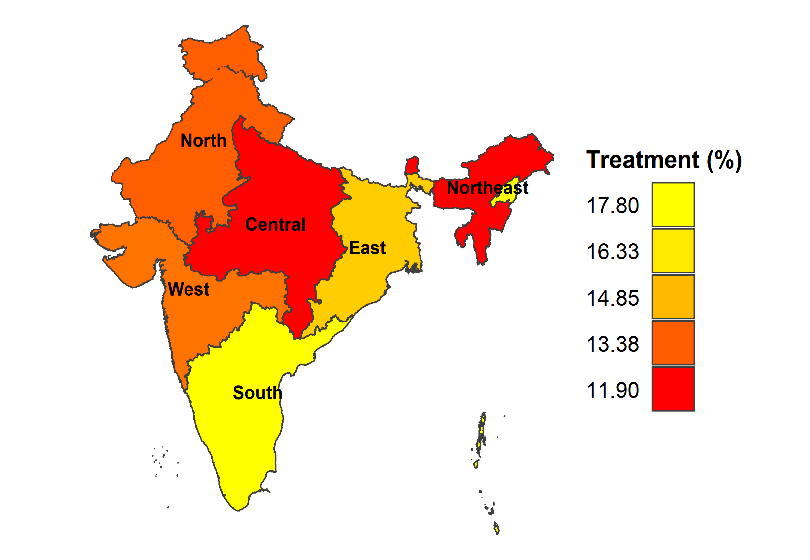

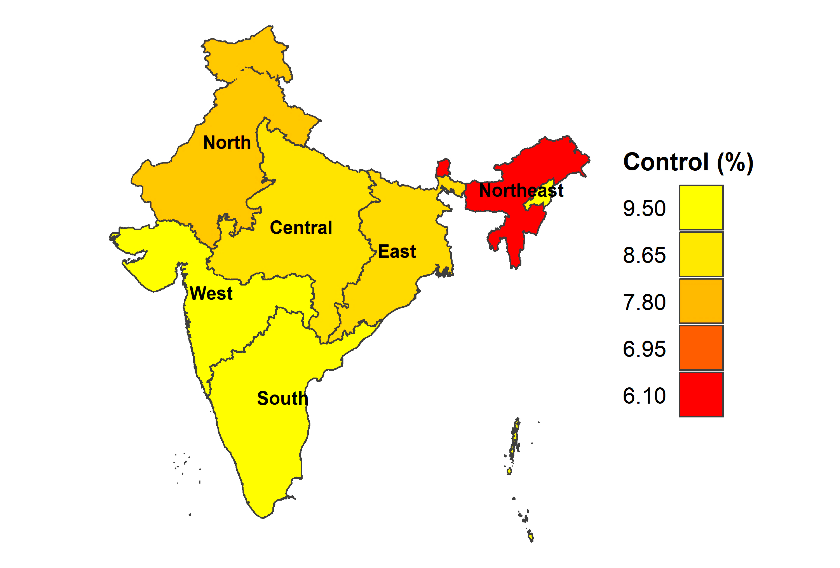


**Supplementary Figure 10. Prevalence and managemnet of hypertension at regional level by gender in India, 2016** * All the choropleth maps were generated in R programming software using spatial data from the DHS Spatial Data Repository. Permission to reproduce the map was obtained from DHS Program.

**A. India**


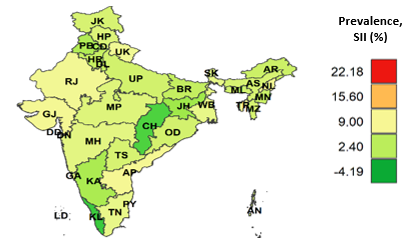

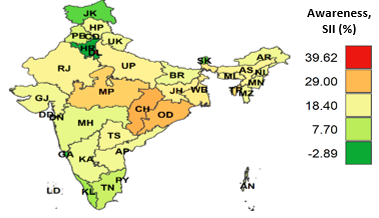


**B. Nepal**


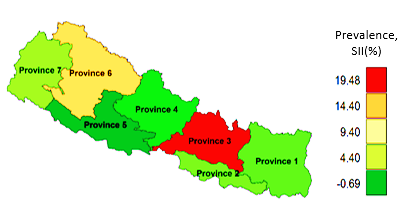

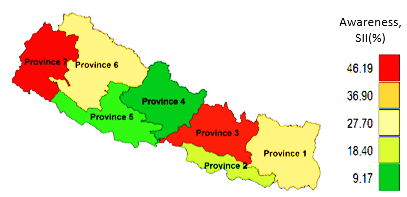


**Supplementary Figure 11. Wealth-based SII in prevalence and awareness of hypertension in India and Nepal.**

*SII = slope index of inequality; AN, Andaman and Nicobar Island; AP, Andhra Pradesh; AR, Arunachal Pradesh; AS, Assam; BR, Bihar; CD, Chandigarh; CH, Chhattisgarh; DN, Dadra and Nagar Haveli; DD, Daman and Diu; GA, Goa; GJ, Gujarat; HR, Haryana; HP, Himachal Pradesh; JK, Jammu & Kashmir; JH, Jharkhand; KA, Karnataka; KL, Kerala; LD, Lakshadweep; MP, Madhya Pradesh; MH, Maharashtra; MN, Manipur; ML, Meghalaya; MZ, Mizoram; NL, Nagaland; DL, New Delhi; OD, Odisha; PY, Puducherry; PB, Punjab; RJ, Rajasthan; SK, Sikkim; TN, Tamil Nadu; TR, Tripura; UP, Uttar Pradesh; UK, Uttarakhand; WB, West Bengal; TS, Telangana. * All the choropleth maps were generated in R programming software using spatial data from the DHS Spatial Data Repository. Permission to reproduce the map was obtained from DHS Program.

**A. India**

**Prevalence Awareness**

**
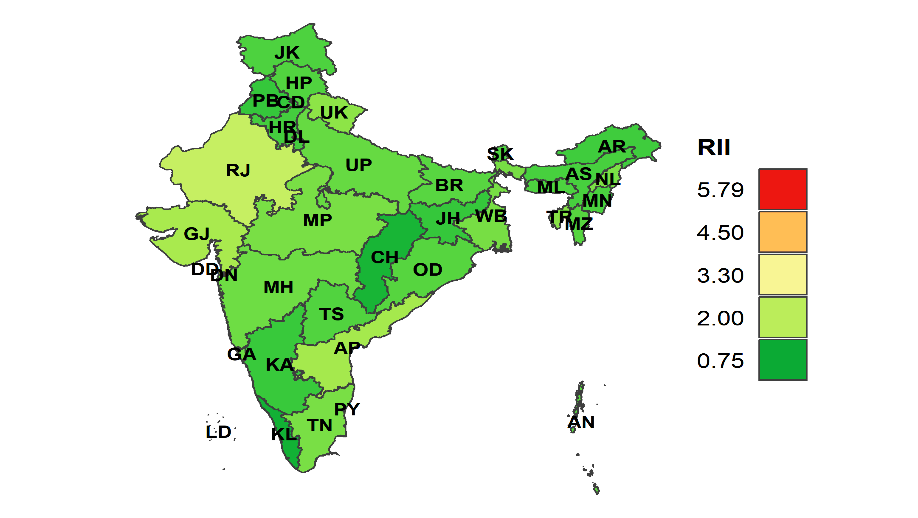

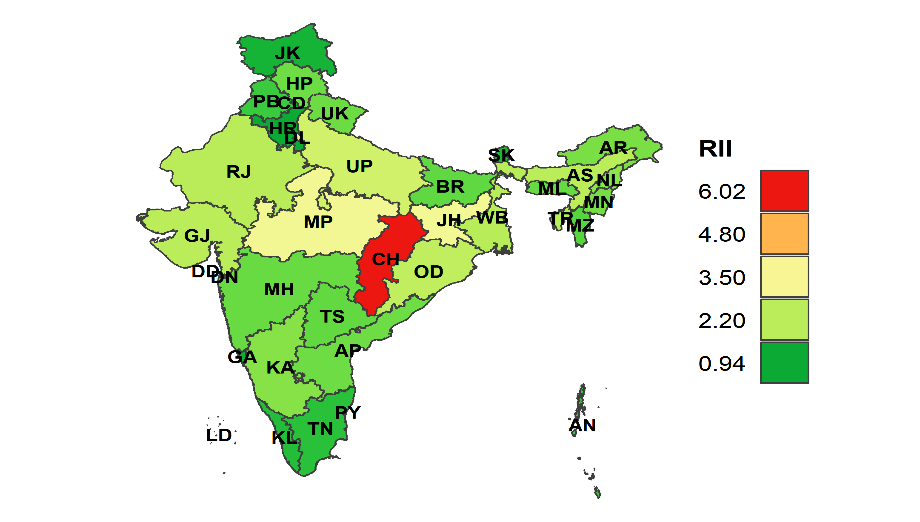
**

**Treatment Control**

**
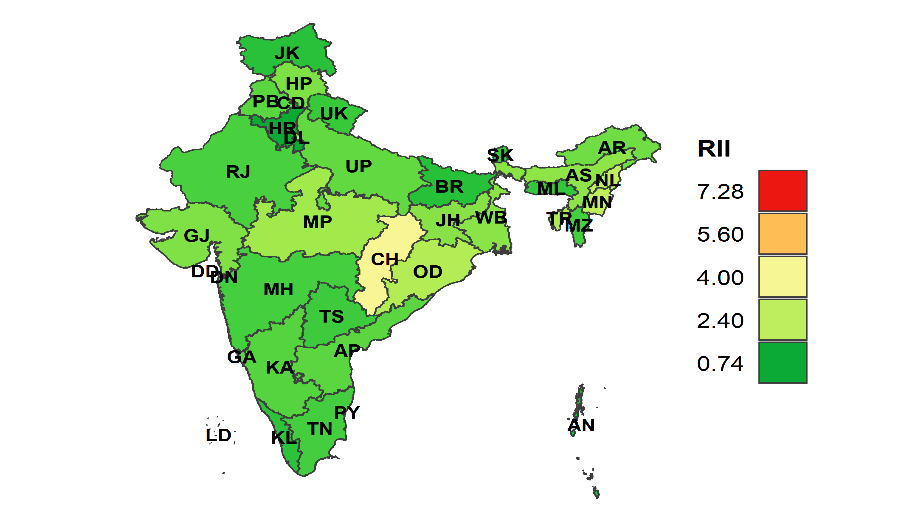

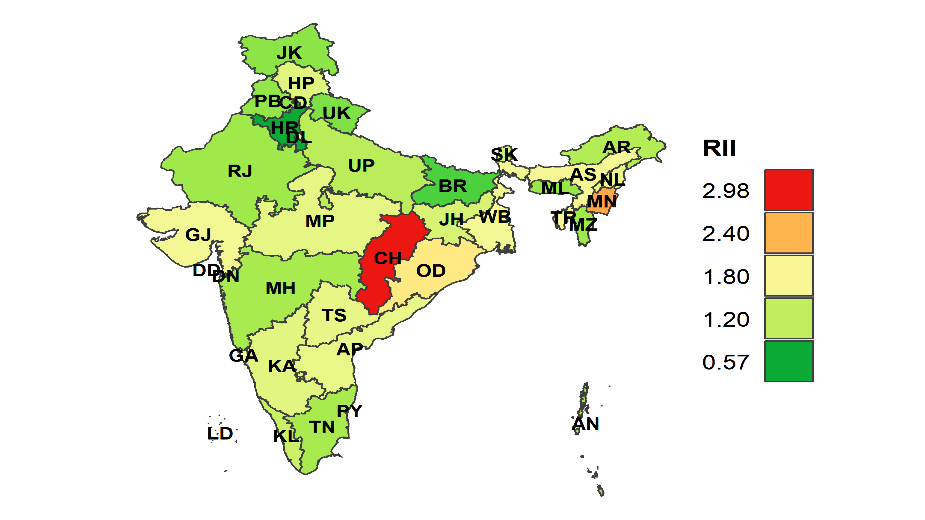
**

**B. Nepal**

**Prevalence Awareness**


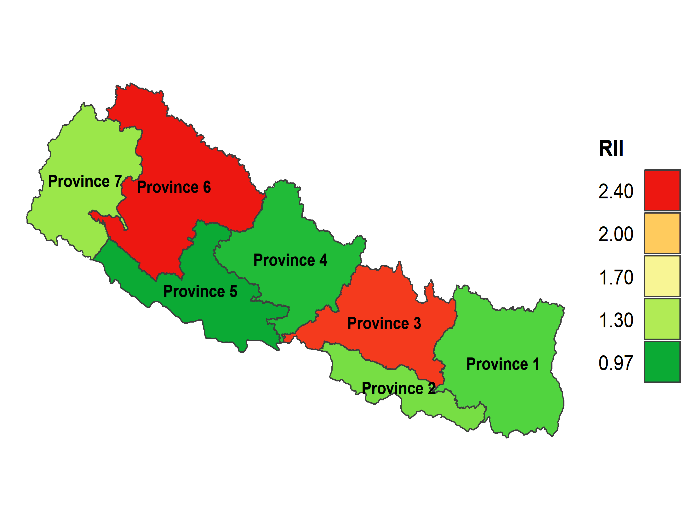

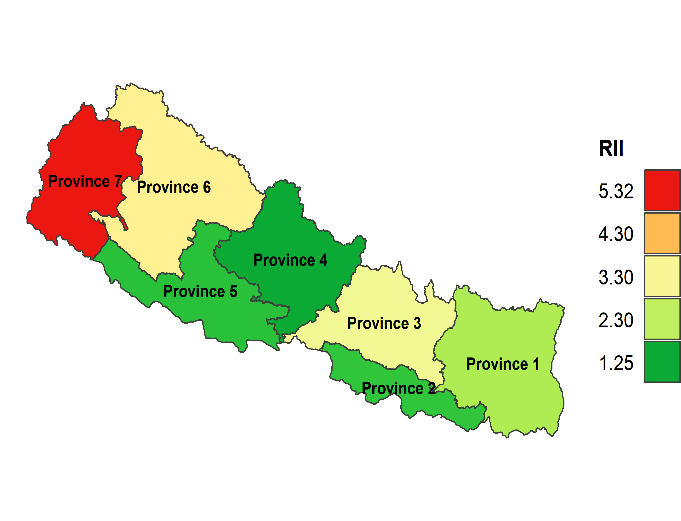


**Treatment Control**


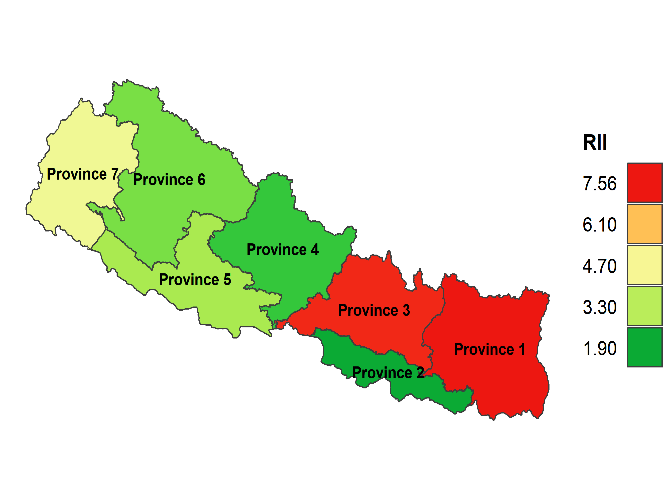

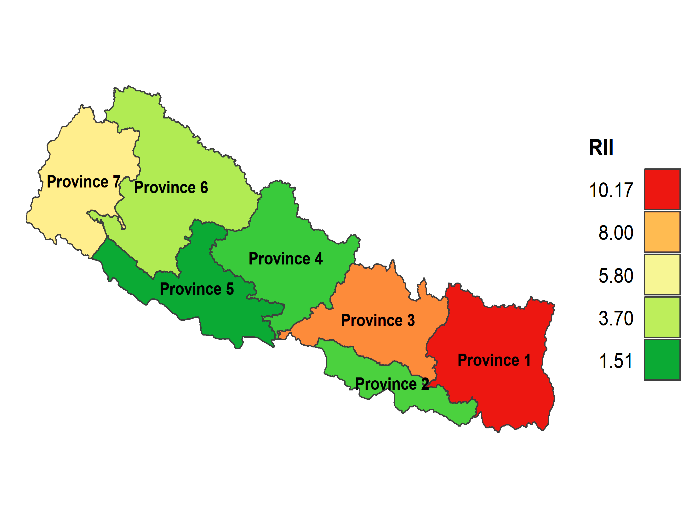


**Supplementary Figure 12. Wealth-based relative index of inequalities in prevalence and management of hypertension at subnational level in India and Nepal, 2016**

*RII = relative index of inequality; AN, Andaman and Nicobar Island; AP, Andhra Pradesh; AR, Arunachal Pradesh; AS, Assam; BR, Bihar; CD, Chandigarh; CH, Chhattisgarh; DN, Dadra and Nagar Haveli; DD, Daman and Diu; GA, Goa; GJ, Gujarat; HR, Haryana; HP, Himachal Pradesh; JK, Jammu & Kashmir; JH, Jharkhand; KA, Karnataka; KL, Kerala; LD, Lakshadweep; MP, Madhya Pradesh; MH, Maharashtra; MN, Manipur; ML, Meghalaya; MZ, Mizoram; NL, Nagaland; DL, New Delhi; OD, Odisha; PY, Puducherry; PB, Punjab; RJ, Rajasthan; SK, Sikkim; TN, Tamil Nadu; TR, Tripura; UP, Uttar Pradesh; UK, Uttarakhand; WB, West Bengal; TS, Telangana. * All the choropleth maps were generated in R programming software using spatial data from the DHS Spatial Data Repository. Permission to reproduce the map was obtained from DHS Program.

**A. India**

Prevalence Awareness


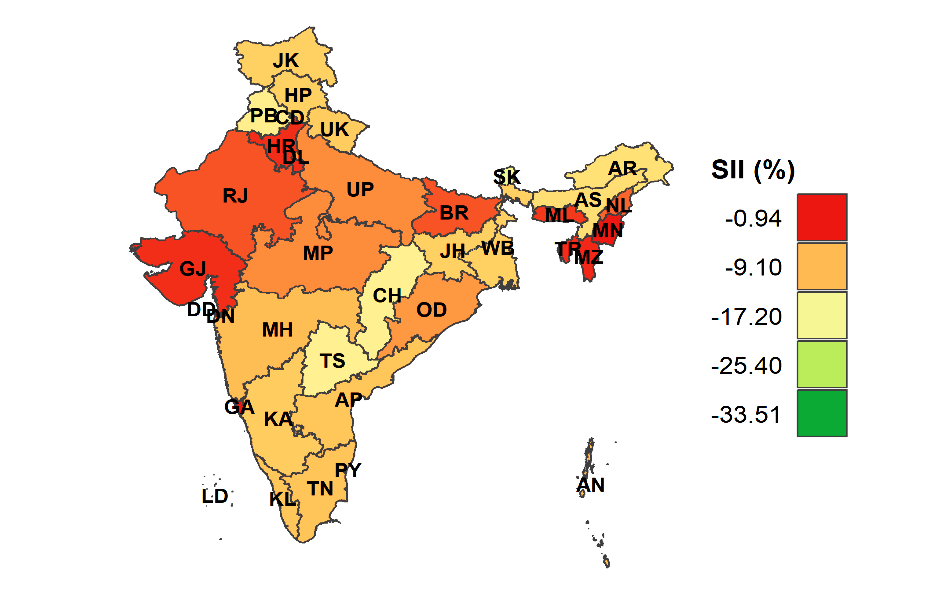

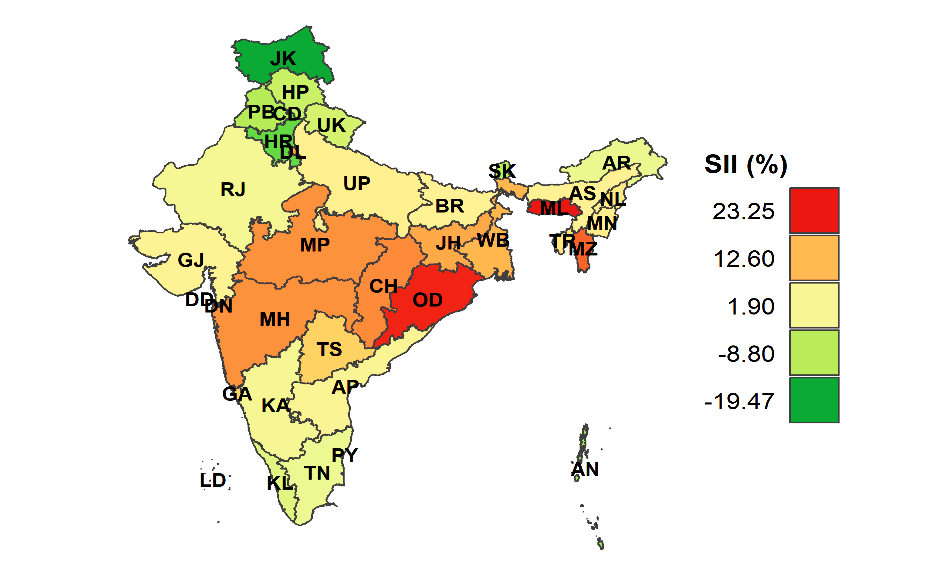


Treatment Control
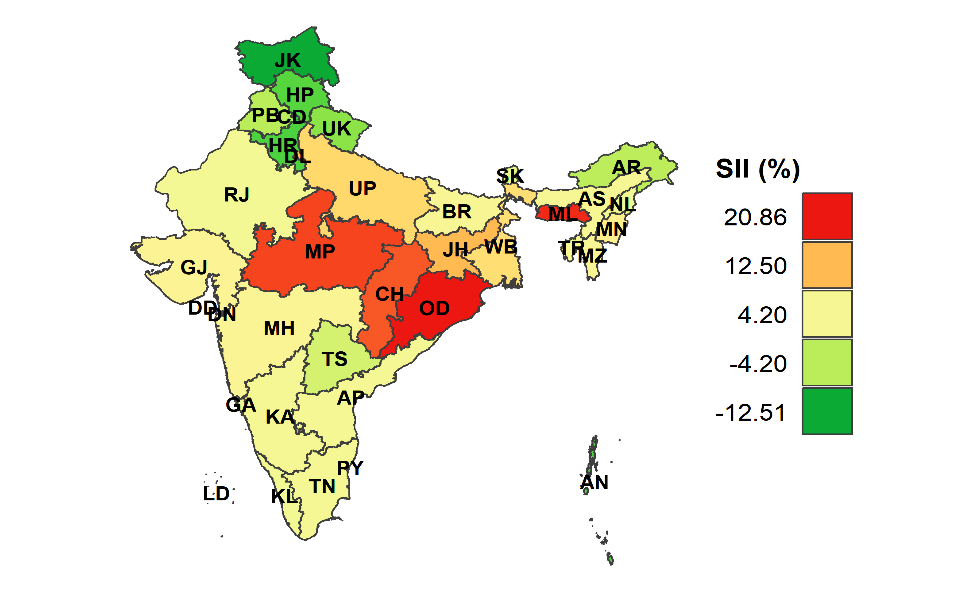

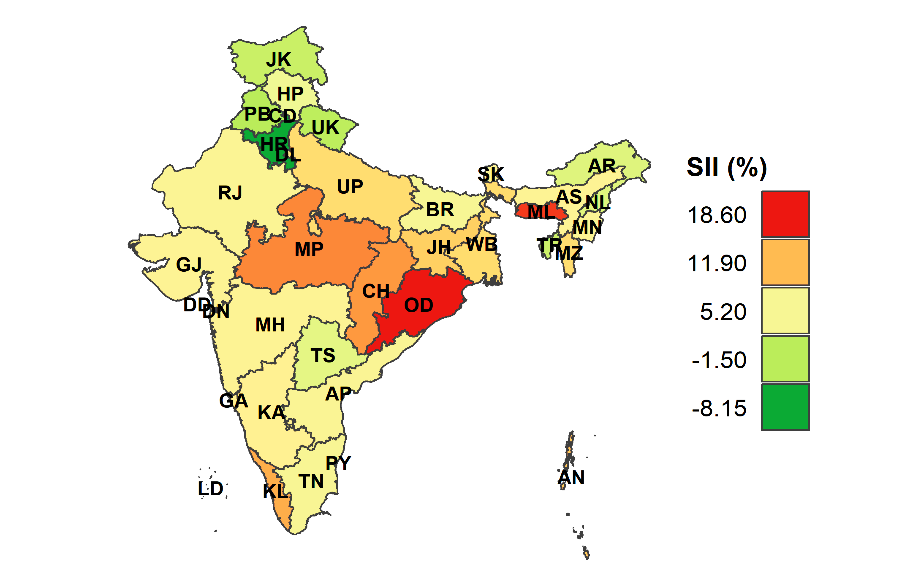


**B. Nepal**

Prevalence Awarness


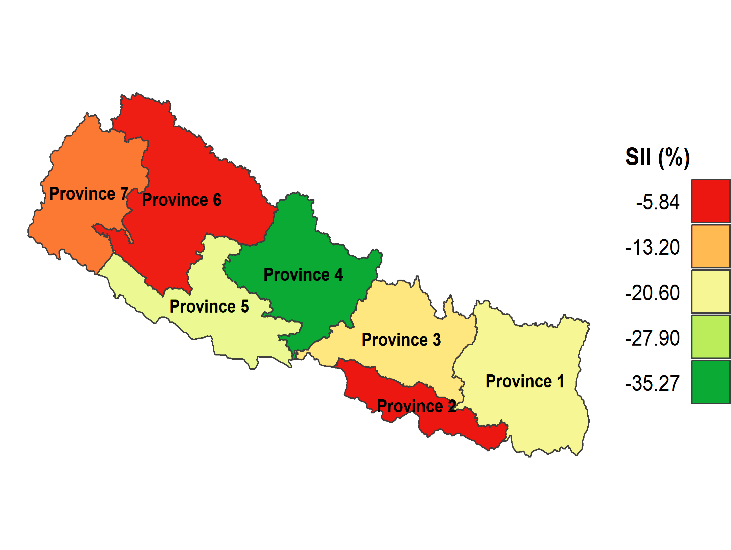

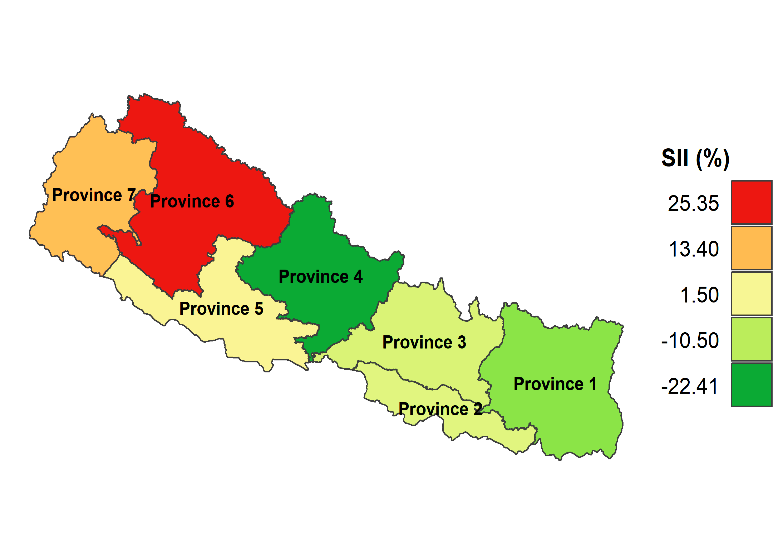


Treatment Control


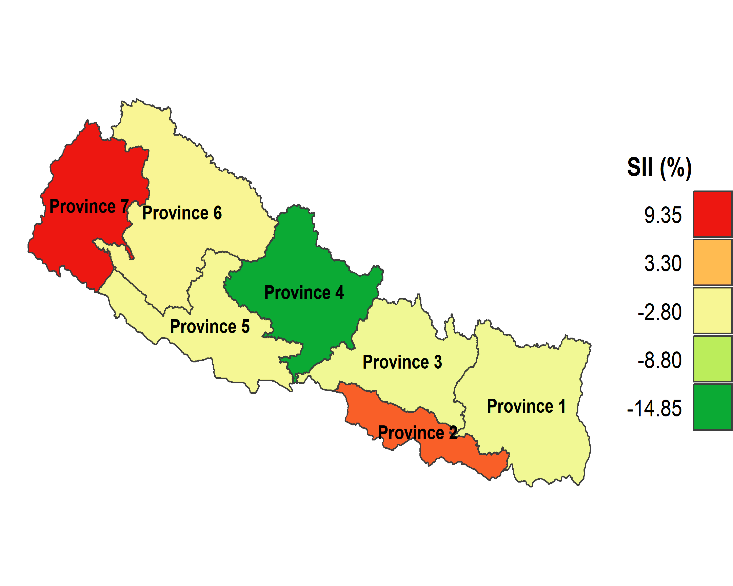

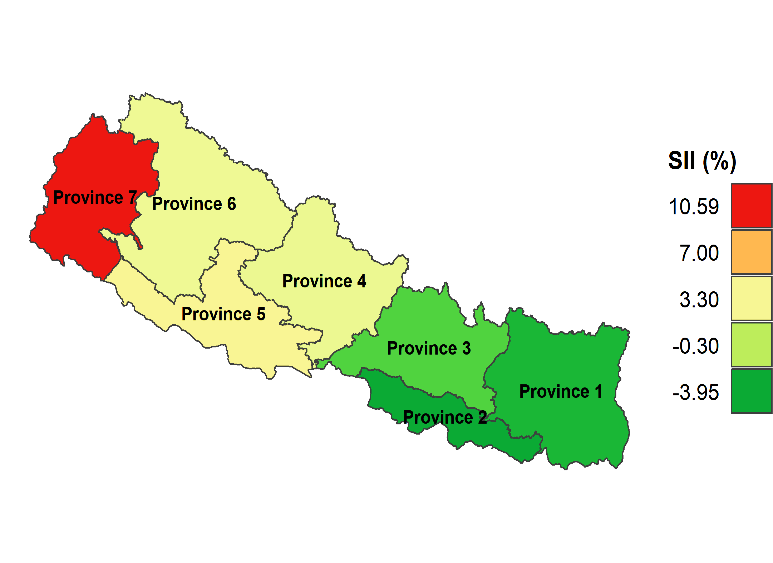


**Supplementary Figure 13. Education-based slope index of inequalities in prevalence and management of hypertension at subnational level in India and Nepal, 2016**

*SII = slope index of inequality; AN, Andaman and Nicobar Island; AP, Andhra Pradesh; AR, Arunachal Pradesh; AS, Assam; BR, Bihar; CD, Chandigarh; CH, Chhattisgarh; DN, Dadra and Nagar Haveli; DD, Daman and Diu; GA, Goa; GJ, Gujarat; HR, Haryana; HP, Himachal Pradesh; JK, Jammu & Kashmir; JH, Jharkhand; KA, Karnataka; KL, Kerala; LD, Lakshadweep; MP, Madhya Pradesh; MH, Maharashtra; MN, Manipur; ML, Meghalaya; MZ, Mizoram; NL, Nagaland; DL, New Delhi; OD, Odisha; PY, Puducherry; PB, Punjab; RJ, Rajasthan; SK, Sikkim; TN, Tamil Nadu; TR, Tripura; UP, Uttar Pradesh; UK, Uttarakhand; WB, West Bengal; TS, Telangana. * All the choropleth maps were generated in R programming software using spatial data from the DHS Spatial Data Repository. Permission to reproduce the map was obtained from DHS Program.

**A. India**

**Prevalence Awareness**

**
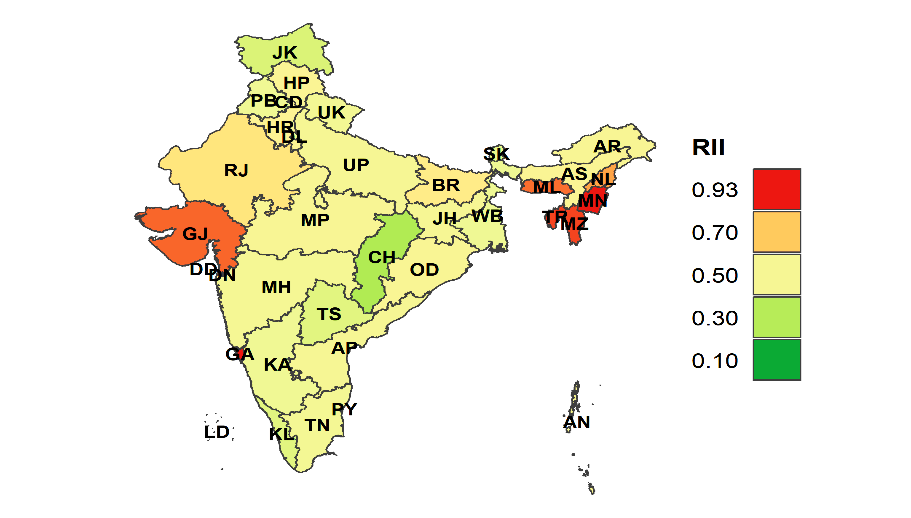
**
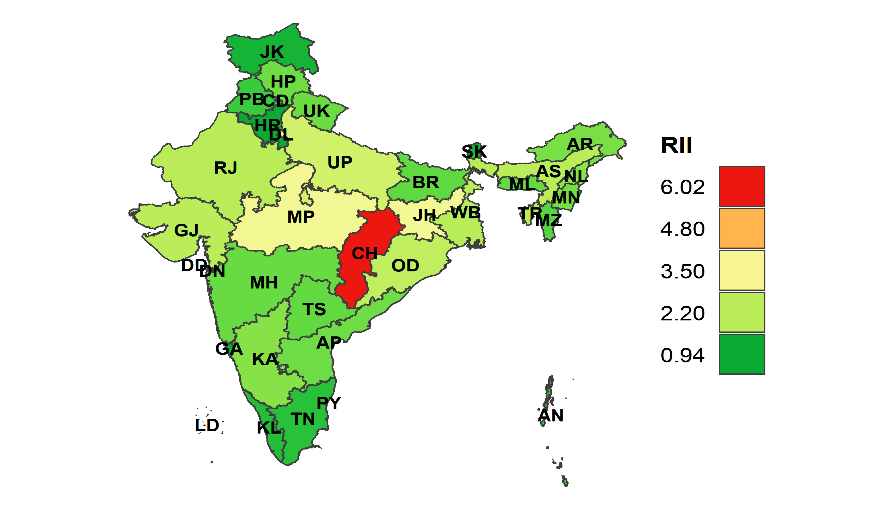


**Treatment Control**

**
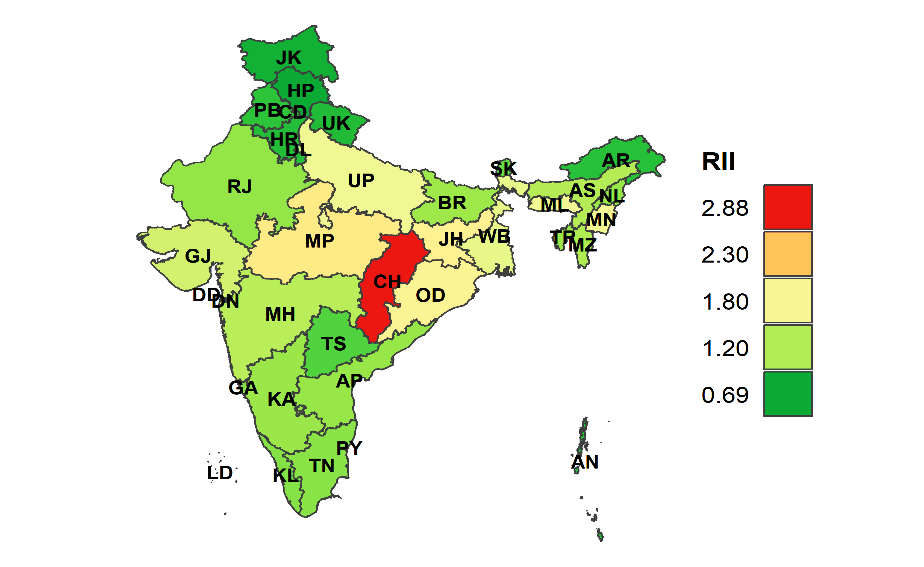

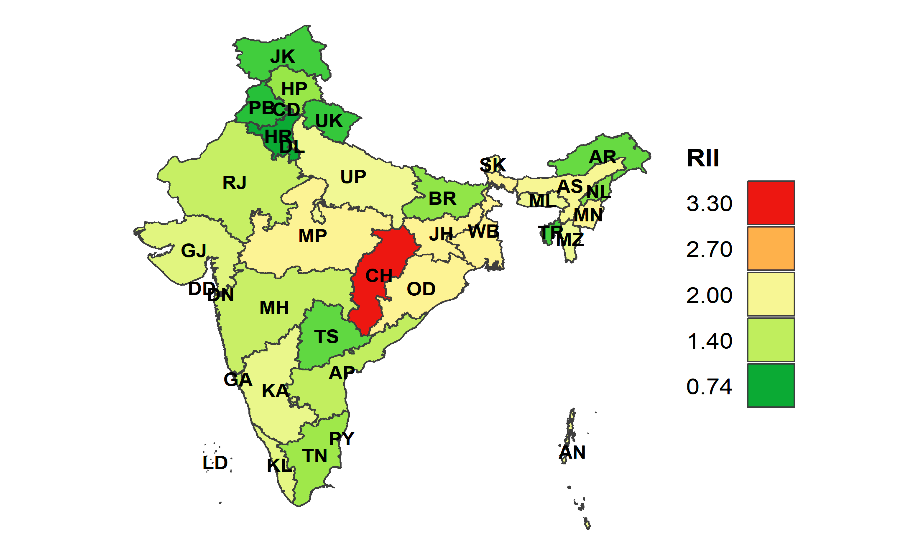
**

**B. Nepal**

**Prevalence Awareness**

**
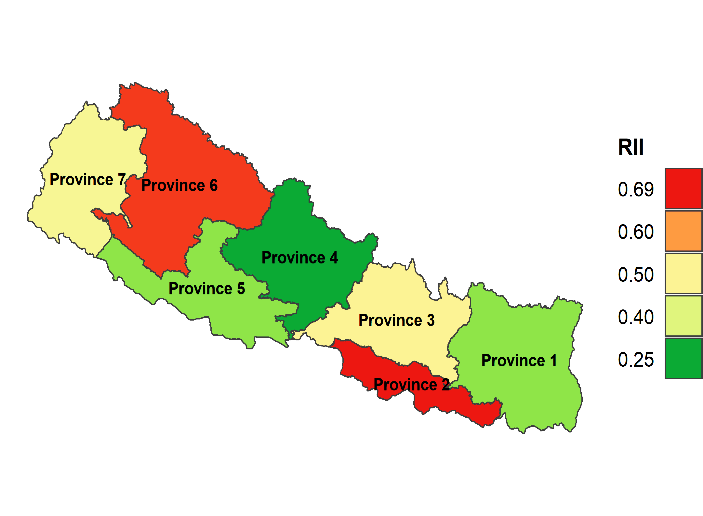

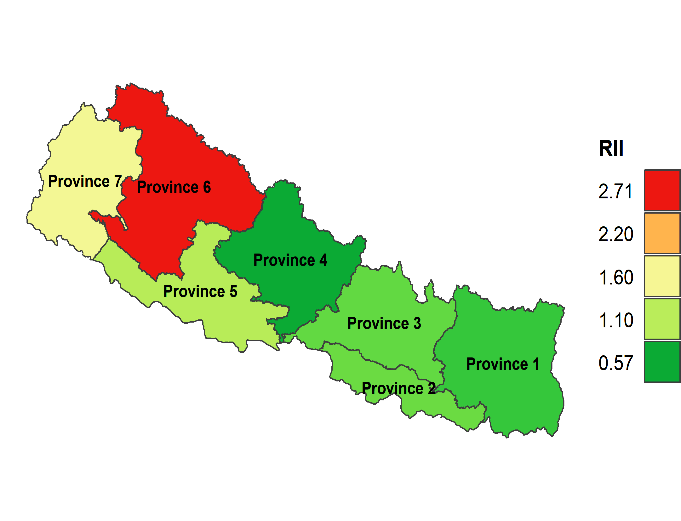
**

**Treatment Control**

**
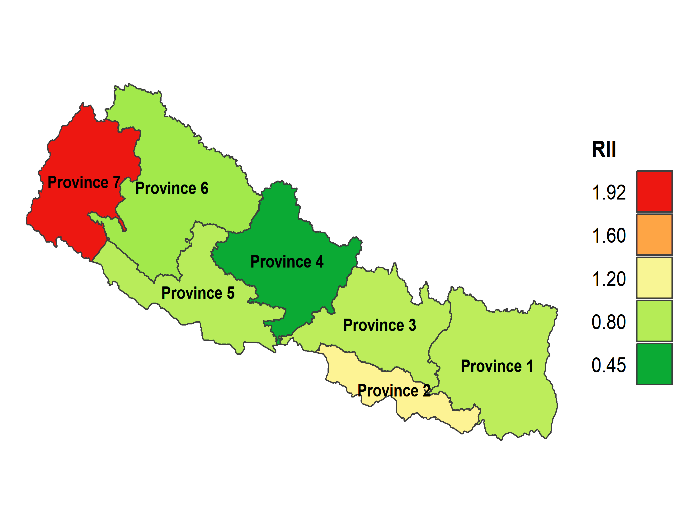

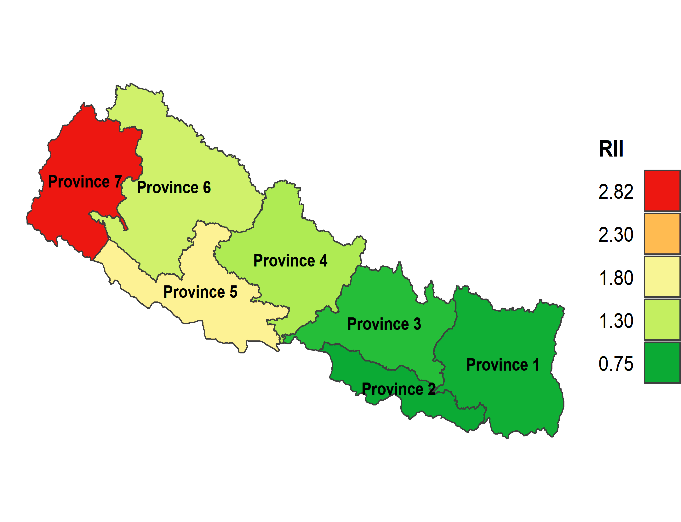
**

**Supplementary Figure 14. Education-based relative index of inequalities in prevalence and management of hypertension at subnational level in India and Nepal, 2016**

*RII = relative index of inequality; AN, Andaman and Nicobar Island; AP, Andhra Pradesh; AR, Arunachal Pradesh; AS, Assam; BR, Bihar; CD, Chandigarh; CH, Chhattisgarh; DN, Dadra and Nagar Haveli; DD, Daman and Diu; GA, Goa; GJ, Gujarat; HR, Haryana; HP, Himachal Pradesh; JK, Jammu & Kashmir; JH, Jharkhand; KA, Karnataka; KL, Kerala; LD, Lakshadweep; MP, Madhya Pradesh; MH, Maharashtra; MN, Manipur; ML, Meghalaya; MZ, Mizoram; NL, Nagaland; DL, New Delhi; OD, Odisha; PY, Puducherry; PB, Punjab; RJ, Rajasthan; SK, Sikkim; TN, Tamil Nadu; TR, Tripura; UP, Uttar Pradesh; UK, Uttarakhand; WB, West Bengal; TS, Telangana. * All the choropleth maps were generated in R programming software using spatial data from the DHS Spatial Data Repository. Permission to reproduce the map was obtained from DHS Program.

**A. Wealth-based SII**

**Prevalence Awareness**

**Treatment Control**

**B. Education-based SII**

**Prevalence Awareness**

**Treatment Control**

**Supplementary Figure 15. Slope index of inequalities in prevalence and management of hypertension by wealth quintile and education at regional level in India, 2016**

SII = slope index of inequality; * All the choropleth maps were generated in R programming software using spatial data from the DHS Spatial Data Repository. Permission to reproduce the map was obtained from DHS Program.

**A. India**

**B. Nepal**

**Supplementary Figure 16. Decomposition of concentration index, India and Nepal 2016**

* All the bar chart was generated using excel.

**Supplementary Figure 17. Equiplots for prevalence of hypertension by household wealth quintile in India, 2016.**

* All the Equiplots were generated using Stata 15 software.

**Supplementary Figure 18. Equiplots for awareness, of hypertension by household wealth quintile in India, 2016.**

* All the Equiplots were generated using Stata 15 software.

**Supplementary Figure 19. Equiplots for treatment of hypertension by household wealth quintile in India, 2016.**

* All the Equiplots were generated using Stata 15 software.

**Supplementary Figure 20. Equiplots for control of hypertension by household wealth quintile in India, 2016.**

* All the Equiplots were generated using Stata 15 software.

**Supplementary Figure 21. Equiplots for prevalence of hypertension by household wealth quintile in Nepal, 2016.**

**Supplementary Figure 22. Equiplots for awareness of hypertension by household wealth quintile in Nepal, 2016.**

* All the Equiplots were generated using Stata 15 software.

**Supplementary Figure 23. Equiplots for treatment of hypertension by household wealth quintile in Nepal, 2016.**

**Supplementary Figure 24. Equiplots for control of hypertension by household wealth quintile in Nepal, 2016.**

* All the Equiplots were generated using Stata 15 software

**Supplementary Figure 25. Equiplots for prevalence of hypertension by level of education in India, 2016.**

* All the Equiplots were generated using Stata 15 software.

**Supplementary Figure 26. Equiplots for awareness of hypertension by level of education in India, 2016.**

* All the Equiplots were generated using Stata 15 software.

**Supplementary Figure 27. Equiplots for treatment of hypertension by level of education in India, 2016.**

* All the Equiplots were generated using Stata 15 software.

**Supplementary Figure 28. Equiplots for control of hypertension by level of education in India, 2016.**

* All the Equiplots were generated using Stata 15 software.

**Supplementary Figure 29. Equiplots for prevalence of hypertension by level of education in Nepal, 2016**

**Supplementary Figure 30. Equiplots for awareness of hypertension by level of education in Nepal, 2016.**

* All the Equiplots were generated developed using Stata 15 software.

**Supplementary Figure 31. Equiplots for treatment of hypertension by level of education in Nepal, 2016**

**Supplementary Figure 32. Equiplots for control of hypertension by level of education in Nepal, 2016.**

* All the Equiplots were generated using Stata 15 software.

**Supplementary Method 1. Data Collection**

**Data collection in India**

The National Family Health Survey 2015-2016 (NHFS-4), is a nationally representative population survey conducted under the stewardship of Ministry of Health and Family Welfare (MoHFW), Government of India. It is the fourth NFHS series that provides the information on population, health and nutrition for India and each state/union territory. The clinical, anthropometric and biochemical (CAB) component of NFHS-4 provides information about hypertension through series of biomarker tests and measurements.

NFHS-4 used multistage stratified cluster sampling method to collect data from households nation-wide. The 2011 census in India was used as the sampling frame. Census Enumeration Blocks (CEBs) in urban areas and rural areas were compiled which served as primary sampling units PSUs for NFHS-4 sample. Stratification for NFHS-4 sample was achieved by separating each district into urban and rural areas. Within each rural stratum, six approximately equal substrata were created based on crossing three sub-strata. Within each explicit rural sampling stratum, and before the primary sampling units (PSUs) selection, PSUs were sorted according to the literacy rate of women age 6+ years. Within each urban sampling stratum, and before the PSUs selection, PSUs were sorted according to the percentage of SC/St population.

In the first stage, 9,938 PSUs were randomly selected. In every selected rural and urban PSU, a complete household mapping and listing operating was conducted prior to the main survey. Selected PSUs with an estimated number of at least 300 households were segmented into segments of approximately 100 – 150 households. Two of the segments were selected for the survey using systematic sampling with probability proportional to segment size. In second stage, in every selected rural and urban cluster, 22 households were selected using systematic sampling with probability proportional to segment size.

Out of total population of 1.32 billion, 811,808 individuals (699,686 women and 112,122 men) were selected from 601,506 households (175,946 in urban and 435,563 in rural) with over all response rate of 94.3% (96.7% women and 91.9% men).

Blood pressure was measured for eligible women aged 15-49 and (in the state module subsample of households only) eligible men aged 15-54 years, who stayed in the household the night before the household interview (including both usual residents and visitors). Blood pressure measurements for each respondent were taken three times with an interval of five minutes between readings using an Omron blood pressure monitors by the trained field staffs.

**Data collection in Nepal**

The Nepal Demographic and Health Survey 2016 (NDHS), is a nationally representative population survey conducted under the aegis of the Ministry of Health (MOH), Government of Nepal. It is the fifth NDHS series that provides up-to-date estimated of basic demographic and health indicators for Nepal and each ecological zone (Terai, Hills, and Mountains) and province (Province 1-7). The clinical, anthropometric and biochemical (CAB) component of NDHS 2016 provides information about hypertension through series of biomarker tests and measurements.

The 2016 NDHS used multistage stratified cluster sampling method to collect data from households nation-wide like India. The sampling frame used for 2016 NDHS was updated version of the frame from the 2011 National Population and Housing Census (NPHC). The 2016 NDHS included 263 municipalities and 7 provinces which were not in NPHC 2011). Samples were stratified and selected in two stages in rural areas and three stages in urban areas. The sampling frame contained information about ward location, type of residence (urban and rural), estimated number of residential households and estimated population. In rural areas, wards were small (average of 104 households) and served as the primary sampling units PSUs and households were selected from these PSUs. In urban areas, wards were large, with average of 800 households per ward. These wards were further segmented into small enumeration areas (EAs). Selected EAs served as PSUs and households were selected from them.

In the first stage, 383 wards were selected with probability proportional to ward size and with independent selection in each sampling stratum. Due to large size of the urban wards, in a second stage of sample selection, on EA was randomly selected from each of the sample urban ward. A household listing was done in all of the selected sampling clusters (rural wards and urban EAs), and the resulting lists of households served as the sampling frame for the selection of households in the next stage. Some of the large clusters with more than 200 households were segmented further to minimize the task of household listing. In last stage of selection, a fixed number of 30 households per cluster were selected with an equal probability systematic selection.

Out of total population 28.98 million in Nepal, 14,701 individuals (8,435 women and 6,266 men) from 11,040 households (6,978 in urban and 4,062 in rural) were selected with overall response rate of 96% (97% women and 95% men).

Blood pressure was measured in women and men aged 15-69 years, who stayed in the household the night before the household interview (including both usual residents and visitors). Blood pressure measurements for each respondent were taken three times with an interval of five minutes between readings using UA-767F/FAC (A&D medical) blood pressure monitors by the trained field staffs.

**Supplementary Method 2. Sampling weight**

Due to non-proportional allocation of the sample to the different survey domains and to their urban and rural areas, sampling weights were required in both countries. Since the survey was carried out in two-stage stratified cluster sample, sampling weights were calculated based on sampling probabilities separately for each sampling stage and for each cluster in both the countries.

*P_1ht_:* first-stage sampling probability of the *i^th^* cluster in stratum *h.*

*P_2ht_:* second-stage sampling probability within the *i^th^* cluster (household selection)

Let *a_h_* be the number of clusters selected in stratum h, let *M_hi_* be the number of households according to the sampling frame in the *i^th^* cluster, and let *∑* ***_h_*** *M_hi_* be the total number of households in stratum *h*. The probability of selecting the *i^th^* cluster in stratum h in the sample is calculated as follows:

$\frac{\boldsymbol{a}_{\boldsymbol{h}}\boldsymbol{M}_{\boldsymbol{hi}}}{\boldsymbol{\sum}\boldsymbol{M}_{\boldsymbol{hi}}}$

Let *b_hi_* be the proportion of household in the selected segments with respect to the total number of households in the PSU I in stratum h if the PSU is segmented; otherwise, *b_hi_* = 1. Then, the probability of selecting cluster I in the sample is:

$\boldsymbol{P}_{\boldsymbol{1}\boldsymbol{hi}}$ **=** $\frac{\boldsymbol{a}_{\boldsymbol{h}}\boldsymbol{M}_{\boldsymbol{hi}}}{\boldsymbol{\sum M}_{\boldsymbol{hi}}}$ *** *b_hi_***

In Case of three stage sampling in urban areas (Nepal)

Let *b_hi_* be the proportion of households in the selected EA compared with the total number of households in ward *I* in stratum *h* for the urban strata. Otherwise *b_hi_* = 1. Let *c_hi_* be the proportion of households in the selected segment compared with the total number of households in ward or EA *i* in stratum *h*, if the ward or the EA is segmented. Otherwise *c_hi_* _­_=1. Then the probability of selecting cluster *i* in the sample is:

$\boldsymbol{P}_{\boldsymbol{1}\boldsymbol{hi}}$ **=** $\frac{\boldsymbol{a}_{\boldsymbol{h}}\boldsymbol{M}_{\boldsymbol{hi}}}{\boldsymbol{\sum M}_{\boldsymbol{hi}}}$ *** *b_hi­­­_* ******c_hi_***

_­_Let $L_{hi}$ be the number of households listed in the household listing operation in cluster *i* in stratum *h,* let $g_{hi}$ be the number of households selected in the cluster. The last stage’s selection probability for each household in the cluster is calculated as follows:

$$\boldsymbol{P}_{\boldsymbol{2}\boldsymbol{hi}}\mathbf{=}\frac{\boldsymbol{g}_{\boldsymbol{hi}}}{\boldsymbol{L}_{\boldsymbol{hi}}}$$

The overall selection probability of each household in cluster *i* of stratum *h* is therefore the product of selection probabilities:

$\boldsymbol{P}_{\boldsymbol{hi}}\boldsymbol{=}\boldsymbol{P}_{\boldsymbol{1}\boldsymbol{hi}}$***** $\boldsymbol{P}_{\boldsymbol{2}\boldsymbol{hi}}$

The sampling weight for each household in cluster *i* of stratum *h* is the inverse of its total selection probability:

$\boldsymbol{W}_{\boldsymbol{hi}}\boldsymbol{=1/}\boldsymbol{P}_{\boldsymbol{hi}}$

**Supplementary Method 3. Independent variables**

***Individual***-***level characteristics:***

Age: Age group was categorized differently in India and Nepal. For India, the age group included was 15–49 years. Whereas, for Nepal, age group included was 15–69 years and above. Age group above 49 years was not included in India due to limited data availability.

Gender: Gender was categorized as men and women.

Education: Educational level was stratified into four levels based on the household questionnaire namely, no education, primary education, secondary education, and higher education.

Body mass index (BMI): BMI was calculated as weight (kg) / height (m^2^) and categorized into four groups following the WHO categorization.[^42^](#_ENREF_42) Underweight was defined as the individuals having BMI less than 18.5 kg/m^2^. BMI of 18.5–24.9 kg/m^2^ were categorized as normal weight. BMI between 25.0–29.9 kg/m^2^ was defined as overweight and BMI 30.0 or more was grouped as obese.

Alcohol consumption: Alcohol consumption was binary variable with options ‘Yes’ if the participants had/have been consuming alcohol and option ‘No’ for no consumption.

Tobacco consumption: It was a binary variable with option ‘Yes’ if the participants had/have been smoking or taking any tobacco product and option ‘No’ for no consumption.

Marital status: Marital status was categorized differently in India and Nepal. In India, marital status was divided into five categories based on the response to household questionnaires which were, (i) Never married (ii) Married (iii) Widowed (iv) Divorced, and (v) Not living together. For Nepal, all categories were same except not living together option was not included.

***Household****-****level variables:***

Availability of iodine: Availability of iodine was categorized into three categories; (i) Iodine present, (ii) No iodine, and (iii) No salt in house.

Household wealth quintile: Household wealth quintile was measured by forming the wealth index. The wealth index is a composite measure of a household's cumulative living standard. Households are given scores based on the number and kinds of consumer goods they own, ranging from a television to a bicycle or car, and housing characteristics such as source of drinking water, toilet facilities, and flooring materials. These scores are derived using principal component analysis. National wealth quintiles are compiled by assigning the household score to each usual (de jure) household member, ranking each person in the household population by his or her score, and then dividing the distribution into five equal categories, each comprising 20% of the population:

*A­_­_­_i_ = ∑­_k_ [*$\frac{a_{ik}-\bar{a}_{k}}{S_{k}}$*]*

where $a_{ik}$ is the value of assets *k* for household *i*, $\bar{a}_{k}$ is sample mean, and $s_{k}$ is sample standard deviation.

***Community***-***level variables:***

Place of residence: Place of residence was stratified into two; urban and rural residences.

Region of residence: Region of residence was categorized according to the legal categorization by constitution of both countries. In India, first it was categorized into six geographical regions: North, Central, East, Northeast, West, and South. Further, it was divided into 36 states (33 states and 3 union territories considered as states). In Nepal, region was first divided into three ecological zones; Terai, Hills, and Mountains and further it was divided into seven provinces.

**Supplementary Method 4. Statistical Analysis**

**Multi-level logistic regression with random intercept**

Survey data are generally hierarchical in nature. These data are nested in more than one category (for example states, provinces etc.). Thus, to consider the hierarchical effects multi-level regression was used. MLM accounts for different levels of aggregation that is present in data.

***Logit(odds) = B_00_ + (B_10_ + u_1j_) ∗ x_ij_ + B_01_∗ X_j_ + B_11_∗ x_ij_ ∗ X_j_ + u_0j_***

Where, ***B_00_*** is fixed intercept. ***B_10_*** is fixed slope of level-1 variable.

***u_1j_*** and ***u_0j_*** is residual term associated with level-1 predictors ***x_ij_*** _­_level-2 predictors ***X_j_*** respectively.

***B_01_*** fixed/ random slope of level-2 variables ***X_j._***

***B_11_*** is the coefficient estimate associated with the cross-interaction of level-1 and level-2 predictors.

**Slope index of inequality (SII)**

SII is a complex, weighted measure of inequality that represents the absolute difference in estimated values of a health indicator between the most advantaged and disadvantaged, while taking into consideration all the other subgroups-using a regression model. SII is obtained via regression of the mean health variable on mean relative rank variable. To calculate relative rank the social groups are first ranked from lowest to highest and the cumulative proportion of population is assigned for each group. The midpoint of the population proportion for the most deprived socio-economic group is taken as its relative rank. For each consecutive group relative rank is calculated by adding the mid-point of its population proportion to the proportion of the previous groups. _­_For the favourable health intervention indicators, the difference between the estimated values at rank (*ν_1_* ­­) and rank 0 (*ν_0 ­_*) (covering the entire distribution) generates the SII value:

***SII = ν_1 ­_ -ν_0_***

And the regression equation is specified as follow:

$\bar{\boldsymbol{y}}\boldsymbol{=}\boldsymbol{\beta}_{\boldsymbol{0}}\boldsymbol{+}\boldsymbol{\beta}_{\boldsymbol{1}}\bar{\boldsymbol{R}}$***_j_***

Where *j* indexes social group, $\bar{y}$ is the average health status and $\bar{R}$*_j­_ _­_*the average relative rank. $\beta_{0}$ is the estimated health status of a person at the bottom of the social group hierarchy (a person whose relative rank $\bar{R}$*_j ­_*in the social distribution is zero) and $\beta_{1}$is the difference in average health status between the person with relative rank zero ($\bar{R}$*­_j_* = 0), and person with relative rank 1 ($\bar{R}$ = 1) in the cumulative proportion of the population rank from 0 to 1. For grouped data, use of ordinary least squares regression would result in heteroskedasticity of the error term (i.e. when different groups ‘error have different variances), which violets the assumption of linear regression, where the weights are proportional to the population size of the socioeconomic groups.

SII can take positive as well as negative values. SII takes value zero if there is no inequality. Greater absolute values indicate the higher level of inequality.

**Relative index of inequality (RII)**

RII is a complex weighted measure of inequality that shows the health gradient across multiple subgroups with natural ordering, on a relative scale. It indicates the extent to which a health indicator is concentrated among the disadvantaged or the advantaged. SII discussed above is a measure of absolute disparity, whereas, RII measure the relative disparity. Dividing the estimated SII by the mean population health gives RII.

***RII = SII / µ =*** $\boldsymbol{\beta}_{\boldsymbol{1}}$***/µ***

Where *µ* is mean population health and the SII estimate of $\beta_{1}$ from the regression that generates the SII. RII takes only positive values. If there is no inequality, RII takes the value one. RII value larger than one indicates a concentration of the indicator among the advantaged and values smaller than one indicates concentration of the indicator among the disadvantaged.

**Concentration index (CnI)**

Concentration index is a relative measure of inequality that indicates the extent to which a health indicator is concentrated among the disadvantaged or the advantaged. It is interpreted in reference to the concentration curve (CC). CC plots the cumulative % of health variable against the cumulative % of population ranked for a specific variable of interest such as socioeconomic status or education. CnI is defined as twice the area between the CC and the line of equality (the 45°line.). It quantifies the degree of inequality in a specific health variable.

$$\boldsymbol{C}\boldsymbol{=1-2}\int_{\boldsymbol{0}}^{\boldsymbol{1}} \boldsymbol{L}_{\boldsymbol{h}}\left( \boldsymbol{p} \right)\boldsymbol{dp}$$

where *C* is concentration index and $L_{h}\left( p \right)$ is concentration curve. The index is bounded between -1 and 1.

For a discrete living standards variable, it is derived as

$$\boldsymbol{C=}\frac{\boldsymbol{2}}{\boldsymbol{N}_{\boldsymbol{\mu}}}\sum_{\boldsymbol{i=1}}^{\boldsymbol{n}} \boldsymbol{h}_{\boldsymbol{i}}\boldsymbol{r}_{\boldsymbol{i}}\boldsymbol{-1-}\frac{\boldsymbol{1}}{\boldsymbol{N}}$$

Where $h_{i}$is the health sector variable, *µ* is its mean, and $r_{i}$= *i / N* is the fractional rank of individual *i* in the living standard distribution, with *i =* 1 for the poorest and *i =* N for the richest. CnI can be derived in terms of covariance between health variable and the fractional rank in the living standard distribution

$$\boldsymbol{C=}\frac{\boldsymbol{2}}{\boldsymbol{\mu}}\boldsymbol{cov(h,r)}$$

CnI is zero if there is no inequality, hence overlapping line of equality. It takes negative value when the curve lies above the line of equality, indicating disproportionate concentration of the health variable among the disadvantaged population. Positive value of CnI indicate that the curve lies below the line of equality and the concentration of health is among the advantaged population.

**Decomposition of concentration index (dCnI)**

The health concentration index can be decomposed into the contribution of individual factors to income-related health inequality, in which each contribution is the product of the sensitivity of health with respect to that factor and the degree of income-related inequality in that factor.

$$\boldsymbol{C=}\sum_{\boldsymbol{k}} \mathbf{(}\boldsymbol{\beta}_{\boldsymbol{k}}\boldsymbol{x}_{\bar{\boldsymbol{k}}}\boldsymbol{/\mu)}\boldsymbol{C}_{\boldsymbol{k}}\boldsymbol{+G}\boldsymbol{C}_{\boldsymbol{\varepsilon}}\boldsymbol{/\mu}$$

where $\mu$ is mean of y, $x_{\bar{k}}$is the mean of $x_{k}$, $C_{k}$is the concentration index for $x_{k}$ (defined analogously to *C*), and $GC_{\varepsilon}$is the generalized concentration index for the error term (ɛ). This equation shows that *C* equal to a weighted sum of the concentration indices of the *k* regressors.

Decomposition of concentration index measures the contribution of individual factors in concentration index. All the analyses were performed using Stata version 15.1/SE.
